# Installation of an organocatalyst into a protein scaffold creates an artificial Stetterase†

**DOI:** 10.1039/d4cc05182c

**Published:** 2024-10-25

**Authors:** Alice MacAulay, Eva Klemencic, Richard C. Brewster, Süleyman Mert Ünal, Evangelia Notari, Christopher W. Wood, Amanda G. Jarvis, Dominic J. Campopiano

**Affiliations:** a School of Chemistry, University of Edinburgh, Joseph Black Building, King's Buildings Edinburgh EH9 3FJ UK Dominic.Campopiano@ed.ac.uk Amanda.Jarvis@ed.ac.uk; b School of Biological Sciences, University of Edinburgh, Roger Land Building, King's Buildings Edinburgh EH9 3FF UK

## Abstract

Using a protein scaffold covalently functionalised with a thiamine-inspired N-heterocyclic carbene (NHC), we created an artificial Stetterase (ArtiSt) which catalyses a stereoselective, intramolecular Stetter reaction. We demonstrate that ArtiSt functions under ambient conditions with low catalyst loading. Furthermore, activity can be increased >20 fold by altering the protein scaffold.

## Abstract 2

Using a protein scaffold covalently functionalised with a thiamine-inspired N-heterocyclic carbene (NHC) we created an artificial Stetterase (ArtiSt) which catalyses a stereoselective, intramolecular Stetter reaction.

## Graphical abstract

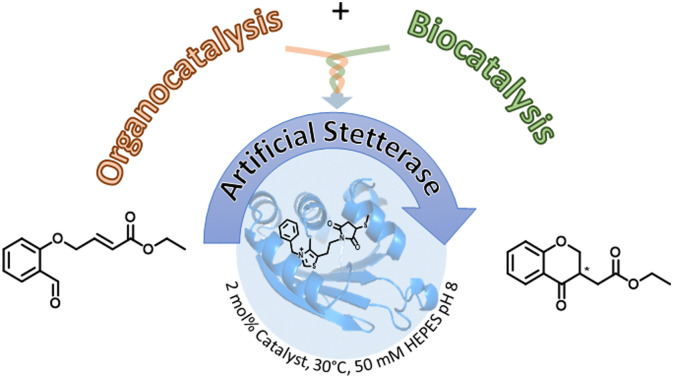

The synthesis of 1,4 dicarbonyl compounds remains a challenging transformation in synthetic chemistry due to the innate polarity mismatch of carbonyl fragments when forming even-numbered dicarbonyls.^1^ One synthetic route, reported by Stetter in 1973, uses a nucleophilic catalyst to catalyse C–C bond formation between an aldehyde and an α,β-unsaturated carbonyl.^2^ Such reactions can be catalysed by N-heterocyclic carbenes (NHCs), a class of compounds inspired by the vitamin B derived cofactor thiamine pyrophosphate (TPP, Fig. 1A). TPP is composed of a catalytic thiazole moiety linked to a pyrimidine ring and a pyrophosphate handle important for non-covalent protein: cofactor interactions. The seminal work of Breslow described the innate catalytic properties of TPP and has inspired subsequent generations of researchers in organocatalysis.^3–6^ A simple thiazolium-based NHC precursor was first used for a Stetter reaction in 1976,^7^ since then, the development of chiral NHCs for enantioselective reactions has led to a rapidly growing pool of catalysts, covered by several reviews.^1,6,8,9^ Whilst NHCs are used as enantioselective organo-catalysts such as for an intramolecular Stetter reaction (Fig. 1B),^10^ they are also versatile ligands for numerous transition metal catalysts.^9^ An example, inspired by biology, is the synthesis of a benzylthiazolium pyrophosphate gold(i)–carbene complex, which was found to catalyse a hydroalkoxylation of an allene in buffered aqueous conditions at pH 7.0.^11^ Chiral NHCs can require complex synthesis, however the discovery of natural TPP dependant Stetterases such as MenD^12^ and PigD^13^ from the menaquinone and prodigiosin biosynthetic pathways respectively, offer an alternative biocatalytic solution.^14^ The field of NHC organocatalysis has now surpassed the reaction scope of TPP-dependant enzymes and as such it would be interesting to conjugate these novel NHCs to a protein and direct enantioselective catalysis^15,16^

**Fig. 1:**
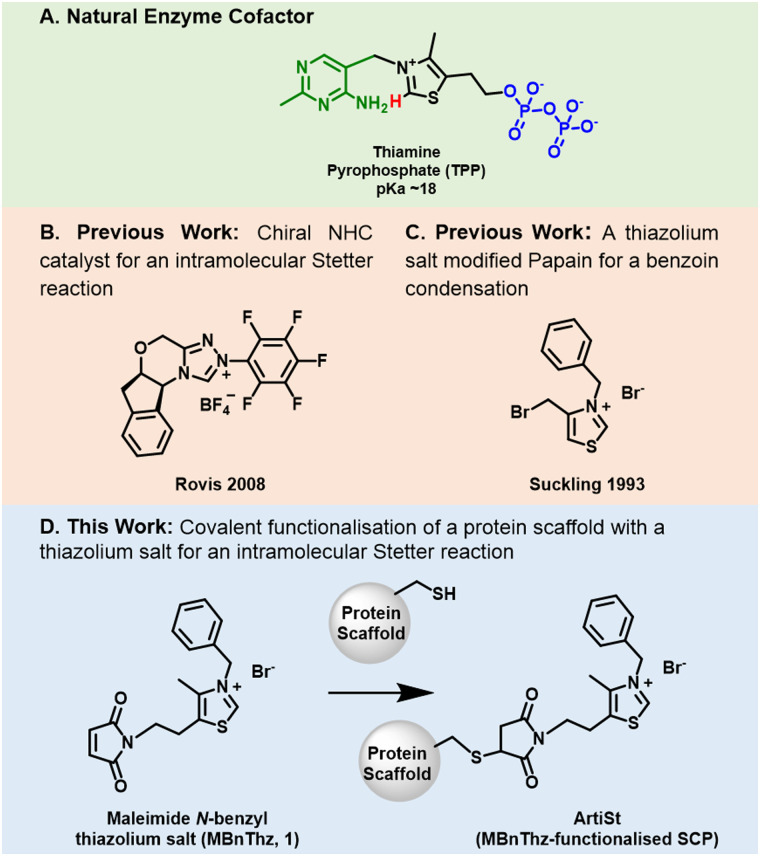
(A) The chemical structure of the enzyme cofactor thiamine pyrophosphate (TPP) showing the C2-proton (red), pyrophosphate (PPi, blue) and pyrimidine (PYR, green). (B) The structure of a chiral triazolium salt used by Rovis in 2009 to catalyse an intramolecular Stetter reaction. (C) The structure of *N*-benzyl-4-bromomethylthiazolium bromide prepared by Suckling in 1993 to modify papain for use in an intramolecular benzoin condensation. (D) A scheme of this work showing the structure of the thiazolium salt (MBnThz, 1) and its functionalisation of a cysteine-containing protein scaffold to create the artificial Stetterase (ArtiSt).

In solution TPP can catalyse reactions such as the decarboxylation of α-keto acids and C–C bond formations albeit at a low rate. However, when TPP is located in the active site of an enzyme, its activity is increased by many orders of magnitude (*e.g.* decarboxylase activity increased by 3 × 10^12^ for yeast pyruvate decarboxylase, PDC)^17^ delivering products with high enantioselectivity. Few natural “Stetterases” exist and all display a limited substrate scope when compared with other TPP-dependent enzymes, which when combined with difficulties in producing soluble, recombinant protein, has impeded their application in biocatalysis.^18,19^ The possibility to repurpose other TPP-dependant enzymes for the synthesis of 1,4 dicarbonyls was highlighted recently.^20^ In this paper the authors examined wild type TPP-containing enzymes (*Pseudomonas fluorescens* benzaldehyde lyase (PfBAL), MenD, ApPDC and a benzylformate decarboxylase (BFD)) and used molecular dynamics (MD) simulations to select PfBAL as a potential Stetterase.^20^ The natural promiscuity of PfBAL was exploited to catalyse an intramolecular reaction with 10 out of 13 substrates to give the corresponding Stetter products with 60–99% yield and e.r.'s ranging from 89 : 11 to 99 : 1.^20^

Our approach was to make use of the catalytic power of NHCs and embed them in a suitably functionalised protein environment to create an artificial “biohybrid” catalyst. This concept of pairing a chemical catalyst and a genetically encoded tuneable biocatalyst has received great attention in recent years and shows promise in being able to deliver synthetically useful tools.^21–24^

Early studies by Hilvert and Breslow showed that thiazolium-modified, cyclodextrin protein mimics catalysed benzoin condensations.^25,26^ Later, Suckling revealed that bioconjugation of an NHC (*N*-benzyl-4-bromomethylthiazolium bromide, Fig. 1C) to the protease papain could be used to catalyse a benzoin condensation of 6-oxoheptanal.^27,28^ The methods available at the time and the fact that papain was obtained from papaya extract rather than through recombinant expression, made it difficult to fully explore the mechanistic details of this important proof of concept work. Recent advances in computational techniques now enable better modelling of active sites,^29^ more accurate docking of substrates^30,31^ and the design of *de novo* protein scaffolds^32^ with some designs going beyond the 20 canonical amino acids to enable novel enzyme-reaction with enhanced catalytic properties.^22,33,34^

Inspired by the work of Suckling^27^ we have developed a genetically-encoded, tuneable, artificial Stetterase by placing a thiazole-based NHC specifically into a protein scaffold (Fig. 1D). As an initial proof of concept we focused on an intramolecular Stetter reaction (Scheme 1 and Fig. S19, ESI†)^35,36^ which requires a single substrate that undergoes cyclisation by formation of a C–C bond. For our protein scaffold, we chose to use the human steroid carrier protein 2L (hSCP) containing a unique, reactive cysteine mutation, A100C, since it has been successfully used to create several artificial enzymes for reactions as diverse as hydroformylation^37,38^ and both photocatalytic^39^ and transition metal oxidation.^40^ Structural analysis of hSCP A100C (PDB code: 6Z1W) revealed it has a hydrophobic tunnel of ∼18 Å long and ∼9 Å wide which should be sufficient to accommodate a maleimide-linked NHC as well as the intramolecular Stetter substrate (Fig. S27, ESI†). We opted to prepare a novel thiazole-based NHC, MBnThz, 1*via* a convergent 5 step synthesis using inexpensive starting materials (Fig. 1D and Fig. S1, ESI†). This was used to functionalise recombinant hSCP A100C purified from *E. coli via* a selective maleimide-thiol reaction. Mass spectrometry analysis showed successful mono-functionalisation of the protein with 100% conversion (Fig. S14 and Table S2, ESI†). This functionalised protein was then screened as a catalyst and pleasingly conversion of substrate 2 to Stetter product 3 was observed by HPLC (Table 1 and Fig. S24c, ESI†). To our knowledge, this represents the first time that a NHC-modified protein has displayed Stetterase activity.

**Scheme 1:**
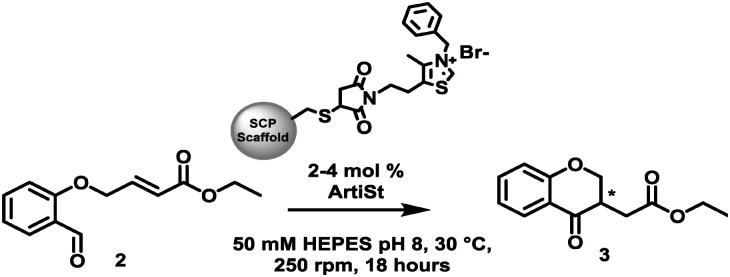
The intramolecular Stetter reaction catalysed by different thiazolium functionalised SCP scaffolds (ArtiSt).

**Table 1:** Activity of functionalised SCP scaffold for the intramolecular Stetter reaction (2.5 mM 2, 50 mM HEPES pH 8, 30 °C, 250 rpm, 18 hours)

Protein	Conc. (μM)	Mono-functionalisation^a^ (%)	Yield^b^ of 3 (%)	TTN^c^
MBnThz (control)	100	—	<1	<0.1
TTSCP L102C	100	Unmodified	<1	<0.1
hSCP A100C	100	98	4.4	1.1
TTSCP W83C	100	<5	—	—
TTSCP L112C	100	85	<1	<0.1
TTSCP ΔDSB L102C	100	90	7.8	2.2
TTSCP L102C	100	50	23	22^d^

a Functionalisation percentage determined by LC-MS protein ion relative rations.

b HPLC yield.

c Total turn over number.

d Based on thiazolium-modified scaffold.

Having demonstrated that the organocatalyst-functionalised hSCP A100C scaffold is active we looked to improve the conversion. However, despite attempts at optimisation, this initial scaffold was not carried forward due to protein precipitation (pI 9.24) when reactions were carried out at pH 8.0. A screen of reaction conditions (pH 6.0–8.0) showed less precipitation at lower pH but pH 8.0 was required for catalysis as negligible activity was observed at lower pHs (Fig. S23, ESI†). However, SCPs are found in many different species that display desirable properties. The SCP from *Thermus thermophillus* (TTSCP, PDB: 2CX7)^41^ was identified as a potential scaffold for further development of the Stetterase due to its acidic pI (4.98) and thermostability (*T*_m_ > 95 °C, Fig. S32, ESI†). One issue we considered when changing scaffold is the size of the hydrophobic pocket which varies across the SCP superfamily (Table S3, ESI†). Docking of the proposed Breslow intermediate (Fig. S27, ESI†) into this scaffold using Autodock Vina^31^ suggested that despite being approximately half the size of the hSCP cavity, it was large enough to accommodate both the NHC organocatalyst, 1 and the Stetter substrate, 2. In order to allow functionalisation of the scaffolds with 1, a free cysteine residue was required to be introduced into the TTSCP scaffold. To select potential positions for the cysteine, docking studies of 1 into TTSCP (PDB; 2CX7 Chain A) generated a number of poses (Table S4, ESI†). From these residues W83, L102 and L112 were chosen based on their proximity to the maleimide (<5 Å) and their inward orientation within the cavity. The soluble, recombinant scaffolds TTSCP W83C, L102C and L112C were expressed and purified from *E. coli* in higher yields (∼20–50 mg L^−1^) than the hSCP A100C (Table S2, ESI†).

To obtain the desired modified TTSCP scaffolds, the proteins were functionalised by addition of MBnThz, 1 and conversion monitored by ESI-MS (Fig. S15–S17, ESI†). We found TTSCP L112C was the easiest to functionalise but some over functionalisation did occur (85% mono, 10% di, 5% tri). In contrast, TTSCP W83C and TTSCP L102C were much more challenging and required harsher conditions that caused significant protein precipitation.

After optimisation, MS analysis revealed ∼50% functionalisation of L102C whereas only <10% functionalisation of W83C was achieved, so this construct was not carried forward. Unlike the hSCP scaffold, the TTSCP protein also contains two cysteine residues (Cys13 and Cys60) which form a disulfide bond. Whilst preparing these scaffolds we observed multiple adducts of 1 which we ascribed to modification of the native cysteine thiol residues. This issue was resolved by mutating both Cys13 and Cys60 residues to alanine to prepare a scaffold with the disulfide bond (DSB) removed, named TTSCP ΔDSB L102C. Recombinant TTSCP ΔDSB L102C was obtained in good yield (Table S2, ESI†) and CD analysis revealed that the stability of this scaffold was not compromised to a great extent by removal of this structural feature (*T*_m_ 79 °C, Fig. S34, ESI†). This was also supported by extensive molecular dynamics simulations which consistently showed that removal of the disulfide bond and/or addition of the MBnThz did not disrupt the structure of the scaffolds (Fig. S29–S31, ESI†). The TTSCP ΔDSB L102C was mono functionalised to >90% with a single MBnThz 1 and only trace amounts of over modification was observed (Fig. S18, ESI†), supporting the hypothesis that the disulfide bond of TTSCP was the source of the over functionalisation. Moreover, less precipitation was observed during modification.

The Stetterase activity of the three MBnThz-modified scaffolds (TTSCP L112C, TTSCP L102C and TTSCP ΔDSB L102C) was analysed. We were disappointed to see the easiest to functionalise, TTSCP L112C, displayed no catalytic activity above background, although this was useful in serving as a negative control (Table 1 and Fig. S24d, ESI†). However, we were pleased to observe that both of the functionalised TTSCP L102C scaffolds displayed Stetterase activity (Table 1 and Fig. S24e, f, ESI†). The modified TTSCP ΔDSB L102C displayed a 2-fold increase in activity over the previous hSCP A100C scaffold (2.2 turnovers, 8% yield, Table 1 and Fig. S24f, ESI†).

To our surprise, when we used the modified disulfide-containing scaffold TTSCP L102C, the activity increased ∼20 fold (22 turnovers, 23% yield, Table 1 and Fig. S24e, ESI†). The finding that this is the best catalyst is especially gratifying since this scaffold was the most difficult to selectively functionalise (∼50% functionalisation) and as such the effective catalyst concentration is half (50 μM, 2 mol%) compared to the other catalysts. Control reactions using unmodified TTSCP L102C confirmed the observed Stetterase activity was due to the thiazolium modification (Table 1). Yields were comparable to those achieved with functionalised thiazolylalanine peptides in organic solvents^42^ but unsurprisingly still fell short of those achieved by the TPP dependant enzyme PfBAL.^20^ In an attempt to understand why TTSCP L102C was the catalytically superior mutant, particularly compared to the TTSCP ΔDSB L102C variant, AlphaFold2^29^ was used to generate models of the two L102C scaffolds. Analysis of these models using CastP^43^ revealed the calculated cavity volumes and size of openings of the two scaffolds to be different (Table S3, ESI†). It is worth noting that open and closed conformations of the TTSCP scaffold are observed in the two chains present in the crystal structure (PDB: 2CX7), whereas the NMR structure (PDB: 1WFR) only displays a closed conformation. From modelling alone, it is difficult to determine why the two functionalised scaffolds display such different catalytic activities, however it seems likely to be linked to both conformational changes and protein dynamics, rather than the reduced stability resulting from removal of the disulfide bond.

Importantly, we also examined the stereoselectivity of the reaction and the NHC-modified TTSCP L102C scaffold displayed a modest, but clear 5% ee. of Stetter product 3 (Fig. S25, ESI†). This exciting observation allows comparison with the PfBAL enzyme that catalyses a stereoselective intramolecular Stetter reaction that converts substrate 2 to product 3 with 98% ee. It is also interesting to note that our functionalised biocatalyst displays a preference for the formation of the (*R*)-enantiomer, whilst PfBAL catalysed formation of the (*S*)-enantiomer. An important next step will be the determination of the 3D structure of this novel catalyst to identify where the NHC sits within the cavity, predict residues involved in catalysis and enable semi-rational engineering to improve activity and stereoselectivity.

We set out to combine the power of organocatalysis with the selectivity of biocatalysts and pave the way for creating tuneable biohybrid catalysts for organic synthesis.^23^ To that end, we show it is possible to create an artificial Stetterase (which we name “ArtiSt”) by bioconjugation of an NHC to an inert protein scaffold. We highlight how important the choice of scaffold is and how even without applying protein design/evolution, scaffold choice can lead to a ∼20 fold increase in catalytic activity. Structural studies and enzyme engineering are ongoing to improve the activity and stereoselectivity of this catalyst. It is appreciated that there are technical challenges associated with selective and efficient attachment of the NHC to the chosen scaffold. One way to overcome this is to use genetic code expansion to incorporate an NHC based amino acid as an alternative to bioconjugation.^44^ Nevertheless, the successful demonstration that the TTSCP/thiazole pairing is catalytically active in aqueous conditions allows us to screen different protein/NHC combinations that can catalyse a range of chemical reactions. Furthermore, as an alternative to the thiazole-derived from TPP, it is hoped that this concept could be used to harness the diverse chemistry and catalytic repertoire of NHC organocatalysts once they are incorporated into the chiral environment of a protein.

Authors acknowledge support from the BBSRC (EastBio, BB/M010996/1 and BB/X003027/1) and UK Research and Innovation (MR/S017402/1 and MR/X011127/1).

## Data availability

All raw data (*e.g.* NMR, mass spectrometry, CD and protein modelling) is available upon request from the corresponding author.

## Conflicts of interest

There are no conflicts to declare.

## Supplementary Material

CC-060-D4CC05182C-s001
