# Supplementary material for: Installation of an organocatalyst into a protein scaffold creates an artificial Stetterase
Source: Chem Commun (Camb). 2024 Oct 25;60(93):13746–9. doi: 10.1039/d4cc05182c (PMC11533139; doi:10.1039/d4cc05182c)
Supplement: CC-060-D4CC05182C-s001 [file CC-060-D4CC05182C-s001.pdf]

Supplementary Information (SI) for ChemComm.
This journal is © The Royal Society of Chemistry 2024

Supplementary Information

Installation of an organocatalyst into a protein scaffold creates an artificial Stetterase.

Alice MacAulay[a], Eva Klemencic[a], Richard C. Brewster [a,c], Süleyman Mert Ünal[b], Evangelia Notari[a, b], Christopher W. Wood[b], Amanda G. Jarvis[a]*, Dominic J. Campopiano[a]*

Author Address:

[a] School of Chemistry, Joseph Black Building, The University of Edinburgh, King's Buildings, Edinburgh, EH9 3FJ, United Kingdom.

[b] School of Biological Sciences, The Roger Land Building, The University of Edinburgh, Edinburgh, EH9 3FF, United Kingdom.

[c] Current Address: AstraZeneca, Pharmaceutical Sciences, Advanced Drug Delivery, Etherow Building, Macclesfield, SK10 2NA.

*Corresponding authors: Contact Dominic.Campopiano@ed.ac.uk and Amanda.Jarvis@ed.ac.uk

Contents

Abbreviations 3

General remarks 4

Synthesis of MBnThz (1) 5

Plasmid sequences 8

Protein Expression and Purification 12

Protein functionalisation 15

Protein LC-MS..17

Intramolecular Stetter reaction..28

Structural analysis 35

Docking..36

TTSCP melting temperature measurements 45

NMR and MS Data 49

Abbreviations

ArtiSt	Artifical Stetterase
BAL	Benzaldehyde lyase
BFD	Benzylformate decarboxylase
CD	Circular dichroism
HPLC	High pressure liquid chromatography
MBnThz	N-Benzyl-5-(2-maleimidoethyl)-4-methyl-thiazolium bromide
MS	Mass spectrometry
NHC	N-heterocyclic carbene
PDC	Pyruvate Decarboxylase
SCP	Steroid carrier Protein
TPP	Thiamine Pyrophosphate

General remarks

NMR ^{1}H, ^{2}H and ^{13}C NMR spectra were obtained in deuterated solvent using the Bruker Ascend 500 MHz spectrometer. Data for ^{1}H NMR are reported in the conventional form: chemical shift (δ ppm), multiplicity (s = singlet, d = doublet, t = triplet, q = quartet, m = multiplet, br = broad), coupling constant (Hz), and integration.

Mass Spectrometry (MS) Mass spectrometry analysis of small molecules was completed using a Bruker microTOF instrument using electrospray ionisation (ESI+). Protein LC ESI-MS was completed using a Synapt G2-Si Q-TOF (Waters) instrument with Phenomenex C4 3.6 µm column.

Protein Purification All protein purification steps were completed on AKTA Go or AKTA Start (Cytiva Lifesciences) using a F9-R fraction collector.

Centrifugation Thermoscientific Heraeus Multifuge X3R was used for harvesting cells and concentrating protein with 8 × 50 mL rotor and 6 × 250 mL rotor at 4 °C. Benchtop centrifuge VWR Microstar 17 and was used at rt as required.

HPLC Both chiral and C18 reverse phase HPLC methods were carried out using a Shimadzu instruments HPLC fitted with an autosampler (SIL-20A HT), pump (LC-20AT), a UV/visible detector (SPD-20A) and a column oven (CTO-20A). For reverse phase analysis a Luna 5u C18 RP-Phenomenex column (100 Å, 250 x 4.6 mm) was used and for chiral analysis a CHIRALPAK® IB N-5 analytical column (5 µm, 250 × 4.6 mm) column was used.

Synthesis of MBnThz (1)

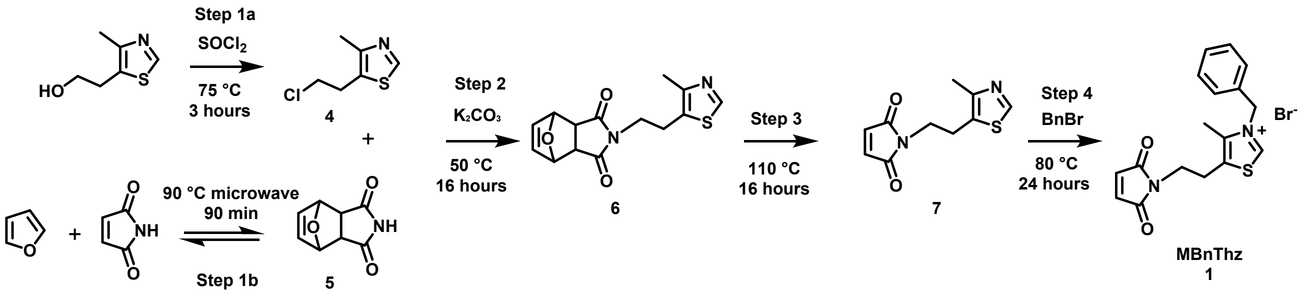

***Figure S1**: Overview of synthetic route to **1** MBnThz*

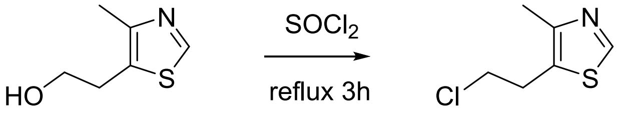

4

Figure S2** Synthesis of **4: 2-(4-methylthiazol-5-yl)ethanol (1.20 mL , 10 mmol) was added to thionyl chloride (10 mL) and the mixture was stirred at reflux for 3 hours. Thionyl chloride was removed by rotary evaporation and the product was washed with toluene and dried under vacuum. The product was used without further purification.

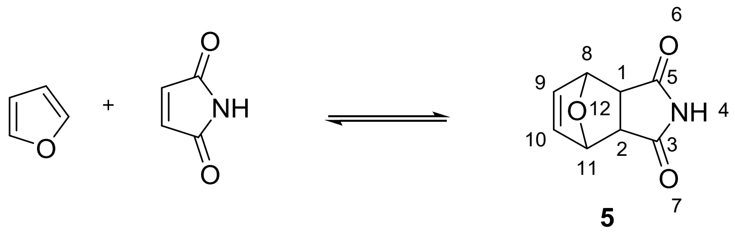

5

Figure S3** Synthesis of **5: Maleimide (10 mmol, 970 mg, 1 eq) was dissolved in dH2O (11.4 mL) in a 35 mL microwave tube. Furan (17 mmol, 1.24 mL, 1.7 eq) was added. The reaction was heated to 90 °C in a microwave reactor for 90 min then cooled, filtered and product **5** was washed with dH_2O then dried under vacuum (white powder, 60 % yield, 1 g).

^{1}H NMR (500 MHz, Methanol-d_4) δ 6.55 (t, J = 1.0 Hz, 2H), 5.18 (t, J = 1.0 Hz, 2H), 2.92 (s, 2H) ^{13}C NMR (126 MHz, Methanol-d_4) δ 178.4, 136.2, 80.8, 48.7. Mass calcd for $[C_8H_7O_4N_1O_3Na]^+$ ESI $[M+]^+$ requires m/z =188.0318 ,found m/z = 188.0315. In agreement with literature [1].

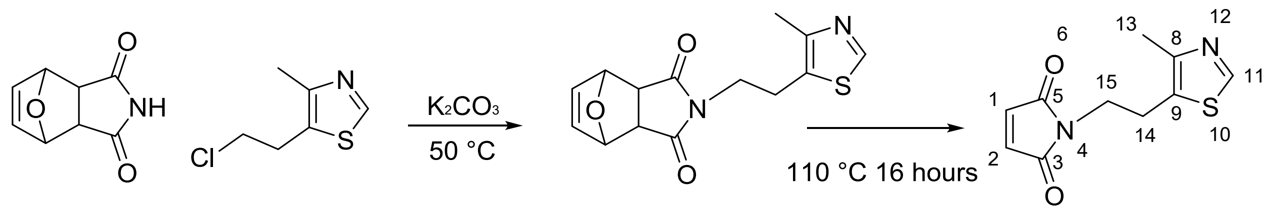

4 5 6 7

Figure S4 *Synthesis of* ***7***: Compound **4** (3 mmol, 500 mg, 1 equiv) and potassium carbonate (15 mmol, 2.1 g, 5 equiv) were dissolved in DMF (10 mL) under argon and heated to 50 °C for 1 hour. Crude **4** (3.6 mmol, 581 mg, 1.2 equiv) was added and the reaction was stirred at 50 °C for 18 hours. The reaction was cooled, filtered and DMF was removed under reduced pressure. The crude product was dissolved in ethyl acetate (50 mL) and washed with water (3X 10 mL). The organic extract was dried with $MgSO_4$, filtered and concentrated under reduced pressure. The crude product was dissolved in ethyl acetate, filtered through a silica pad and concentrated under reduced pressure. The colourless oil **6** was dissolved in toluene 10 mL and refluxed at 110 °C for 18 hours. Toluene was removed under reduced pressure to give **7** in 242 mg, 30% yield.

^{1}H NMR (500 MHz, Chloroform-*d*) δ 8.60 (s, 1H^{11}), 6.72 (s, 2$H^{1,2}$), 3.77 (t, *J* = 7.2, 2H^{15}), 3.11 (t, *J* = 7.2 Hz, 2H^{14}), 2.41 (s, 3H^{13}).^{13}C NMR (126 MHz, $CDCl_3$) δ 170.3(2$C^{3,5}$), 149.9 (2$C^{8,11}$), 134.2 (2$C^{1,2}$), 126.6 (1C^{9}), 38.4(C^{15}), 25.0 (C^{14}), 14.8 (C^{13}). Mass calcd for $[C_{10}H_{10}N_2O_2S]^+$ requires ESI $[M+H]^+$ *m/z*= 223.0536, found *m/z* = 223.0535 (ESI) .MP 83-84 °C. IR : 3068.95, 1437.70, 1410.56, 1367.71, 1150.59, 902.05, 829.91

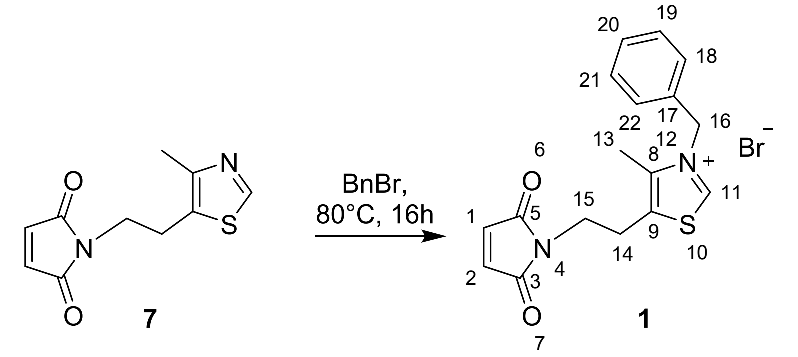

Figure S5 *Synthesis of* ***1*** *(N-Benzyl-5-(2-maleimidoethyl)-4-methyl-thiazolium bromide)*: Compound **7** (1 mmol, 222 mg, 1 eq) and benzylbromide (2 mmol, 238 uL, 2 eq) were dissolved in acetonitrile (5 mL) and refluxed at 80 °C for 24 hours. Acetonitrile was removed under reduced pressure. The product was triturated in diethyl ether and isolated via filtration to give a sticky/amorphous off-white solid,282 mg, 72% yield.

^{1}H NMR (500 MHz, Chloroform-*d*) δ 11.64 (s, 1H^{11}), 7.45 – 7.36 (m, 3H^{18,20,22}), 7.36 – 7.30 (m, 2H^{19,21}), 6.69 (s, 2H^{1,2}), 6.13 (s, 2H^{16}), 3.81 (t, *J* = 6.7 Hz, 2H^{15}), 3.19 (t, *J* = 6.7 Hz, 2H^{14}), 2.39 (s, 3H^{13}).^{13}C NMR (126 MHz, $CDCl_3$) δ 170.1 (2C^{3,5}), 159.2(1C^{11}), 143.4 (1C^{8}), 134.3 (1C^{1}), 134.0 (1C^{2}), 131.6 (1C^{9}), 129.5(3C^{22,20,18}), 129.4(2C ^{21,19}), 128.1(1C^{9}), 57.6 (1C^{15}), 37.4 (1C^{16}), 26.1 (1C^{14}), 12.3 (1C^{13}).Mass calcd for $[C_{17}H_{17}N_2O_2S]^+$ requires ESI $[M+H]^+$ *m/z* = 313.1010, found *m/z* = 313.1009.MP 179-180°C.IR: 3033.24, 1497.70, 1373.42, 1333.43, 1158.45, 951.33, 913.47, 834.91, 692.78.

Plasmid sequences

Human SCP-2L-A100C

Gene Sequence

ATGTCGTACTACCATCACCATCACCATCACGATTACGATATCCCAACGACCGAAAACCTGTATTTTCA
GGGCGCCATGGAGGGAGGGAAGCTTCAGAGTACCTTTGTATTTGAGGAAATAGGACGCCGCCTAA
AGGATATTGGGCCTGAGGTGGTGAAGAAAGTAAATGCTGTATTTGAGTGGCATATAACCAAAGGCG
GAAATATTGGGGCTAAGTGGACTATTGACCTGAAAAGTGGTTCTGGAAAAGTGTACCAAGGCCCTG
CAAAAGGTGCTGCTGATACAACAATCATACTTTCAGATGAAGATTTCATGGAGGTGGTCCTGGGCAA
GCTTGACCCTCAGAAGGCATTCTTTAGTGGCAGGCTGAAGTGCAGAGGGAACATCATGCTGAGCCA
GAAACTTCAGATGATTCTTAAAGACTATGCCAAGCTCTGA

Protein Sequence

MSYYHHHHHHDYDIPTT**ENLYFQ**GA**M**EGGKLQSTFVFEEIGRRLKDIGPEVVKKVNAVFEWHITKGGNI
GAKWTIDLKSGSGKVYQGPAKGAADTTIILSDEDFMEVVLGKLDPQKAFFSGRLK**C**RGNIMLSQKLQMIL
KDYAKL

The N-terminal residues in red are the affinity tag and TEV protease sequence (ENLYFQ/G). Residues are numbered from the subsequence Met (bold, underlined). Cys100 is bold underlined.

TTSCP

The SCP gene from *Thermus Thermophilus* was ordered from IDT and codon optimised for *E. coli* K12 with the 5’ and 3’-terminus for pET28b vector insertion via Gibson assembly incorporating a C-terminal His tag. Gibson assembly was performed using HiFi DNA assembly Master Mix at 20 µl.

Gene Sequence

ATGGAGCTTTTCACCGAGGCCTGGGCCCAGGCGTACTGCCGGAAGCTGAACGAGAGCGAGGCCTA
CCGCAAGGCGGCGAGCACCTGGGAGGGCTCCCTGGCCCTCGCGGTGCGCCCGGACCCCAAGGCGG
GGTTCCCCAAGGGGGTGGCCGTGGTCCTGGACCTCTGGCACGGGGCCTGCCGGGGGGCGAAGGCG
GTGGAGGGGGAGGCGGAGGCGGACTTCGTCATTGAGGCCGACCTCGCCACCTGGCAGGAGGTGCT
GGAGGGACGCCTCGAGCCCCTAAGCGCCCTCATGCGGGGACTTTTGGAGCTCAAGAAGGGCACCAT
CGCCGCCCTCGCCCCTTACGCCCAGGCGGCCCAGGAGCTCGTCAAAGTGGCCCGGGAGGTGGCAGA
AAACCTGTATTTTCAGGGCCTCGAGCACCACCACCACCACCACTGA

Protein Sequence

MELFTEAWAQAY**C**RKLNESEAYRKAASTWEGSLALAVRPDPKAGFPKGVAVVLDLWHGA**C**RGAKAVE
GEAEADFVIEADLATWQEVLEGRLEPLSALMRGLLELKKGTIAALAPYAQAAQELVKVAREVAENLYFQG
LEHHHHHH*

Residues Cys13 and Cys60 are bold underlined. The residues at the C-terminus are the affinity tag and TEV protease sequence (ENLYFQ/G). The C-terminal sequence in red is removed.

TTSCP W83C

This construct was created by site directed mutagenesis of TTSCP using the following primers:

TT_W83C_Fw (5’-TGCCaaCCTGTCAGGAGGTGC-3’)

TT_W83C_Rv (5’-GGTCGGCCTCAATGACGAAG-3’).

The PCR protocol for site-directed mutagenesis was designed according to Thermo Scientific Phusion Site-Directed Mutagenesis Kit. The PCR reaction mixture was prepared: PCR Master Mix (1x), primers (200 nM each), template plasmid DNA (total mass of 10 ng), 3 % DMSO and H_2O to 20 µl. The PCR program started with initial denaturation at 98 °C for 30 s. 30 cycles of denaturation at 98 °C for 10 s, annealing at 55 °C for 30 s, followed by extension at 72 °C for 2 min 30 s. The PCR program ends with at 72 °C for 10 min.

Gene Sequence
ATGGAGCTTTTCACCGAGGCCTGGGCCCAGGCGTACTGCCGGAAGCTGAACGAGAGCGAGGCCTA
CCGCAAGGCGGCGAGCACCTGGGAGGGCTCCCTGGCCCTCGCGGTGCGCCCGGACCCCAAGGCGG
GGTTCCCCAAGGGGGTGGCCGTGGTCCTGGACCTCTGGCACGGGGCCTGCCGGGGGGCGAAGGCG
GTGGAGGGGGAGGCGGAGGCGGACTTCGTCATTGAGGCCGACCTCGCCACCTGTCAGGAGGTGCT
GGAGGGACGCCTCGAGCCCCTAAGCGCCCTCATGCGGGGACTTTTGGAGCTCAAGAAGGGCACCAT
CGCCGCCCTCGCCCCTTACGCCCAGGCGGCCCAGGAGCTCGTCAAAGTGGCCCGGGAGGTGGCAGA
AAACCTGTATTTTCAGGGCCTCGAGCACCACCACCACCACCACTGA

Protein Sequence
MELFTEAWAQAY**C**RKLNESEAYRKAASTWEGSLALAVRPDPKAGFPKGVAVVLDLWHGA**C**RGAKAVE
GEAEADFVIEADLAT**C**QEVLEGRLEPLSALMRGLLELKKGTIAALAPYAQAAQELVKVAREVAENLYFQGL
EHHHHHH*

Residues Cys13, Cys60 and Cys83 are bold underlined. The residues at the C-terminus are the affinity tag and TEV protease sequence (ENLYFQ/G). The C-terminal sequence in red is removed.

TTSCP_L102C and TTSCP ΔDSB L102C

Due to the high GC content of the TTSCP genes hindering mutagenesis attempts synthetic gene cloned into pET-28a(+) between NcoI/XhoI restriction sites was purchased from IDT. These constructs were codon optimised for expression in *E.coli* K12 and further optimised for lower GC content

TTSCP_L102C

Gene Sequence
ATGGAATTGTTTACAGAAGCATGGGCACAGGCATATTGTCGCAAGCTGAATGAATCTGAGGCATAC
CGCAAGGCGGCATCGACTTGGGAAGGGTCACTGGCGCTTGCGGTTCGTCCCGATCCTAAAGCCGGG
TTTCCAAAAGGTGTTGCGGTCGTCCTGGATTTATGGCACGGCGCGTGCCGCGGCGCCAAAGCCGTG
GAAGGGGAAGCCGAGGCTGATTTTGTCATTGAAGCCGATTTAGCAACATGGCAGGAGGTTTTAGAG
GGGCGCCTGGAACCATTGTCAGCCCTGATGCGCGGCTTGTGCGAACTTAAGAAGGGCACAATCGCG
GCCTTGGCTCCATACGCCCAAGCGGCTCAGGAGCTGGTAAAAGTGGCCCGCGAGGTGGCCGAAAAC
CTTTATTTCCAGGGACTCGAGCACCACCACCACCACCAC

Protein Sequence
MELFTEAWAQAY**C**RKLNESEAYRKAASTWEGSLALAVRPDPKAGFPKGVAVVLDLWHGA**C**RGAKAVE
GEAEADFVIEADLATWQEVLEGRLEPLSALMRGL**C**ELKKGTIAALAPYAQAAQELVKVAREVAENLYFQG
LEHHHHHH*

Residues Cys13, Cys60 and Cys102 are bold underlined. The residues at the C-terminus are the affinity tag and TEV protease sequence (ENLYFQ/G). The C-terminal sequence in red is removed.

TTSCP ΔDSB L102C

Gene Sequence
ATGGAATTGTTTACAGAAGCATGGGCACAGGCATACGCTCGCAAGCTGAATGAATCTGAGGCATAC
CGCAAGGCGGCATCGACTTGGGAAGGGTCACTGGCGCTTGCGGTTCGTCCCGATCCTAAAGCCGGG
TTTCCAAAAGGTGTTGCGGTCGTCCTGGATTTATGGCACGGCGCGGCACGCGGCGCCAAAGCCGTG
GAAGGGGAAGCCGAGGCTGATTTTGTCATTGAAGCCGATTTAGCAACATGGCAGGAGGTTTTAGAG
GGGCGCCTGGAACCATTGTCAGCCCTGATGCGCGGCTTGTGCGAACTTAAGAAGGGCACAATCGCG
GCCTTGGCTCCATACGCCCAAGCGGCTCAGGAGCTGGTAAAAGTGGCCCGCGAGGTGGCCGAAAAC
CTTTATTTCCAGGGACTCGAGCACCACCACCACCACCAC

Protein Sequence
MELFTEAWAQAYARKLNESEAYRKAASTWEGSLALAVRPDPKAGFPKGVAVVLDLWHGAARGAKAVE
GEAEADFVIEADLATWQEVLEGRLEPLSALMRGL**C**ELKKGTIAALAPYAQAAQELVKVAREVAENLYFQG
LEHHHHHH*

Residue Cys102 is bold underlined. The residues at the C-terminus are the affinity tag and TEV protease sequence (ENLYFQ/G). The C-terminal sequence in red is removed.

TTSCP L112C

The gene for this construct was ordered from IDT and the clone was created in the same way as the WT TTSCP using Gibson assembly.

Gene Sequence
ATGGAGCTTTTCACCGAGGCCTGGGCCCAGGCGTACTGCCGGAAGCTGAACGAGAGCGAGGCCTA
CCGCAAGGCGGCGAGCACCTGGGAGGGCTCCCTGGCCCTCGCGGTGCGCCCGGACCCCAAGGCGG
GGTTCCCCAAGGGGGTGGCCGTGGTCCTGGACCTCTGGCACGGGGCCTGCCGGGGGGCGAAGGCG
GTGGAGGGGGAGGCGGAGGCGGACTTCGTCATTGAGGCCGACCTCGCCACCTGGCAGGAGGTGCT
GGAGGGACGCCTCGAGCCCCTAAGCGCCCTCATGCGGGGACTTTTGGAGCTCAAGAAGGGCACCAT
CGCCGCCTGCGCCCCTTACGCCCAGGCGGCCCAGGAGCTCGTCAAAGTGGCCCGGGAGGTGGCAG
AAAACCTGTATTTTCAGGGCCTCGAGCACCACCACCACCACCACTGA

Protein Sequence
MELFTEAWAQAY**C**RKLNESEAYRKAASTWEGSLALAVRPDPKAGFPKGVAVVLDLWHGA**C**RGAKAVE
GEAEADFVIEADLATWQEVLEGRLEPLSALMRGLLELKKGTIAA**C**APYAQAAQELVKVAREVAENLYFQG
LEHHHHHH*

Residues Cys13, Cys60 and Cys112 are bold underlined. The residues at the C-terminus are the affinity tag and TEV protease sequence (ENLYFQ/G). The C-terminal sequence in red is removed.

Protein Expression and Purification

hSCP A100C

Chemically competent *E. coli* Rosetta 2 (DE3) cells were transformed with pET28_SCPA100C plasmid, plated on LB agar supplemented with kanamycin (30 µg/mL) and chloramphenicol (34 µg/mL) and incubated at 37 °C overnight (~18 h). A single colony was used to inoculate a liquid culture, LB media (100 mL) containing kanamycin (30 µg/mL) and chloramphenicol (34 µg/mL) and grown at 37 °C, 200 rpm, 18 hours. This pre-culture (20 mL) was used to inoculate PB media (1 L) supplemented with kanamycin (30 µg/mL) and chloramphenicol (34 µg/mL) and grown at 37 °C, 200 rpm to an OD_{600nm} ~ 0.6. Temperature was lowered to 16°C and protein expression was induced with the addition of IPTG (0.2 mM). Induced cultures were incubated overnight (~18 h) at 16 °C, 200 rpm. Cells were harvested by centrifugation (8,000 x g 10 min, 4 °C) then resuspended and washed with PBS buffer (40 mL per 1 L culture). Cells were pelleted again (8,000 x g, 30 min, 4 °C), supernatant discarded and the pelleted cells were frozen and stored at -20 °C.

TTSCP constructs

Chemically competent *E. coli* BL21 (DE3) cells were transformed with the TTSCP_pET28 plasmid for each mutant. Cells were plated on LB agar supplemented with Kanamycin (30 µg/mL) and incubated at 37 °C overnight (~18 h). A single colony was used to inoculate LB media (100 mL) containing kanamycin (30 µg/mL) and grown at 37 °C, 200 rpm, 18 hours. This pre-culture (20 mL) was used to inoculate LB media (1 L) supplemented with kanamycin (30 µg/mL) and grown at 37 °C, 200 rpm to an OD_{600nm} ~ 0.6. Temperature was lowered to 30°C and protein expression was induced with the addition of IPTG (0.5 mM). Induced cultures were incubated overnight (~18 h) at 30 °C, 200 rpm. Cells were harvested by centrifugation (8,000 x g 10 min, 4 °C) then resuspended and washed with PBS buffer (40 mL per 1 L culture). Cells were pelleted again (8,000 x g, 30 min, 4 °C), supernatant discarded and the pelleted cells were frozen and stored at -20 °C.

Purification

Cell pellets were defrosted and resuspended in lysis buffer (30 mL, 50 mM Tris-HCl, 20 mM imidazole, 150 mM NaCl, 0.5 mM benzamidine, pH 8). Lysozyme (20 mg), DNase I (1 mg), and

$MgCl_2$ (1 mL, 1 M) were added to the lysate and incubated at 4 °C for 1 h. Cell lysates were sonicated (2 min, 90 % power with 10 s pulses) then clarified by centrifugation (12,000 x rpm, 1 h, 4 °C). Supernatant was filtered (0.45 µm filter Millipore, Merck), and protein was purified by IMAC: A 5 mL HisTrap FF column was equilibrated with 5 CV of wash buffer (30 mM Tris-HCl, 150 mM NaCl, 20 mM imidazole, pH 8), filtered lysate was applied at a constant flow-rate of 5 mL/min. The column was then washed with 5-10 cv of wash buffer before His-tagged proteins were eluted with 6 cv of 60 % elution buffer (30 mM Tris-HCl, 150 mM NaCl, 330 mM imidazole, pH 8). Eluted proteins were dialyzed against 5 L of dialysis buffer (30 mM Tris-HCl, 150 mM NaCl, 10 mM imidazole, pH 8) at 4 °C overnight. DTT (1 mM) and EDTA (0.5 mM) were added to the protein and the His-tag was cleaved by the addition of TEV-protease (0.014 equivalents) and incubation at room temperature for 6 h. After the TEV cleavage, the protein solution was subjected to 5 mL HisTrap FF column equilibrated with wash buffer at a constant flow of 5 mL/min. The flow-through that contained purified TT_SCP variants with His-tag cleaved was collected and analysed by SDS PAGE (NuPAGE 4-12 % Bis-Tris Gel; NuPAGE MES SDS Running Buffer, Life Technologies). The protein concentrations were determined using A280 and the extinction coefficients corrected for each mutant.

Gels

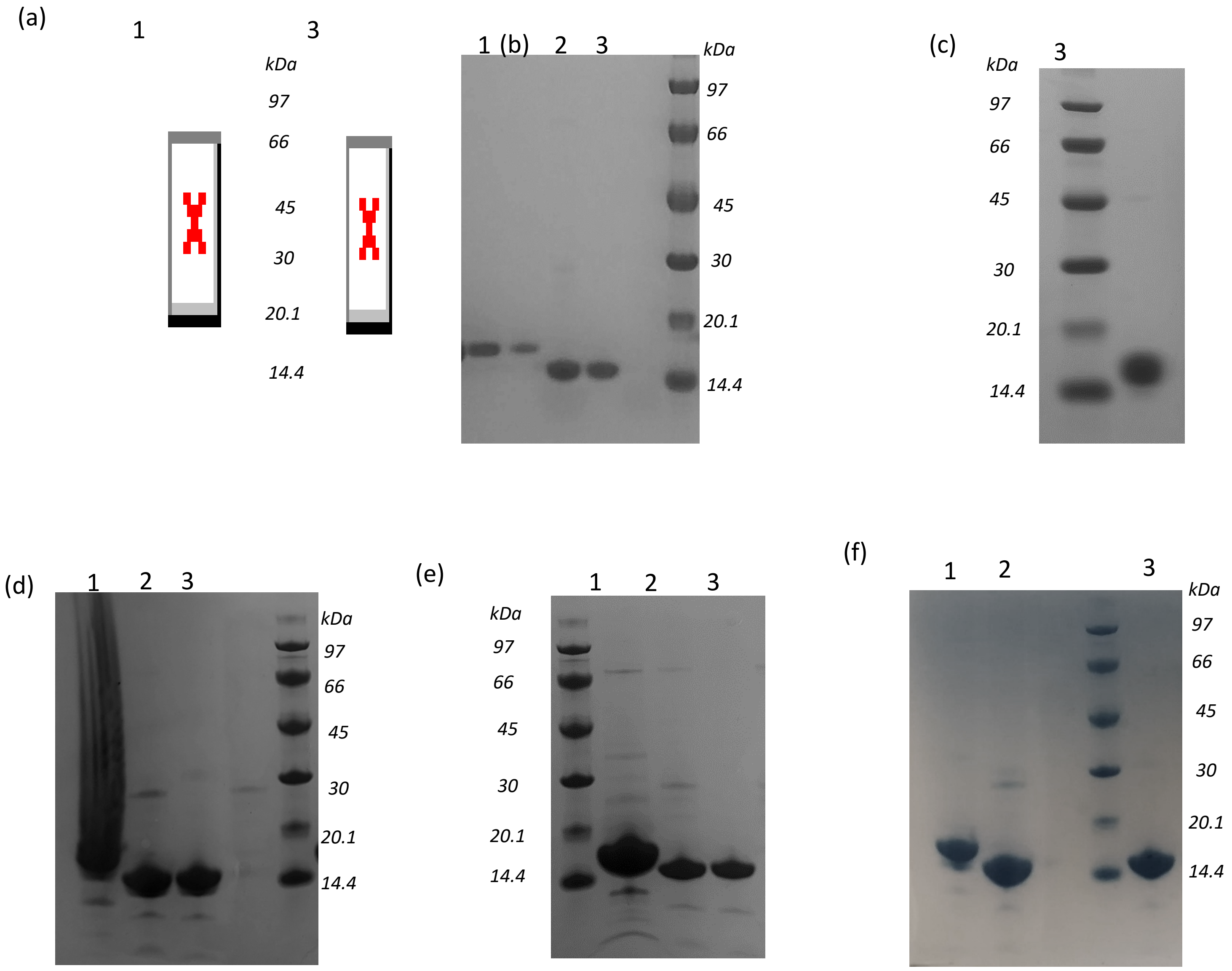

Figure S6*: Gels of purified SCPs with HisTag protein lanes labelled 1, TEV protease cleaved proteins lanes labelled 2 and the final purified cleaved protein labelled 3.*

(a) hSCPA100C (b) TTSCP (c) TTSCP W83C (d) TTSCP L102C (e) TTSCP ΔDSB L02C (f) TTSCP L112C

Protein functionalisation

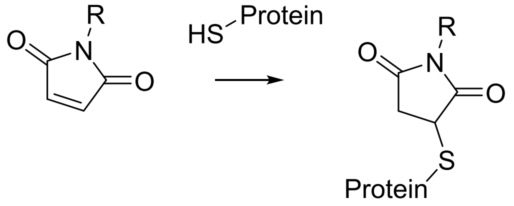

Figure S7: *General scheme for functionalisation of a cysteine-containing protein with a maleimide.*

Proteins (100 µM) were functionalised by incubation with MBnThz **1** (conditions specified for each construct Table S1). Reactions were centrifuged to remove any ppt and excess MBnThz was removed via buffer exchanging using a pd10 column equilibrated with reaction buffer (50 mM HEPES, pH8). Concentration of functionalised protein was determined via Bradford assay and when required protein was concentrated using Vivaspin 6 centrifugal concentrator, MWCO 5 kDa (Sartorius).

Table S1 *Reaction conditions used for functionalisation of each SCP mutant.*

Protein	Functionalisation conditions
hSCP A100C	20 mM Mes pH 6, 10 equiv MBnThz, 2 h, RT
TTSCP V83C	20 mM Mes pH 6, 10 equiv MBnThz, 5 h, RT
TTSCP L102C	20 mM Mes pH 7, 5 equiv MBnThz, 1.5 h, RT
TTSCP L112C	20 mM Mes pH 7, 10 equiv MBnThz, 0.5 h, RT
TTSCP ΔDSB L102C	20 mM Mes pH 7, 5 equiv MBnThz, 1.5 h, RT

Table S2 *A summary of the yield, expected mass and observed mass for each protein both before and after functionalisation with* ***(1)***

	Yield mg/L	Expected Mass (Da)	Observed Mass (Da)	Expected Mass Functionalised with 1 (Da)	Observed mass of functionalised product
hSCP A100C	11	13404.66	13404.74	13717.66	13717.1 (98% monofunctionalisation)<br>14047.5 (2% difuncitonalisation +ring opening)
TTSCP	54	14865.04	14863.0 (accounts for DSB)	-	-
TTSCP W83C	27	14781.9	14780.3 (accounts for DSB)<br>15046.2 (Stearoylation)	15094.90	14779.7 (80% unfunctionalised)<br>15045.8 (Stearoylation unfunctionalised)<br>15091.0 (~5% potential mono-functionalisation)<br>15719.0 (~10% trifunctionalised)
TTSCP L102C	49	14855.02	14853.6 (accounts for DSB)	15168.02	14854.3 (40% unfunctionalised)<br>15166.6 (50% mono-functionalisation accounts for DSB still present)<br>15480.9 (5% di-functionalised)<br>15793.2 (5% tri-functionalised)
TTSCP L112C	55	14855.02	14853.4 (accounts for DSB)	15168.02	15165.3 (85% mono-functionalisation - accounts for DSB still present,)<br>15479.5 (10% di-functionalised)<br>15792.2 (5% tri-functionalised)
TTSCP ΔDSB L102C	24	14790.90	14790.9	15103.90	14792.2 (25% unfunctionalised)<br>15104.7 (75% mono-functionalisation)

Protein LC-MS

hSCP A100C

Expected mass: 13404.66   Mass Found: 13404.74

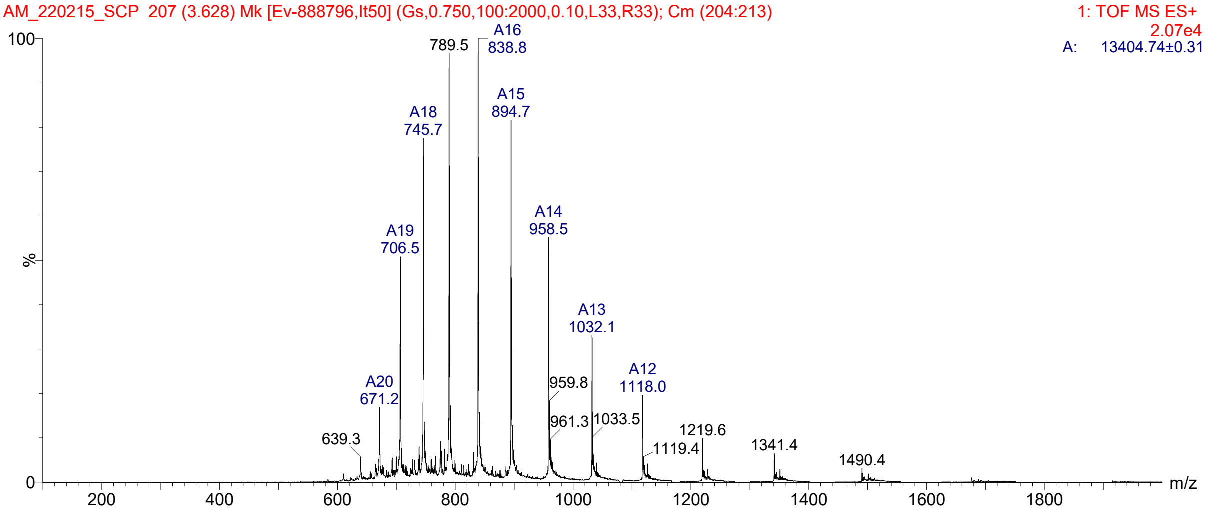

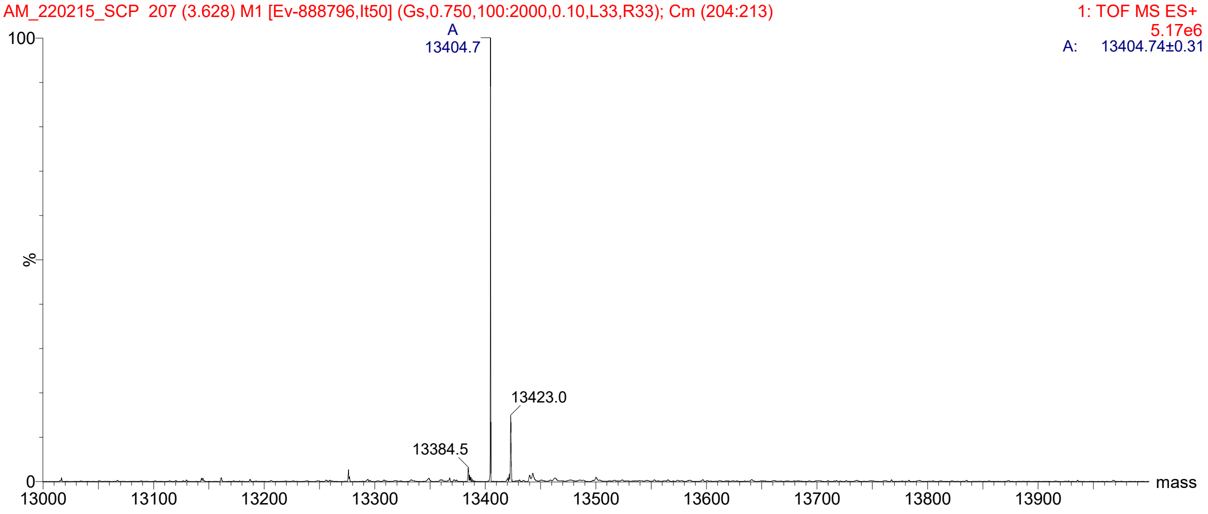

Figure S8: LC-MS of hSCP A100C

TTSCP

Expected mass: 14865.04   Mass Found 14863.0 (accounts for disulfide bond formation)

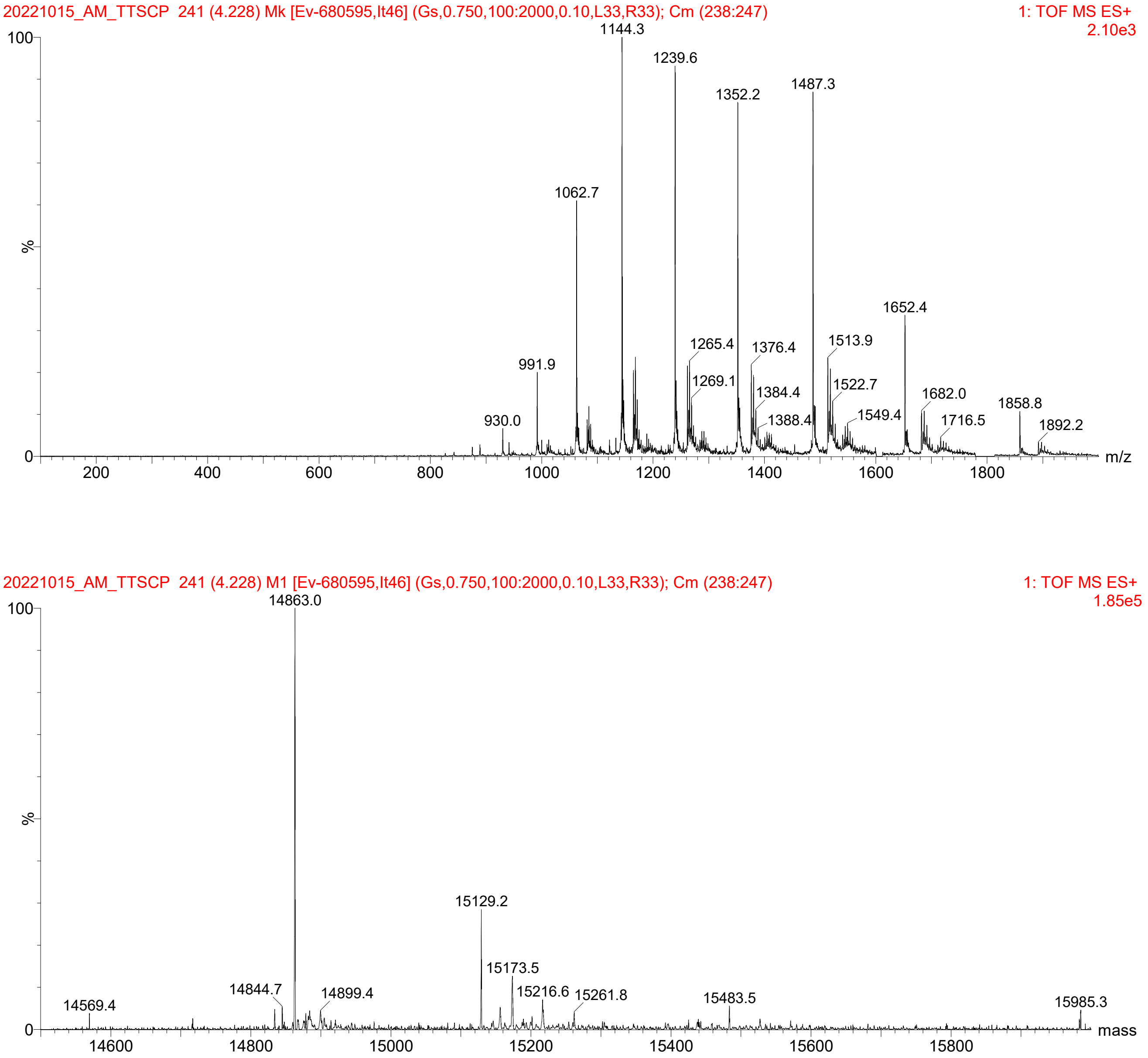

Figure S9: LC-MS of TTSCP

TTSCP W83C

Expected mass: 14781.9   Mass Found 14780.3 (accounts for disulfide bond formation)

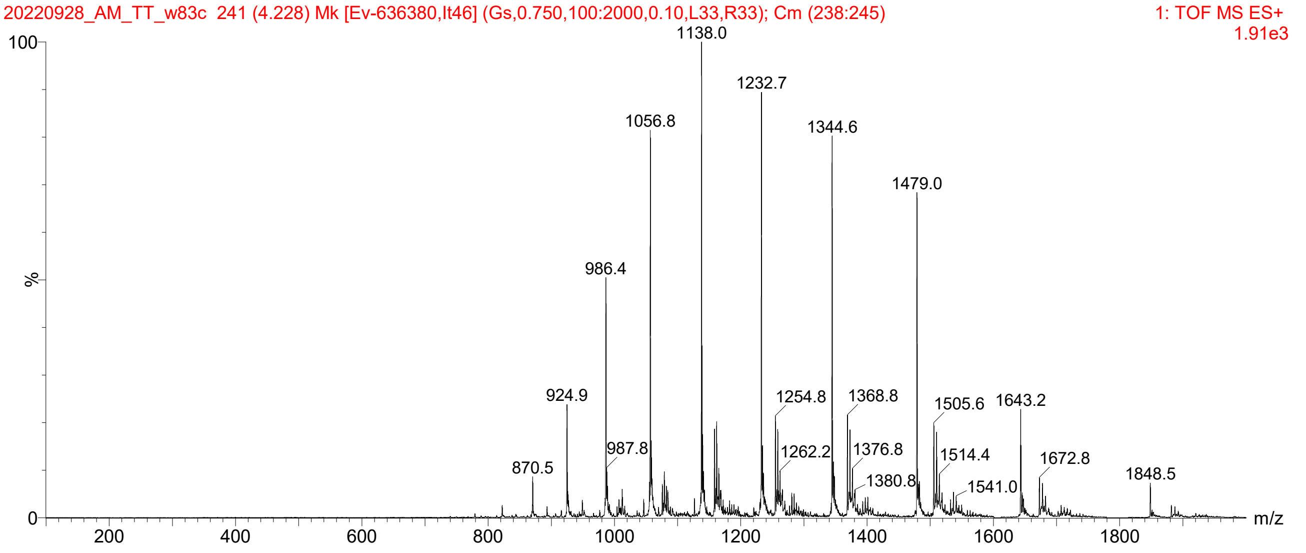

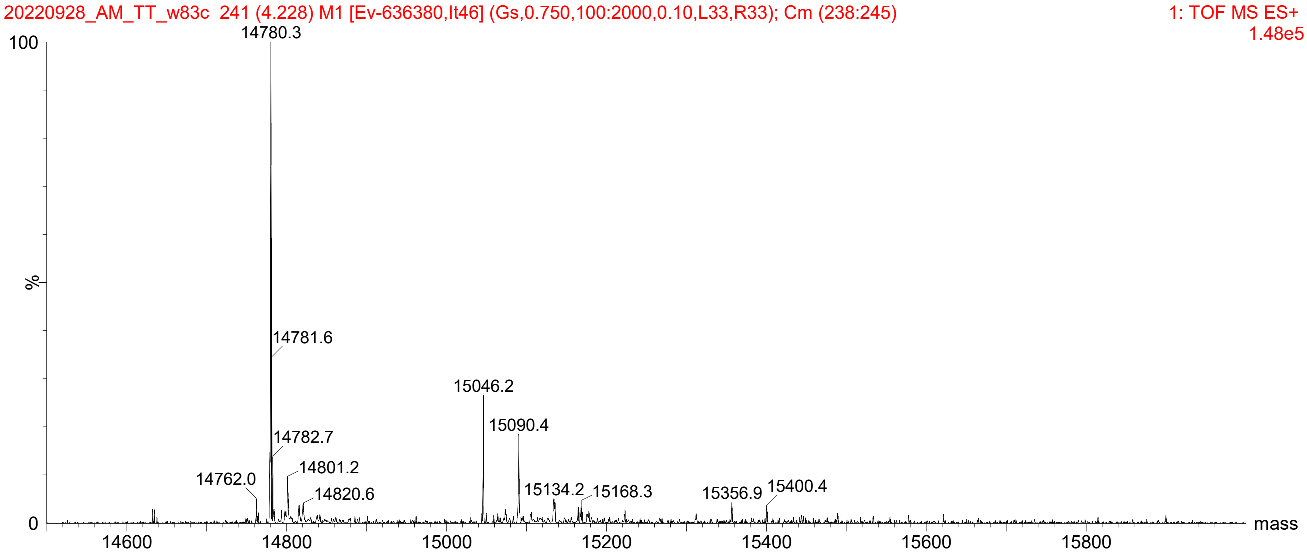

Figure S10 : LC-MS of TTSCP W83C

TTSCP L102C

Expected mass: 14855.02 Mass Found 14853.6 (accounts for disulfide bond formation)

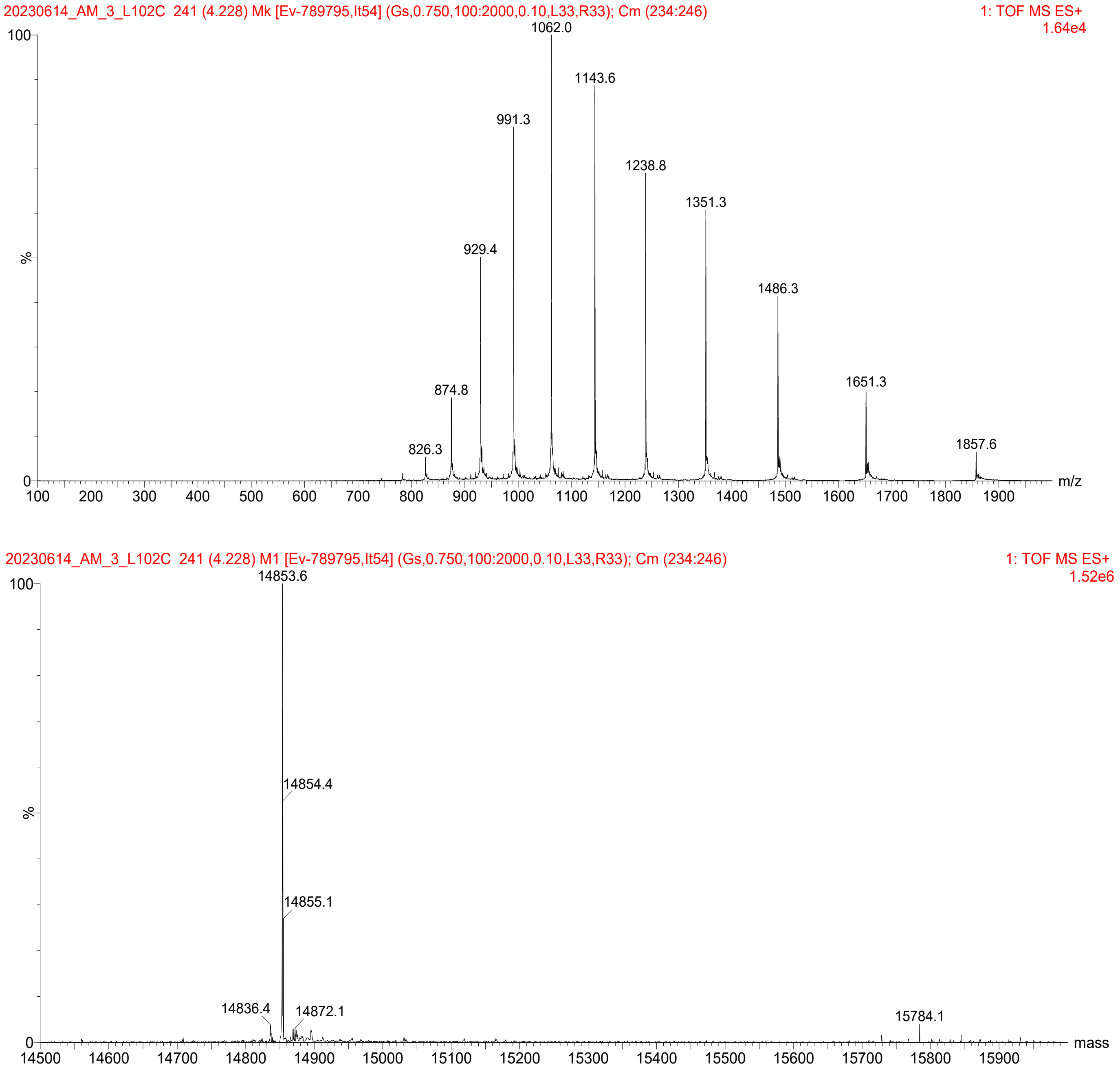

Figure S11: LC-MS of TTSCP L102C

TTSCP L112C

Expected mass: 14855.02   Mass Found 14853.4 (accounts for disulfide bond formation)

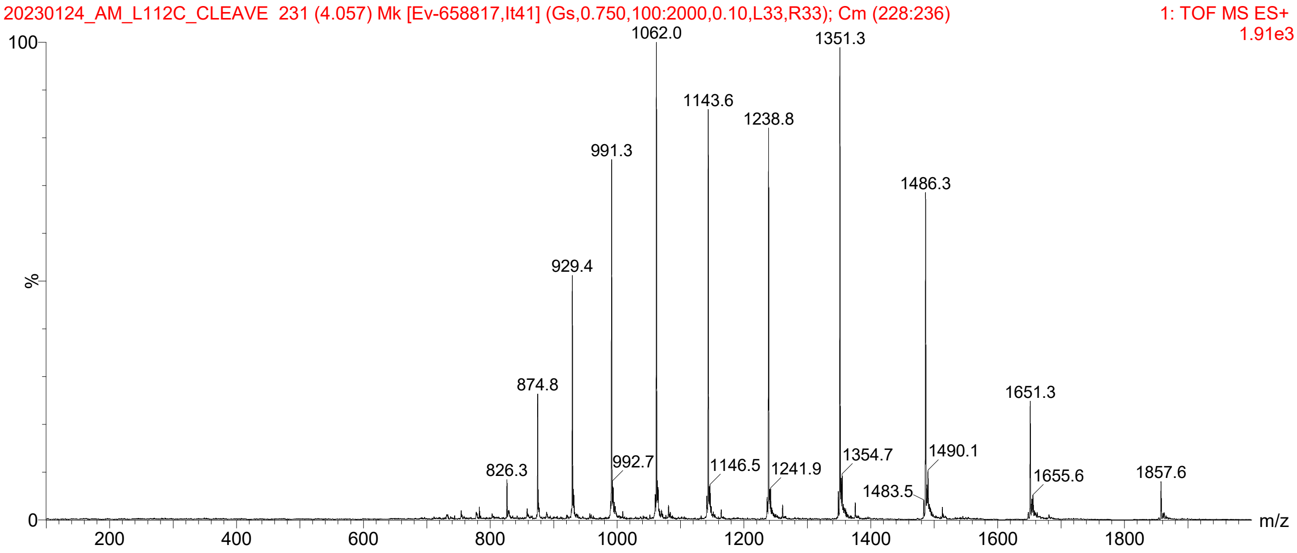

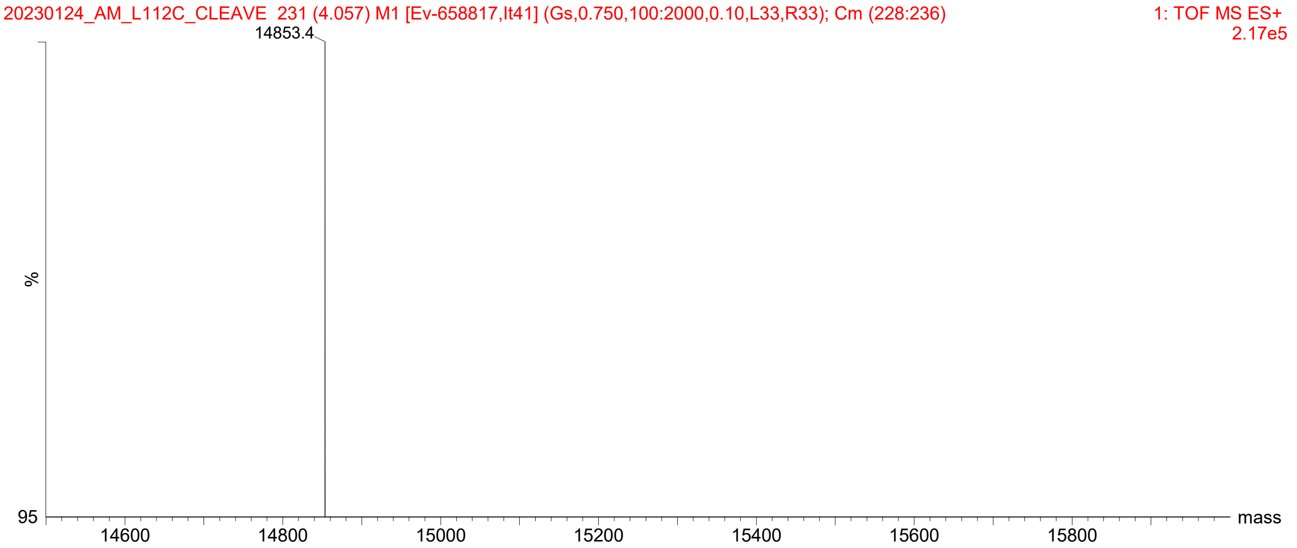

Figure S12 LC-MS of TTSCP L112C

TTSCP ΔDSB L102C

Expected mass: 14790.90 Mass Found 14790.9

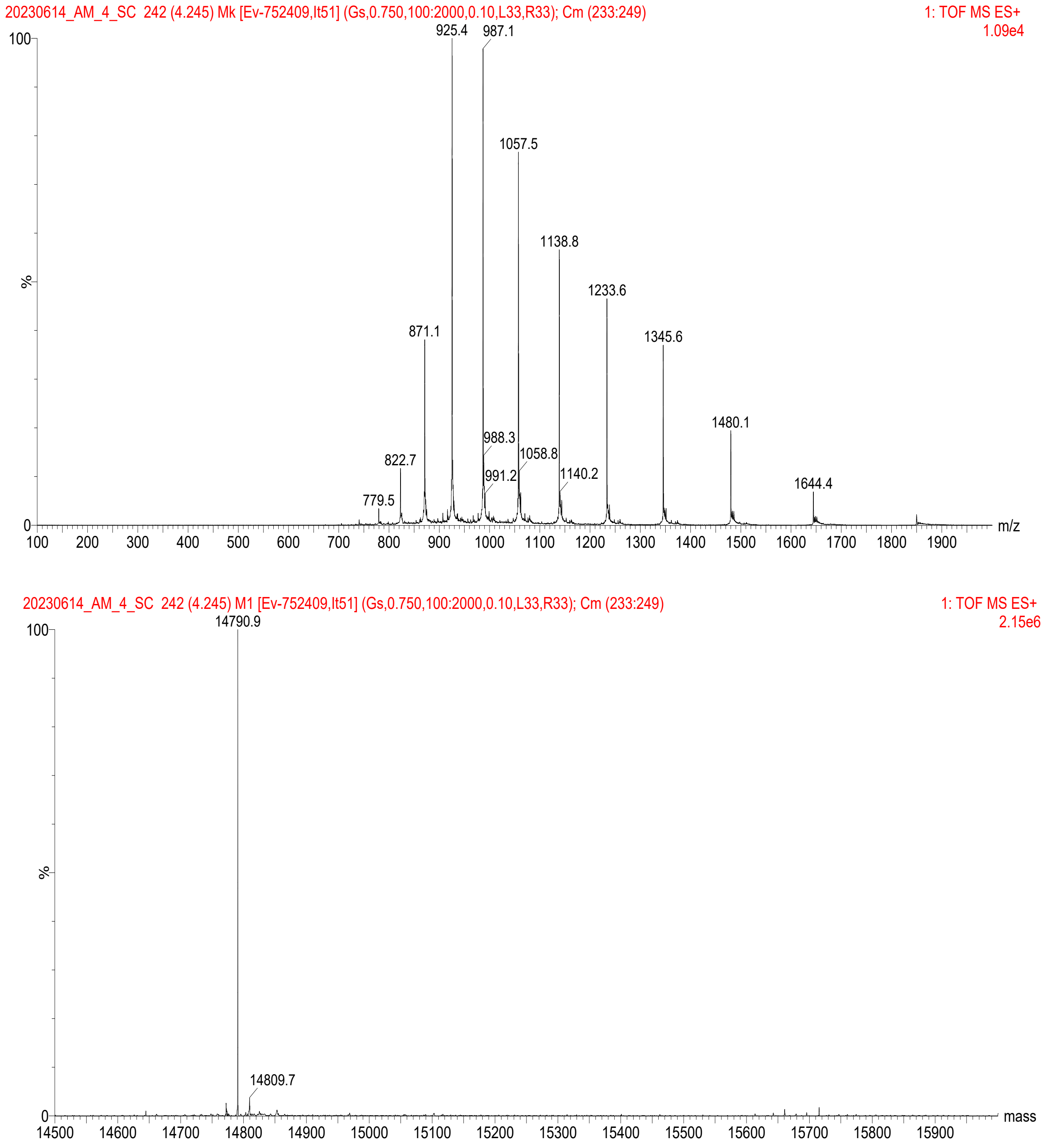

Figure S13: LC-MS of TTSCP ΔDSB L102C

hSCP A100C MBnThz

Expected mass: 13717.66   Mass Found 13717.1 Mono-functionalisation 100%

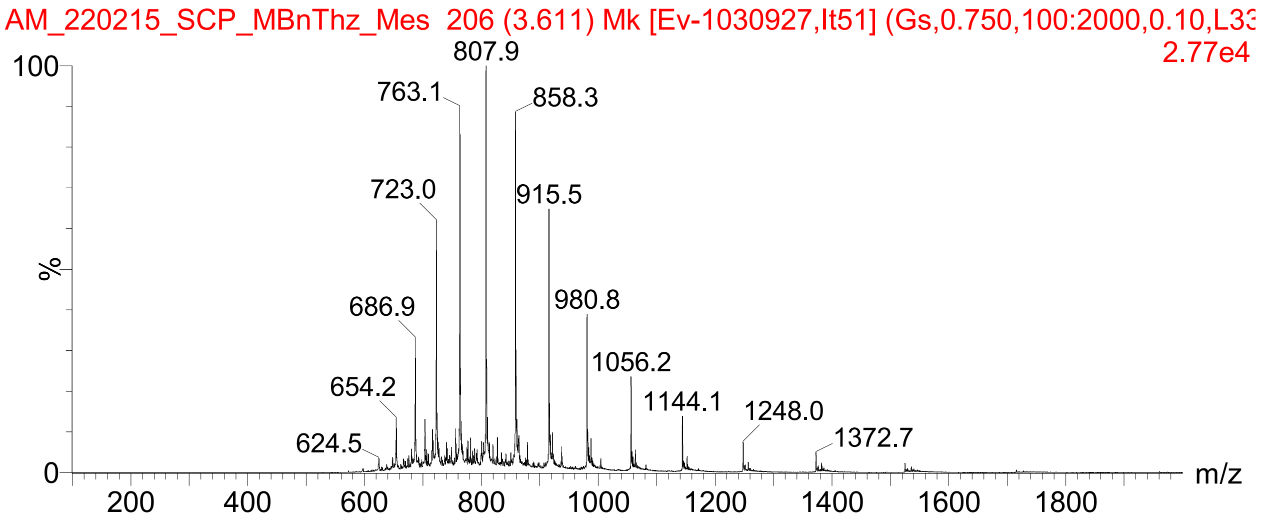

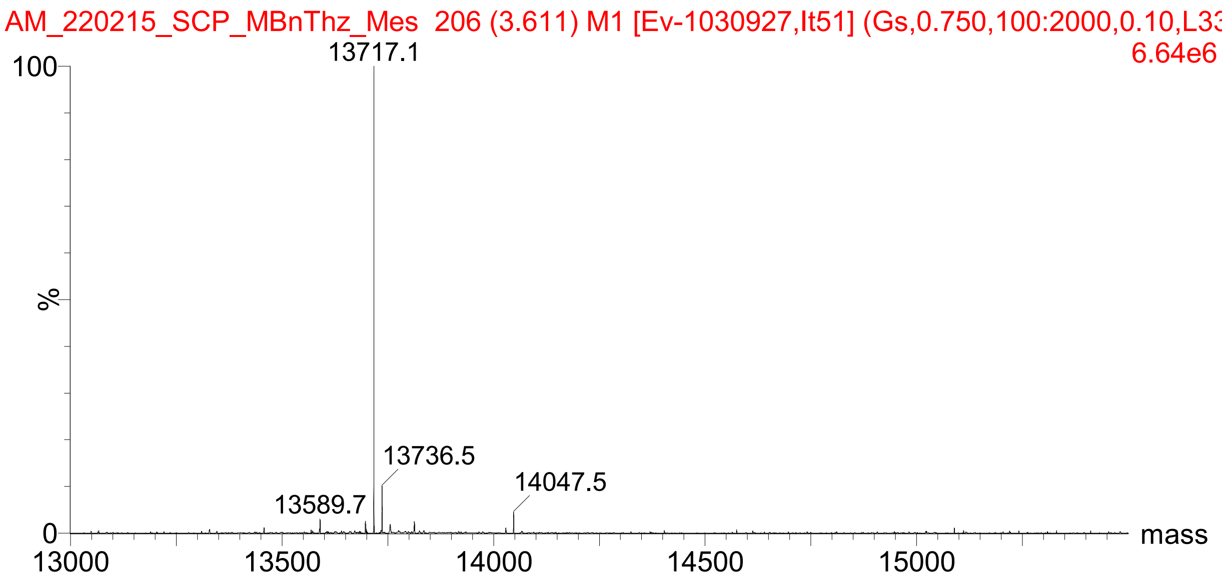

Figure S14 LC-MS of hSCP A100C functionalised with **(1)**

TTSCP W83C MBnThz

10 equiv 5 h RT 20 mM Mes pH6

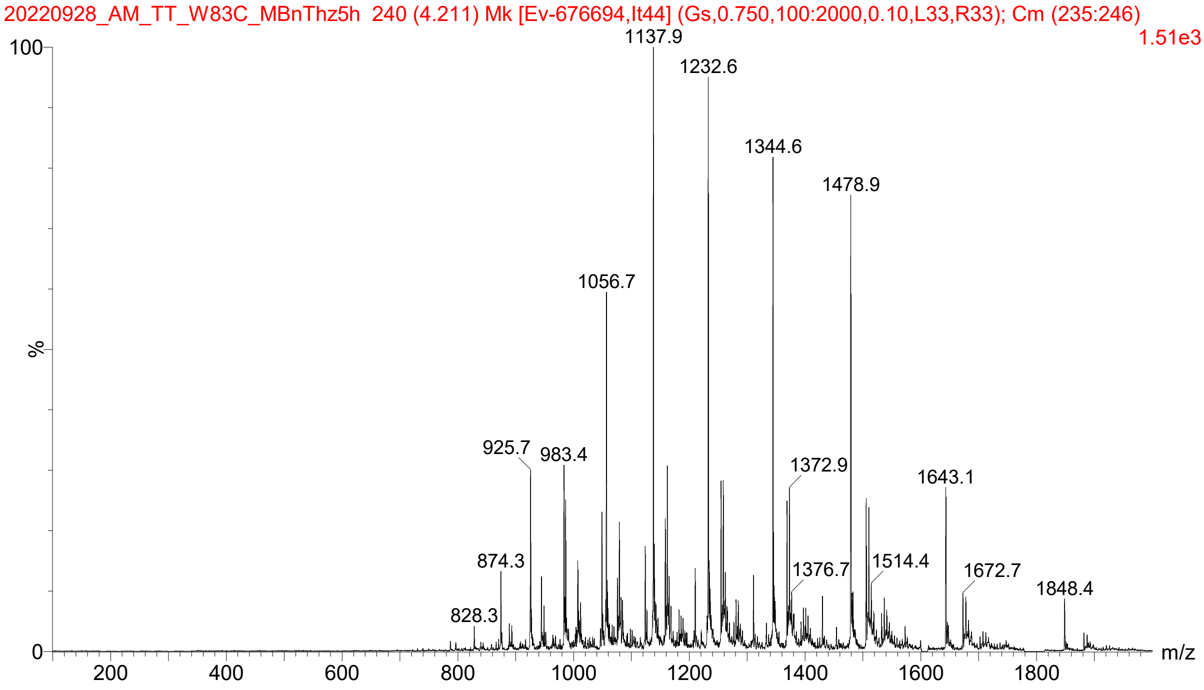

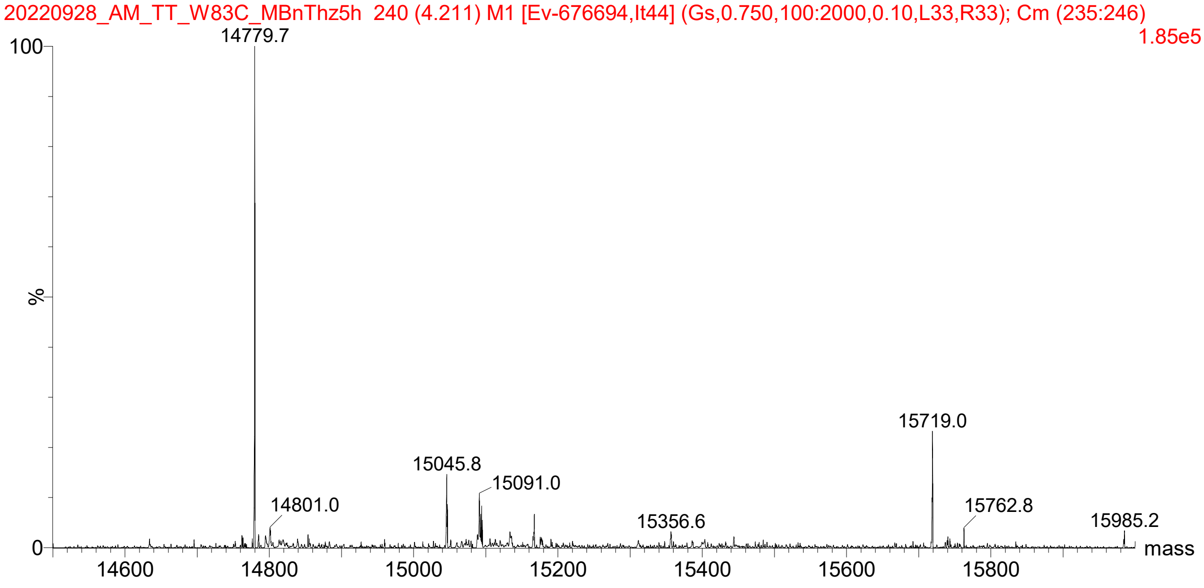

Figure S15 LC-MS of TTSCP W83C functionalised with **(1)**

TTSCP L102C MBnThz

Expected mass 15168.02 Mass Found 15166.6 (accounts for disulfide bond still present)

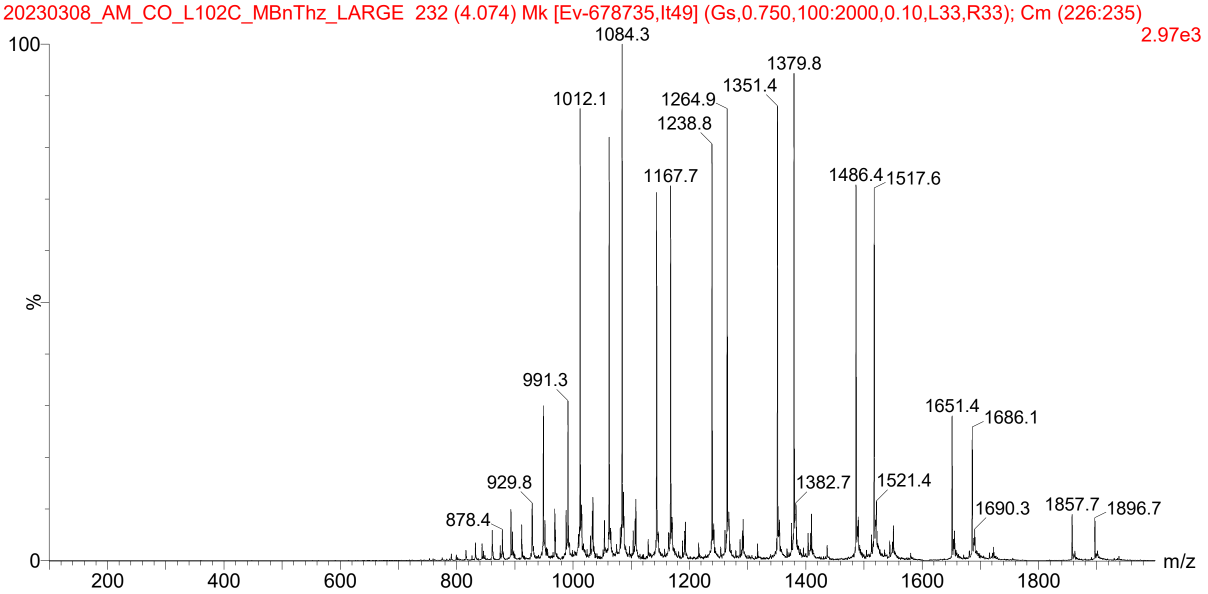

Mono-functionalisation Percentage: 50%

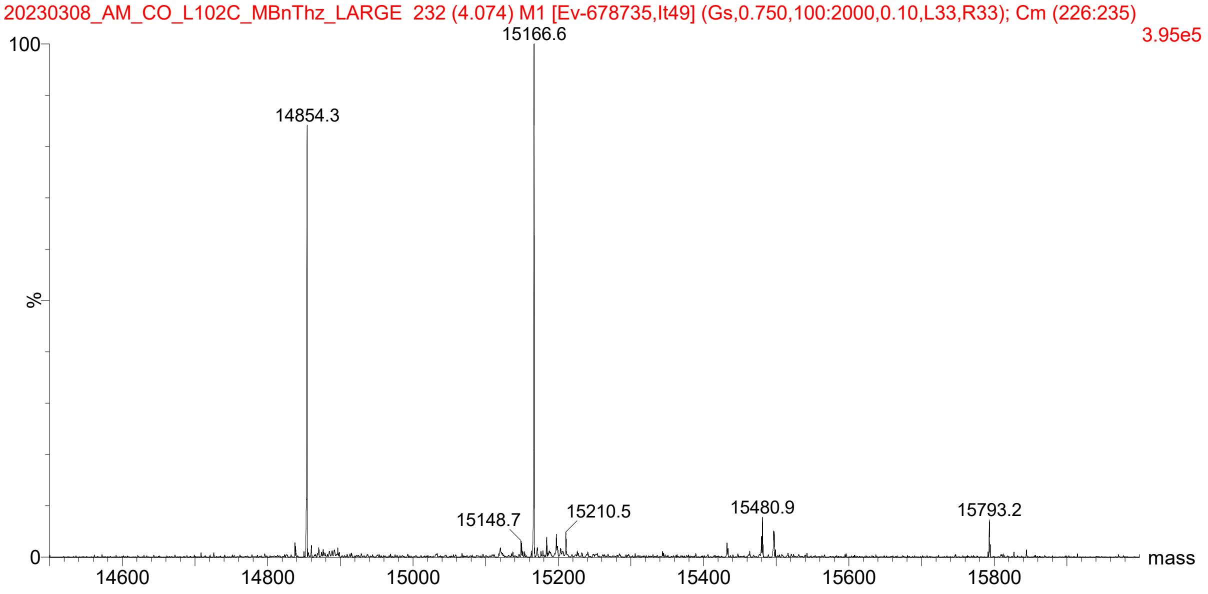

Figure S16: LC-MS of TTSCP L102C functionalised with **(1)**.

TTSCP L112C MBnThz

Expected mass 15168.02 Mass Found 15165.3 (accounts for disulfide bond still present)

Mono-functionalisation Percentage: 85%

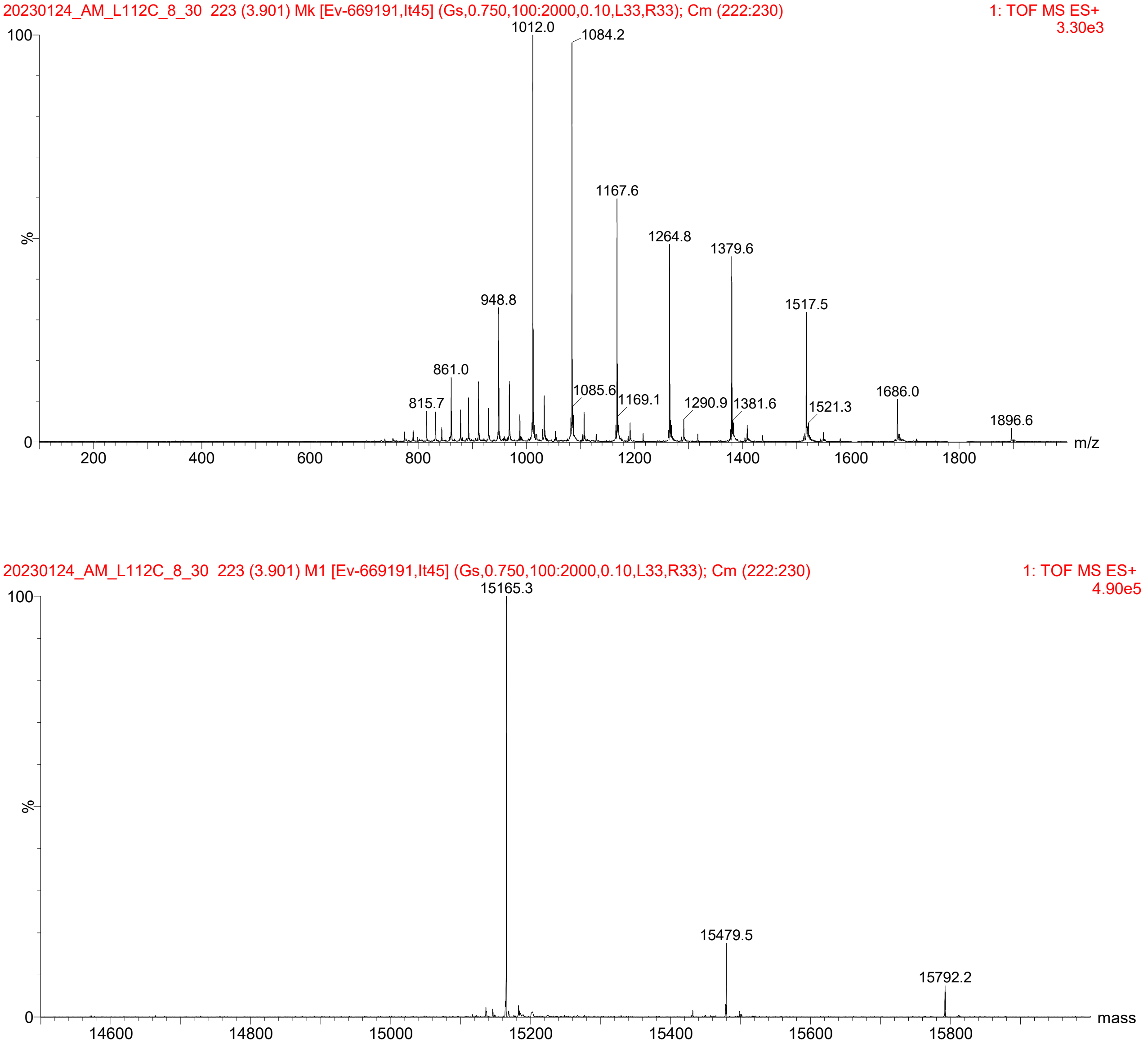

Figure S17: LC-MS of TTSCP L112C functionalised with **(1)**

TTSCP ΔDSB L102C MBnThz

Expected mass 15103.90   Mass Found 15104.7 Mono-functionalisation Percentage: 75%

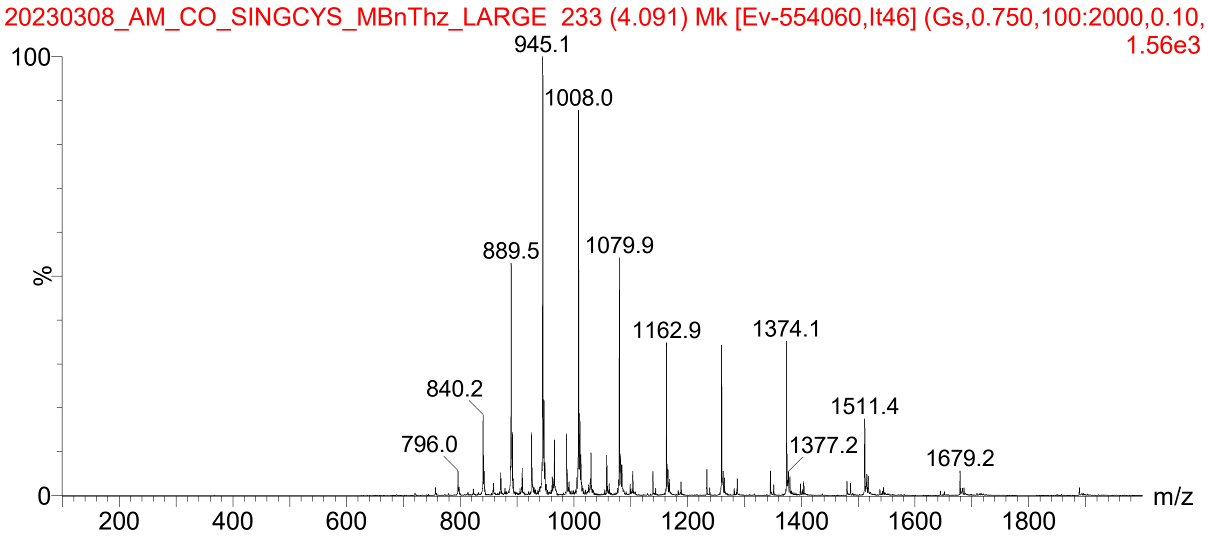

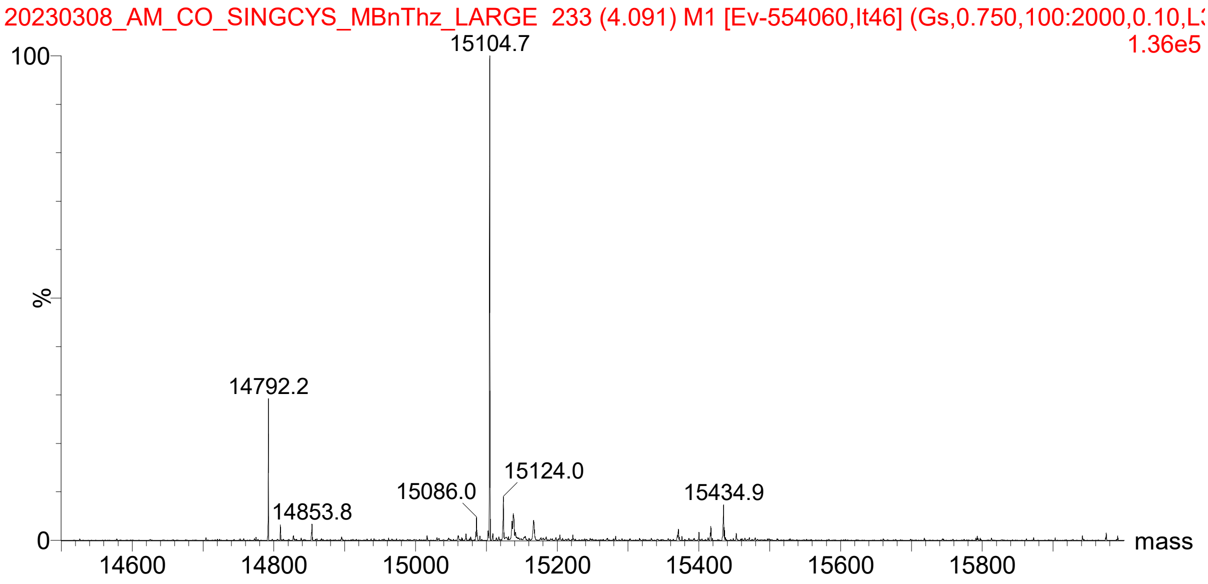

Figure S18: LC-MS of TTSCP ΔDSB L102C functionalised with **(1)**

Intramolecular Stetter reaction

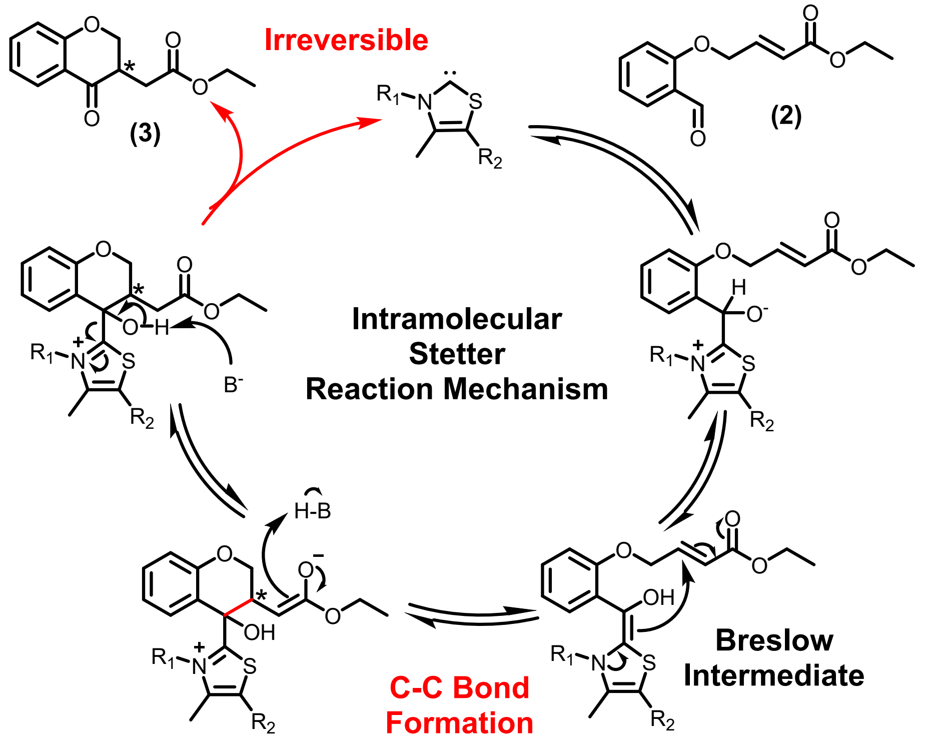

Figure S19: *An overview of the intramolecular Stetter reaction mechanism highlighting the Breslow intermediate and key steps including the formation of the C-C bond and the irreversible conversion of substrate* ***(2)*** *to product* ***(3)****.*

Synthesis of Stetterase substrate

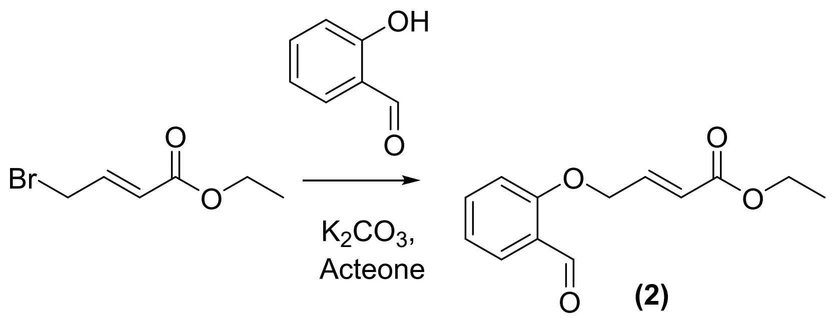

(2)

Figure S20: *Synthesis of Intramolecular Stetter starting material* ***2****, (4-(2-Formyl-phenoxy)-but-2-enoic acid ethyl ester):* Salicylic aldehyde (1.7 mL, 16 mmol, 1 equiv) and K_2CO_3 (3.3 g, 24 mmol, 1.5 equiv) were dissolved in 30 mL of acetone, and ethyl 4-bromocrotonate (2.35 mL, 17 mmol, 1.05 equiv) was added into the mixture then stirred overnight. The reaction was quenched with water (10 mL) and the organic layer was extracted with ethyl acetate (30 mL X 3). The organic layer was dried over $MgSO_4$ and concentrated *in vacuo*. The product was purified by flash chromatography (hexane/ethyl acetate) as a precipitated as a yellow solid.

^{1}H NMR (500 MHz, $CDCl_3$) δ 10.57 (s, 1H), 7.88 (dd, *J* = 7.7, 1.8 Hz, 1H), 7.56 (ddd, *J* = 8.5, 7.3, 1.9 Hz, 1H), 7.15 – 7.07 (m, 2H), 6.95 (dd, *J* = 8.4, 0.9 Hz, 1H), 6.23 (dt, *J* = 15.8, 2.1 Hz, 1H), 4.84 (dd, *J* = 4.1, 2.1 Hz, 2H), 4.24 (q, *J* = 7.1 Hz, 2H), 1.32 (t, *J* = 7.1 Hz, 3H).

^{13}C NMR (126 MHz, $CDCl_3$) δ 189.28, 165.81, 160.20, 141.14, 135.91, 128.86, 125.17, 122.58, 121.46, 112.55, 66.89, 60.74, 14.22.

Exact mass calcd for $[C_{13}H_{14}O_4Na]^+$ ESI $[M+]^+$ requires *m/z* =257.0789 ,found *m/z* = 257.0786.

The NMR and MS data for compound 2 are in accordance with literature values[2].

Synthesis of Stetterase product

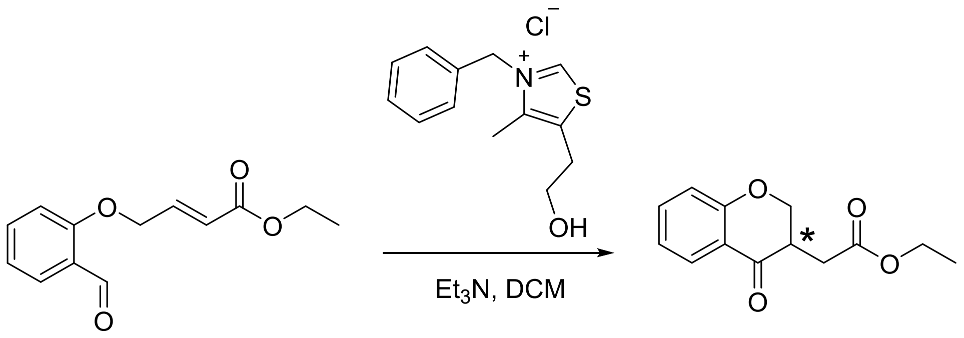

(2) (3)

Figure S21 To obtain product **3** for calibration compound **2** (2.5 mmol, 585 mg, 1 equiv) and 3-benzyl-5-(2-hydroxyethyl)-4-methylthiazolium chloride (0.5 mmol, 134.5 mg, 0.2 equiv) were dissolved in DCM (30 mL). Triethylamine (0.5 mmol, 70 µL, 0.2 equiv) was added and the reaction was stirred at 50°C for 16 hours. Reaction was cooled and solvent was removed under reduced pressure. Product was purified via flash column chromatography (5:1 hexane ethylacetate).

^{1}H NMR (500 MHz, $CDCl_3$) δ 7.90 (dd, *J* = 7.9, 1.8 Hz, 1H), 7.51 – 7.47 (m, 1H), 7.05 – 6.96 (m, 2H), 4.61 (dd, *J* = 11.2, 5.3 Hz, 1H), 4.31 (t, *J* = 11.9, 11.3 Hz, 1H), 4.24 – 4.17 (m, 2H), 3.34 (ddt, *J* = 12.1, 8.2, 5.1 Hz, 1H), 2.94 (dd, *J* = 16.9, 4.8 Hz, 1H), 2.43 (dd, *J* = 16.9, 8.1 Hz, 1H), 1.30 (t, *J* = 7.1 Hz, 3H).

^{13}C NMR (126 MHz, $CDCl_3$) δ 192.61, 171.36, 161.75, 136.01, 127.40, 121.53, 120.52, 117.84, 70.26, 60.98, 42.54, 30.38, 14.18.

Exact mass calcd for [C13H14O4Na]+ requires m/z =257.0789 ,found m/z = 257.0784 (ESI).The NMR and MS data for compound 3 are in accordance with literature values[2].

Intramolecular Stetter reaction HPLC analysis.

HPLC method: Isocratic elution at 45% MeCN 0.1% TFA over 16 min, 30 °C

Standards were made up in 10% DMSO then diluted with an equal volume of MeCN (as reaction samples would be) and used to make calibration curve. Product elution time 12.02

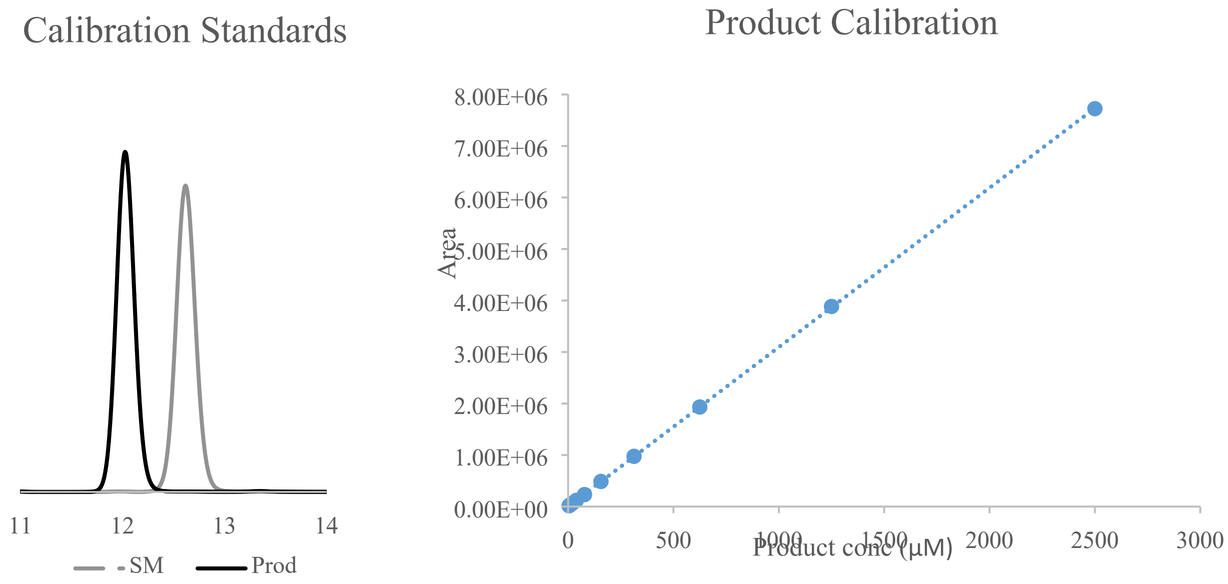

min , Starting material elution time 12.61 min

Figure S22: *Calibration standards of **2** (intramolecular Stetter starting material, SM grey dashed line) and **3** (intramolecular Stetter product, black line) and the calibration curve plotted for **3**.*

Intramolecular Stetter reactions using functionalised protein

Protein was buffer exchanged into reaction buffer (50 mM HEPES pH8) using a pd10 column. ISSM (10 µL, 25 mM in DMSO) and functionalised protein (final concentration 100 µM) were added to a 1.5 mL Eppendorf tube. Reaction buffer was added to a final volume of 100 µL. Each reaction was set up in triplicate. Reactions were incubated in a thermoshaker (Grant Instruments™ PCMT) at 30 °C, 250 rpm for 18 hours. Reactions were quenched with 100 µL MeCN containing 0.1% TFA and 1 mM Coumarin as an internal standard. Reactions were centrifuged to remove precipitated protein and supernatant was analysed by HPLC.

A100C samples were run without a column oven therefor shifted retention time was observed but control samples confirmed retention times of **2** and **3** as 12.41 and 13.09 respectively**.**

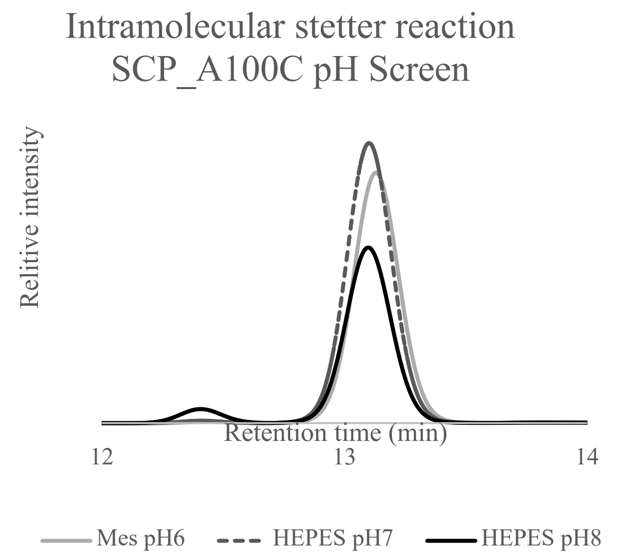

Figure S23*: HPLC traces of the intramolecular Stetter reaction carried out with hSCPA100C_MBnThz under a range of pHs.(20 mM Mes pH6, grey, 50mM HEPES pH7, grey dashed, 50 mM HEPES pH8 black) Product is observed only in the reaction performed at pH 8.*

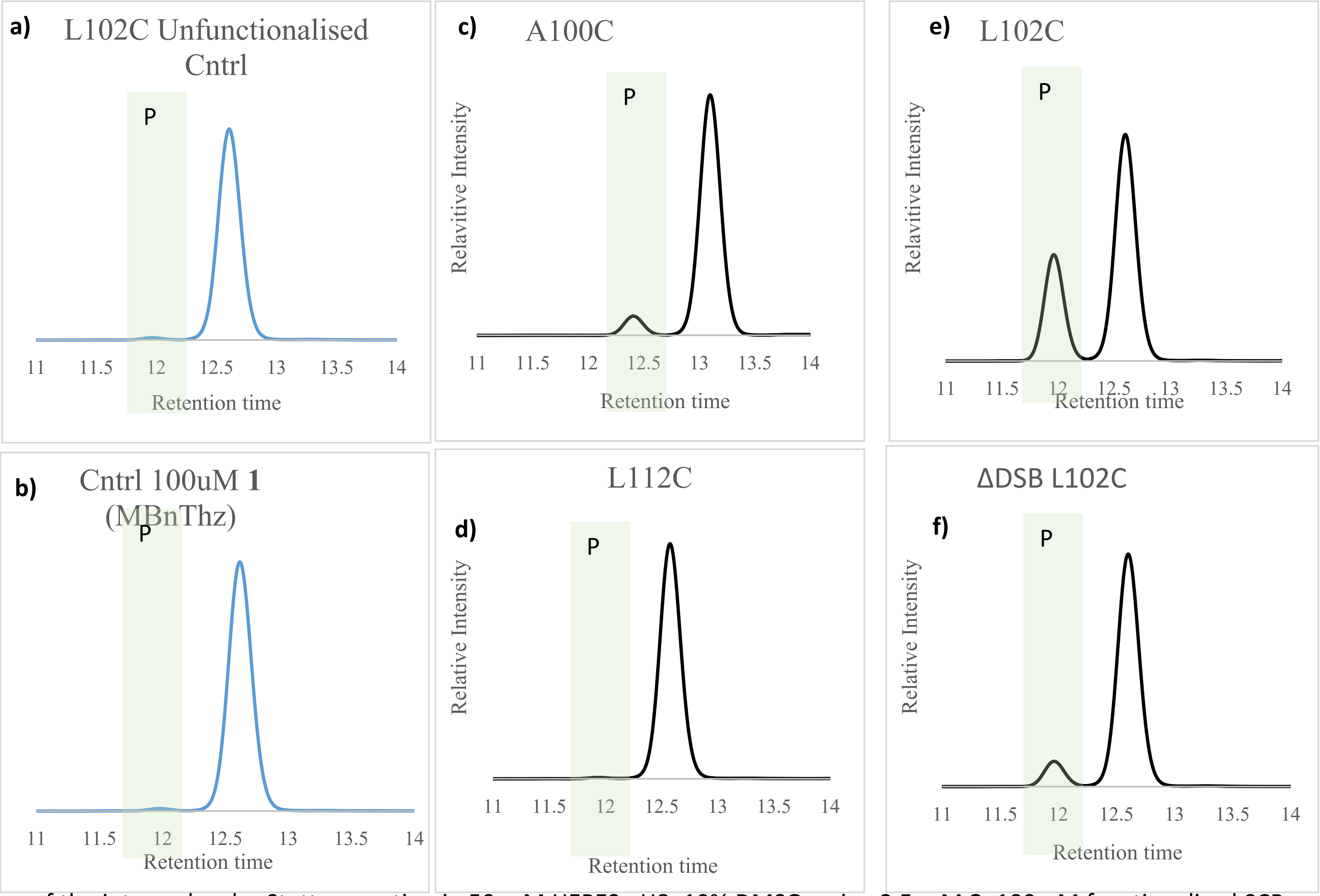

Figure S24: HPLC traces of the intramolecular Stetter reaction in 50 mM HEPES pH8, 10% DMSO, using 2.5 mM **2**, 100 µM functionalised SCPs as catalyst, 30 °C, 250 rpm for 18 hours. **a)** Control reaction with 100 µM unfunctionalised TTSCP A100C, **b)** Control reaction with 100 µM **1** **c)** 100 µM hSCP A100C **d)** 100 µM TTSCP L112C **e)** 50 µM TTSCP L102C **f)** 100 µM TTSCP ΔDSB L102C. Peak corresponding to the formation of product **3** is highlighted green.

Enantioselecitivty

To analyse the enantio selectivity of the reaction, the samples were run on CHIRALPAK® IB N-5 analytical column (5 µm, 250 × 4.6 mm) using an isocratic method for 30 mins, 40% MeCN, 60% 50 mM Kphos pH 2.0

To determine which enantiomer we had formed, we expressed recombinant pfBAL. Performing reacions with pfBAL under similar conditions (50 Mm Kphos pH 7, 20% DMSO, 150 µL pfBAL lysate, 2.5 mM $MgSO_4$, 0.15 mM TPP, 2.5 mM **2**, 24 hours, 30 °C, 250 rpm) to those reported by Chen *et al.*, we obtained an e.r 74: 26 similar to their value with 20% DMSO 71:29[2]. Comparison with PfBAL suggests we have a slight excess of the *R* enantiomer (5%

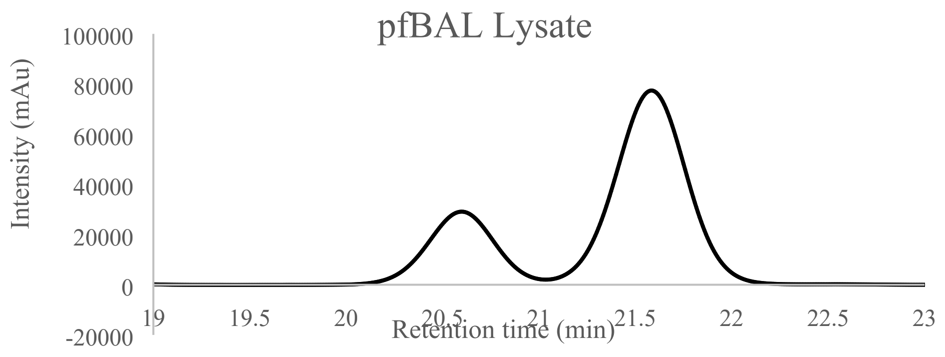

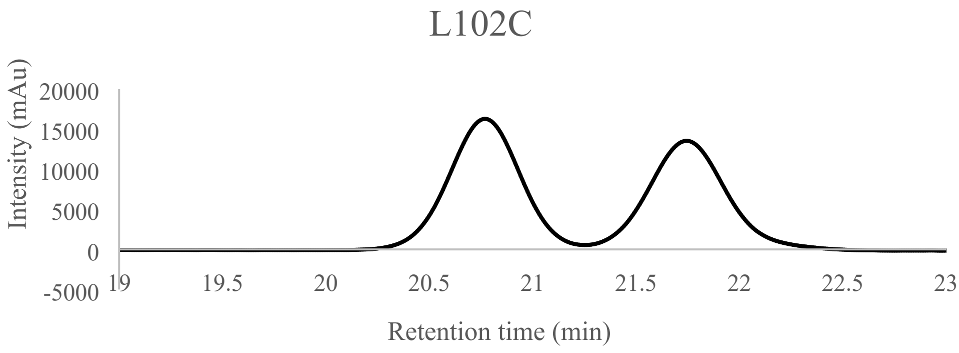

e.e.)

	Peak area (R)	*Peak area (S)*	*ee*	*e.r*
L102C (b)	926207	819757	(R) 6.10	53:47
L102C (b)	946210	843340	(R) 5.75	53:47
L102C (c)	344597	310094	(R) 5.27	53:47
L102C (d)	405255	368290	(R) 4.78	52:48
PfBAL lysate	729538	2041912	(S) 47.35	26:74
PfBAL purified	600078	1344234	(S) 38.27	31:69

Figure S25: Chiral HPLC analysis of the intramolecular Stetter reaction performed with pfBAL lysate and TTSCP L102C functionalised with **1**. 20.77 (*R*–enantiomer), 21.74 (*S*-enantiomer).

Structural analysis

Where structures were not available from the PDB, structural predictions were created using ColabFold via AlphaFold2 using MMseqs2 and relaxed using amber. Docking was performed with AutoDock Vina (v1.1.2). Ligands and receptors were prepared in AutoDockTools. For docking a grid box size of 26x24x22 Å,with coordinates -10.578, 5.582, -9.827 was used. CastP was used to analyse the cavities in each structure (**Figure S26**).

Table S3 Analysis of cavity size of SCP structures using CastP. # Pose 1 of NMR structure used to cavity analysis. * denotes data derived from AlphaFold2 model.

Protein	PDB code	Cavity internal area (Å^2)	Cavity volume (Å^3)	Cavity opening (Å^2)
**Human SCP_2L**	1IKT	688.6	1,076.4	119.8
**Human SCP_2L A100C**	6Z1W	891.2	1,320.2	229.9
**TT_SCP (chain A)**	2CX7	432.6	468.8	22.4
**TT_SCP (chain B)**	2CX7	562.9	595.5	0.0
**TT_SCP (NMR)#**	1WFR	526.6	563.9	0.0
**TT_SCP W83C***	Model	412.7	448.9	0.0
**TT_SCP L102C***	Model	653.3	629.9	46.8
**TT_SCP ΔDSB L102C***	Model	645.0	672.4	69.3
**TT_SCP L112C***	Model	627.2	745.3	77.3

It was noted that after mutation of W83 to Cys, the residue is no longer highlighted by CastP as lining the cavity which may suggest why this position is hard to functionalise.

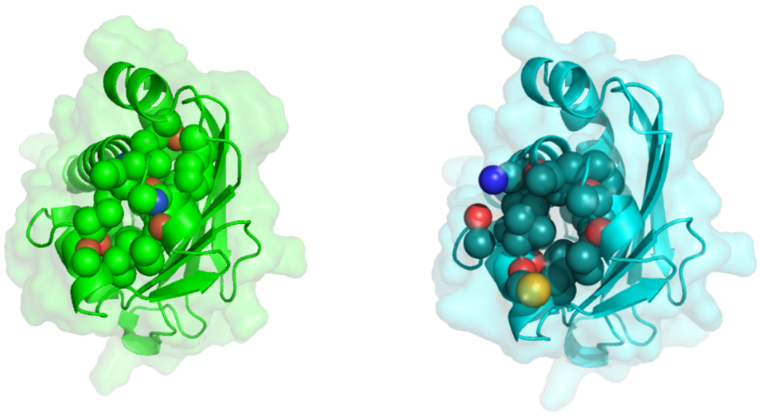

Figure S26: CastP showing atoms lining cavity of alpha fold models. Green TTSCP L102C. Teal TTSCP ΔDSB L102C.

Docking

Breslow Intermediate: We used AutoDock Vina to provide feasible poses of the Breslow intermediate (**8**) formed from **1** and **2** in the cavity of scaffold hSCP A100C (PDB: 6Z1W) and TTSCP (PDB: 2CX7 chain A). The calculated affinities are a guide to how the ligands bind since the Breslow intermediate forms in situ after the covalent attachment of MBnThz (**1**) onto a cysteine residue altering the exact position. The docking score for 6z1w gives a negative affinity indicating favourable binding, however none of the poses show the maleimide near A100C. Although slightly positive affinities were obtained indicates that the smaller cavity size could accommodate the Breslow intermediate.

a)

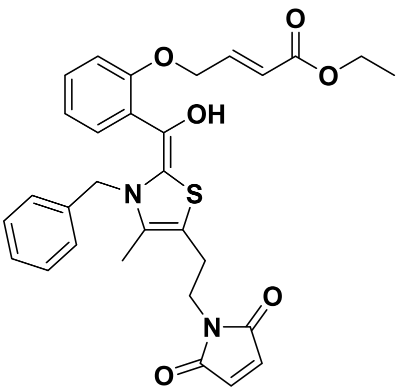

(8)

b)

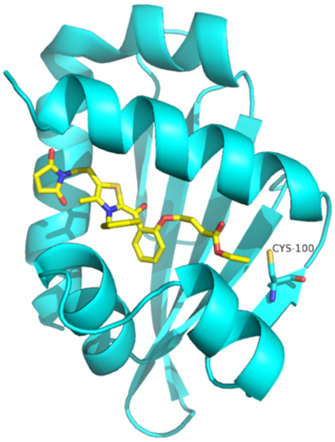

mode	affinity (kcal/mol)	dist from best mode rmsd l.b.	rmsd u.b.
1	-9.1	0.000	0.000
2	-8.7	1.434	2.154
3	-8.5	2.123	3.483
4	-8.0	2.109	3.324
5	-7.9	2.276	3.973
6	-7.8	2.307	3.956
7	-7.7	2.622	4.552
8	-7.6	3.167	9.551
9	-7.6	2.221	4.156

Writing output ... done.

c)

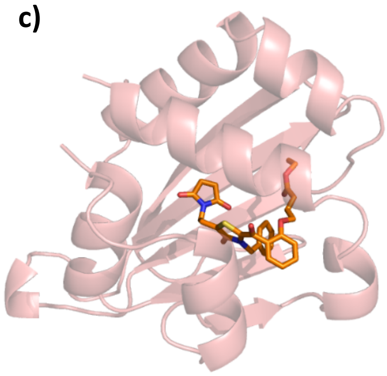

mode	affinity (kcal/mol)	dist from best mode rmsd l.b.	rmsd u.b.
1	5.6	0.000	0.000
2	5.8	0.130	1.421
3	6.8	2.277	6.702
4	6.8	3.051	8.679
5	8.0	2.395	8.427
6	8.6	2.431	7.519

Writing output ... done.

Figure S27: **a)** Structure of the predicted Breslow Intermediate **(8)** formed from **(1)** and **(2).** **b)** Pose of the docking of the intermediate into hSCP A100C (6Z1W). **c)** Pose of the docking of the intermediate into TTSCP (2CX7, chain A).

Maleimide Thiazole: MBnThz (**1**) was docked into TTSCP (2CX7, chain A) to identify residues as potential positions for cysteine mutations. Several poses gave similar positions but 1,2, and 8 showed three unique positions (**Table S4**). From these poses residues identified as being within 5 Å of the maleimide were L35, I76 W83, L94, L97 L102, L112, A116 and V123. Preference was given to residues which faced into the cavity (L35, I76, W83, L97, L102 and L112) over those that were solvent exposed (L94, A116 and V123) which may allow the maleimide thiazole to bind outside the cavity**.** Residues L35 and I76 were not chosen since the model suggested they formed part of the beta sheet which we did not want to disrupt. Residues W83, L102 and L112 were chosen to mutate to cysteine for initial studies.

Table S4*:* Three distinct poses generated by docking of **1** into TTSCP (2CX7 chain A)

For each pose, residues within 5 Å of the maleimide are highlighted.

**Pose 1**	**Pose 2**	**Pose 8**
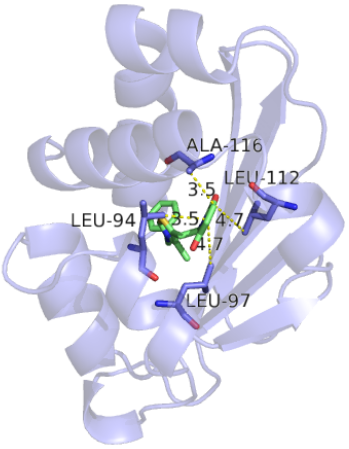<br>affinity -5.2 kcal/mol	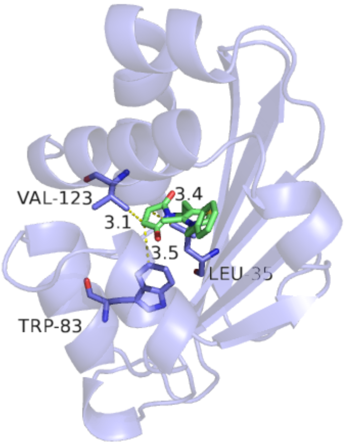<br>affinity -5.1 kcal/mol	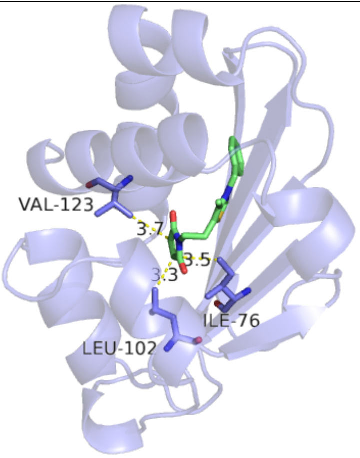<br>affinity -4.0 kcal/mol
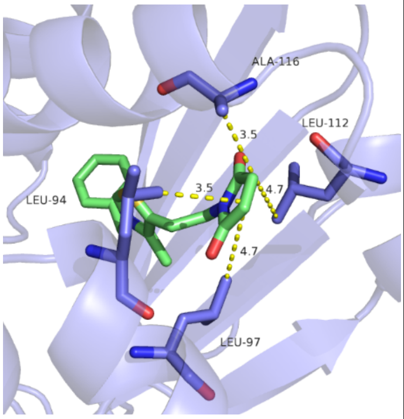	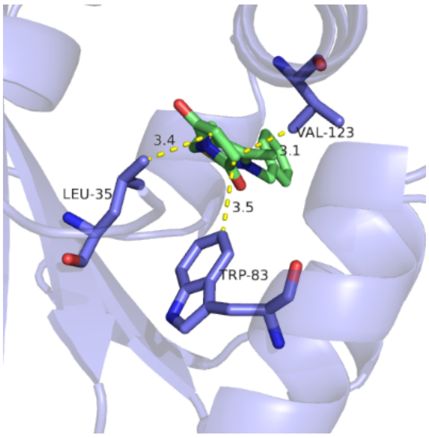	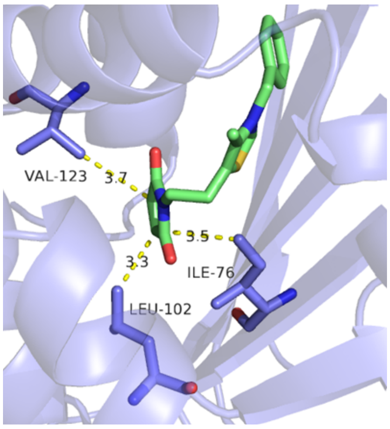

Models of the chosen mutants were created using Alphafold2 and the Breslow intermediate **(8)** was docked into these structures using Autodock Vina (**Table S5**). All models provided a feasible pose however, in most cases the maleimide was not close to the engineered cysteine residue. The exception was L102C pose 7 where the maleimide is <5 Å from Cys102. Although the affinities were poor and in many cases slightly positive, these poses indicated that there would be enough space within the cavity to contain the Breslow intermediate. We hypothesize that the weak,

Table S5 Affinities and structures of pose 1 generated by docking of the Breslow intermediate (**8**) into Alphafold models of each of the TTSCP cysteine mutants.

W83C

mode	affinity (kcal/mol)	dist from best mode rmsd l.b.	dist from best mode rmsd u.b.
1	7.4	0.000	0.000
2	7.7	1.463	2.443
3	7.8	1.445	2.548
4	9.5	2.400	8.251
5	10.2	1.730	2.891

Writing output ... done.

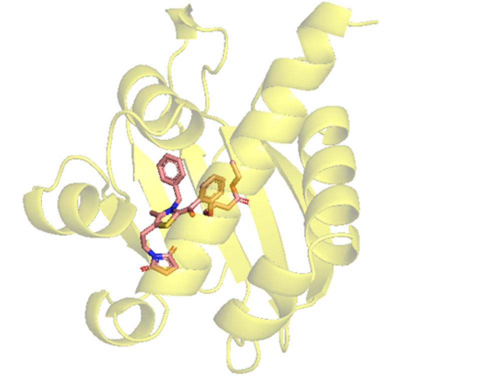

L102C

mode	affinity (kcal/mol)	dist from best mode rmsd l.b.	dist from best mode rmsd u.b.
1	-2.0	0.000	0.000
2	-2.0	0.430	1.479
3	-1.7	1.335	2.108
4	-1.3	2.402	8.742
5	0.1	4.068	8.584
6	0.2	1.564	2.294
7	0.2	2.830	9.466
8	0.6	1.940	3.042
9	0.8	3.574	8.767

Writing output ... done.

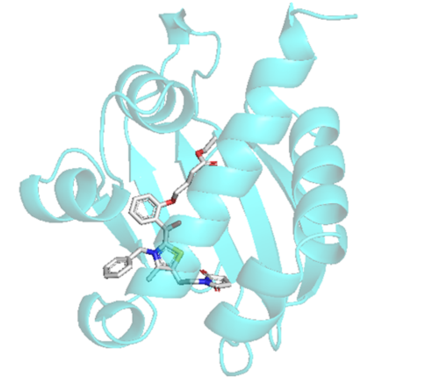

L112C

mode	affinity (kcal/mol)	dist from best mode rmsd l.b.	dist from best mode rmsd u.b.
1	-6.4	0.000	0.000
2	-5.6	2.408	7.823
3	-5.5	2.234	7.627
4	-5.2	1.251	2.097
5	-4.7	2.395	7.410
6	-3.4	2.868	7.694
7	-3.3	2.662	6.124
8	-3.2	2.320	5.841
9	-2.9	2.533	7.813

Writing output ... done.

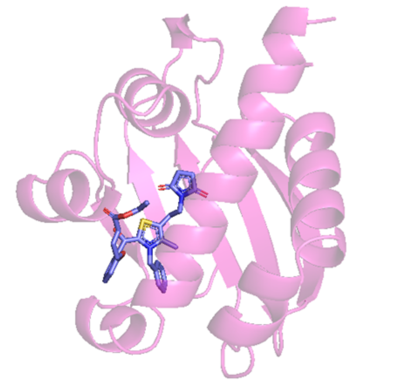

ΔDSB L102C

mode	affinity (kcal/mol)	dist from best mode rmsd l.b.	dist from best mode rmsd u.b.
1	-0.5	0.000	0.000
2	-0.4	0.915	1.565
3	-0.2	0.939	1.982
4	-0.1	1.950	3.870
5	1.5	3.074	5.401
6	1.7	1.151	1.648
7	1.8	2.085	3.901
8	2.8	2.138	3.614
9	3.4	2.117	3.640

Writing output ... done.

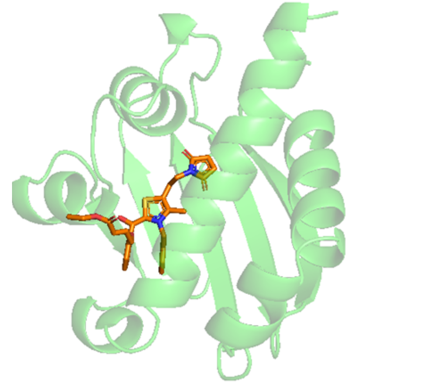

non-covalent binding of the Breslow intermediate is overcome by covalent modification of the cysteine residue by the maleimide thiazole. It is worth noting that in our hands no reversal of the maleimide conjugation has been observed.

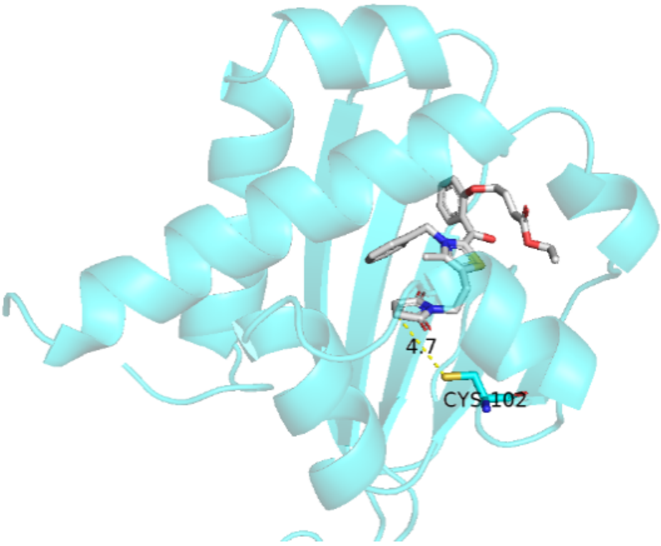

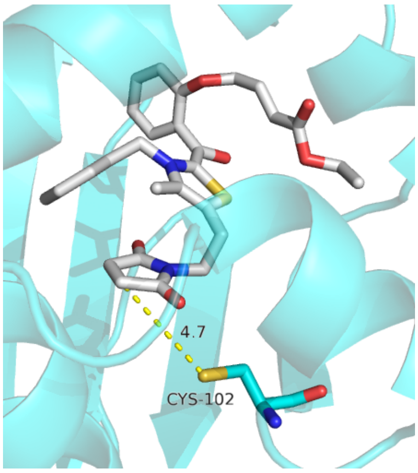

Affinity: 0.2 kcal/mol

***Figure S28*:** Pose 7 of the Breslow intermediate of **1** and **2** docked into TTSCP L102C Alphafold model shows plausible pose with maleimide 4.7 Å away from Cys102. Docking score gives a very slightly positive affinity of 0.2 kcal/mol.

Simulation Protocol

Simulation files were prepared using AmberTools[3], and simulations were performed with OpenMM[4]. For the non-bonded interactions, the Particle Mesh Ewald method was used with a cut-off of 1 nm. Hydrogen bond length constraints were applied. Simulations were run at 298 K and the temperature was kept constant using a Langevin integrator with a friction constant of 1 ps^{-1}. The system pressure was kept constant at 1 bar using a Monte Carlo barostat. Periodic boundary conditions were used. Timestep of the simulation was 2 fs, and information was collected from the simulation every 0.4 ns for a total of 500 frames. Root mean square deviation (RMSD), root mean square fluctuation (RMSF), radius of gyration (Rg) and surface accessible solvent area (SASA) analysis of the simulations were performed using the MDTraj library[5]. For the RMSD, RMSF and Rg analysis, the alpha carbon atom (Cα) of each residue was used. SASA analysis was carried out using all the backbone atoms. Pairwise RMSDs were calculated using the MDAnalysis package[6,7], using Cα atoms.

Individual steps taken to prepare the simulation files, to run the simulations, and to analyse the simulations can be found in the following repository: https://github.com/wells-wood-research/TTSCP_L102C_DSB-MBnThz-vs-TTSCP_L102C_DDSB-MBnThz

Simulation Analysis

To assess whether the removal of the disulphide bond between C13 and C60 causes a disruption to the protein structure in the presence of a C102 ligated MBnThz cofactor, we performed five replicates of a 200 ns molecular dynamics (MD) simulation for both TTSCP_L102C and TTSCP ΔDSB_L102C. Both RMSD and Rg analysis proposed that proteins did not show unfolding in the simulation time frame (Figure S29 A, C, Figure S30 A, C). RMSF analysis revealed that particularly residues around the cofactor (between 100-110) were slightly more flexible in TTSCP_L102C than in TTSCP ΔDSB_L102C (Figure S29 B, Figure S30 B), with fluctuations of approximately 1 Å. In addition, MBnThz in the case of TTSCP_L102C tended to be slightly more exposed to the solvent as the simulation proceeded, despite the overall SASA being similar for the entire protein in both cases (Figure S29 D, E, Figure S30 D, E). When

individually inspected, it was observed that MBnThz usually comes out of the pocket towards the solvent in TTSCP_L102C, whereas this behaviour was only present in one of the replicates of the TTSCP ΔDSB_L102C simulation (Figure S30 E). This behaviour is also accompanied by a slight opening of the distance between the helical portions containing residues 91-100 and 116-120 (Figure S29 F). Moreover, despite the overall deviation from the reference structure being similar in both conditions, TTSCP_L102C appeared to have higher flexibility between each time point, as suggested by the pairwise RMSD analysis (Figure S31). Nevertheless, the differences between TTSCP_L102C and TTSCP ΔDSB_L102C appear to be small and do not confer any drastic changes to the overall protein structure.

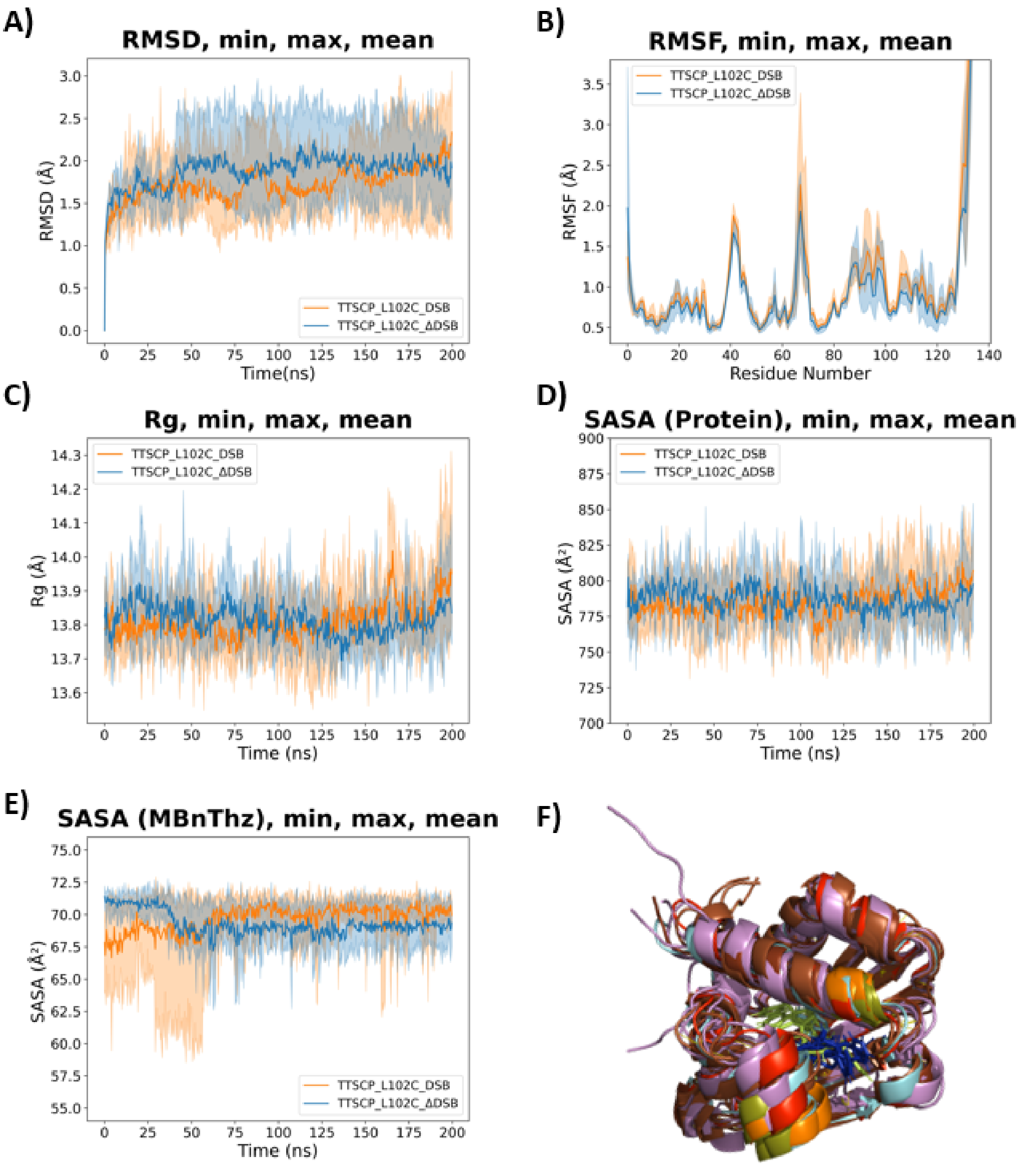

Figure S29: *Comparative MD analysis. A) RMSD compared to the starting structure over simulation time. B) RMSF of individual protein residues during simulation. C) Rg over simulation time. D) SASA of the entire protein over simulation time. E) SASA of MBnThz atoms over simulation time, this does not include cysteine atoms that are ligated to the cofactor. F) Alignment of TTSCP_L102C last frames (violet), TTSCP ΔDSB_L102C last frames (brown), TTSCP_L102C first frame (red), TTSCP ΔDSB_L102C (cyan), TTSCP_L102C 96-101 and 116-120 residues last frames (deep olive), TTSCP ΔDSB_L102C 96-101 and 116-120 residues (orange), MBnThz cofactor at the end of the TTSCP_L102C simulations (dark blue) and TTSCP ΔDSB_L102C (lime).*

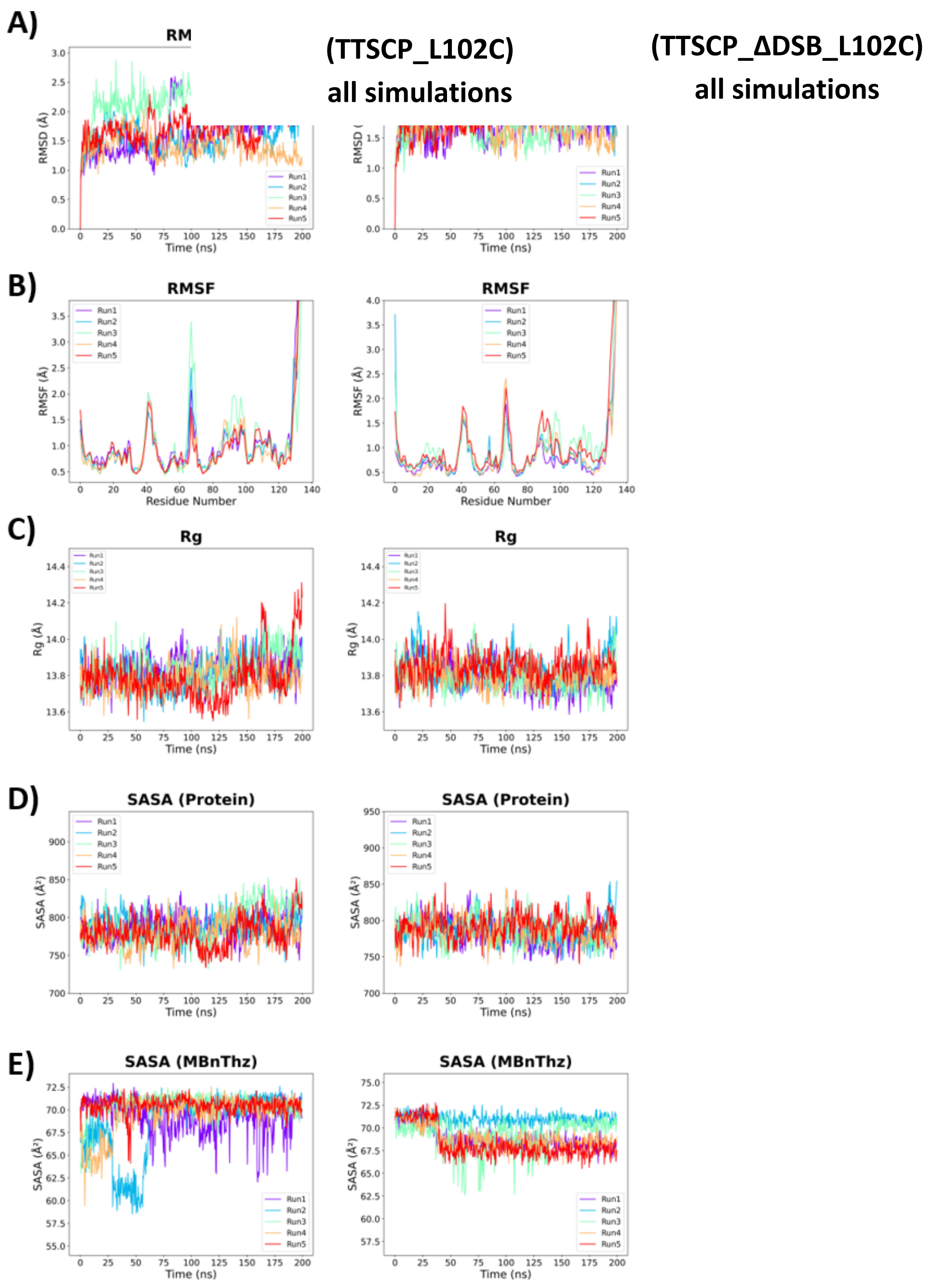

Figure S30: *Analysis of individual MD replicates. A) RMSD compared to the starting structure over simulation time. B) RMSF of individual protein residues during simulation. C) Rg over simulation time. D) SASA of the entire protein over simulation time. E) SASA of MBnThz atoms over simulation time, this does not include cysteine atoms that are ligated to the cofactor.*

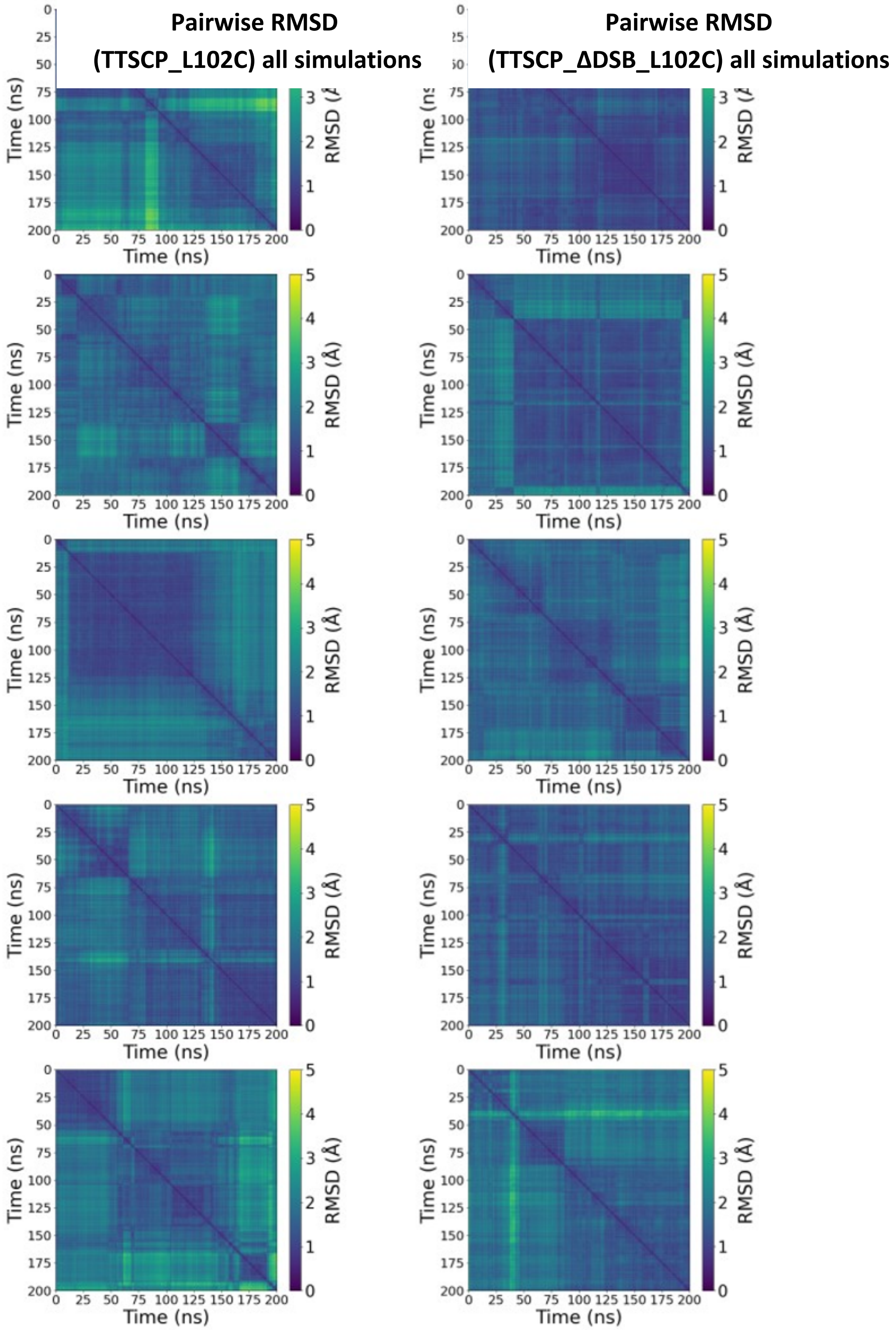

Figure S31*: Pairwise RMSD analysis of the individual MD simulation replicates for both TTSCP_L102C, and TTSCP ΔDSB_L102C*.

TTSCP melting temperature measurements

Data was collected on a Chirascan VX circular dichroism instrument, using a quartz “bottle” cell with a pathlength of 0.005 cm (Starna Scientific Ltd). The purified protein was buffer exchanged into 10 mM phosphate buffer, pH 7 using a PD-10 desalting column (Cytiva) and the concentration of the protein was adjusted to 0.3 mg/mL TTSCP, 0.15 mg/mL TTSCP L102C and 0.2 mg/mL TTSCP ΔDSB L102C. Before measuring the sample a baseline reading with 10 mM phosphate buffer, pH 7 was obtained (Figures S32-34). For each spectral run data was collected at each temperature over the wavelength range from 260 to 190 nm in 0.5-nm steps, and over the temperature range from 25 °C to 100 °C in 5 °C increments, with measurements at each temperature following a 60 s equilibration period. The spectral run was cooled to 25 °C after heating to 100 °C to assess refolding. Single wavelength melting curves were obtained by plotting the measured values of the 194 nm, 208 nm, 218 nm, 223 nm peaks of each spectrum against temperature (Figures S35-37). The data shows the T_m of TTSCP and TTSCP L102C has not been reached therefore T_m >95 °C whilst the data for TTSCP ΔDSB L102C shows the T_m to be 79 °C with analysis of the change in delta epsilon at 223 nm, 212 nm,208 nm and 194 nm all in agreement. This T_m is similar to that of human SCP-2L reported in the literature (T_m 80 °C).

Deuss, P. J. Artificial Metalloenzymes : Modified Proteins as Tuneable Transition Metal Catalysts, University of St Andrews, 2011. http://hdl.handle.net/10023/1923 (accessed 2023-03-08).

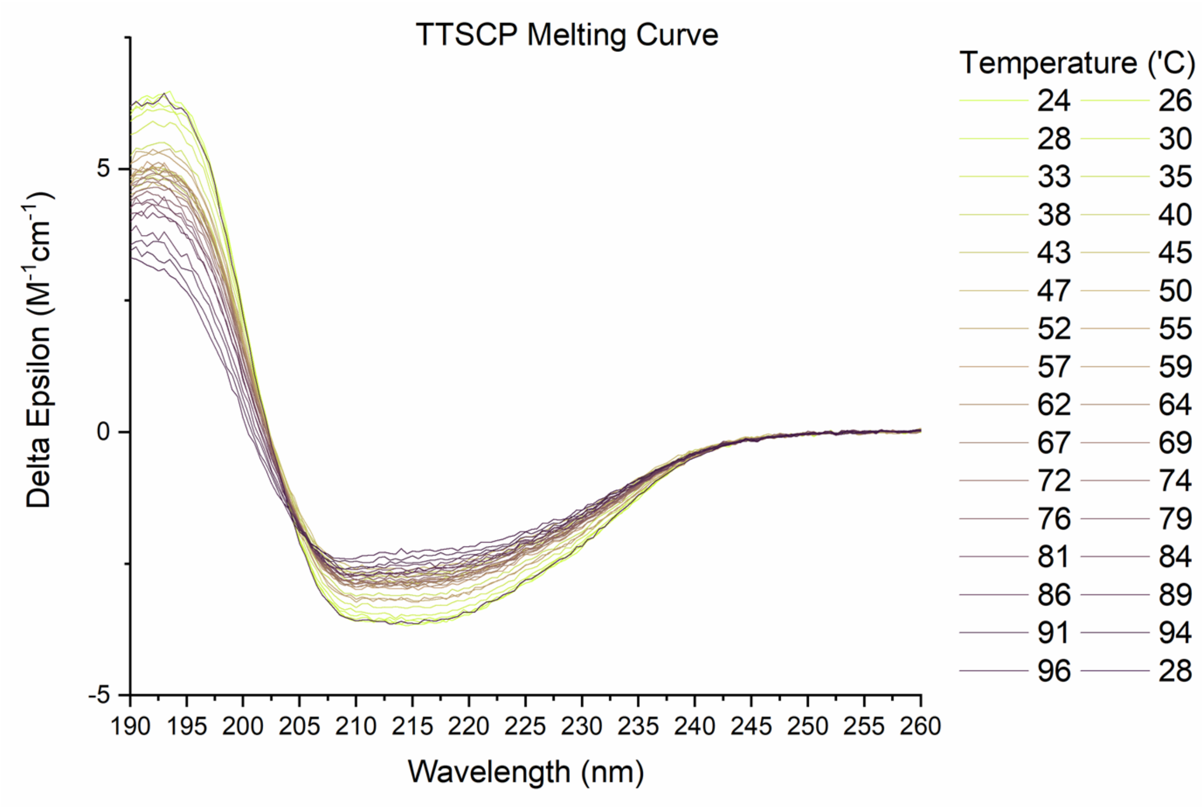

Figure S32: Melting curve of TTSCP recorded by CD.

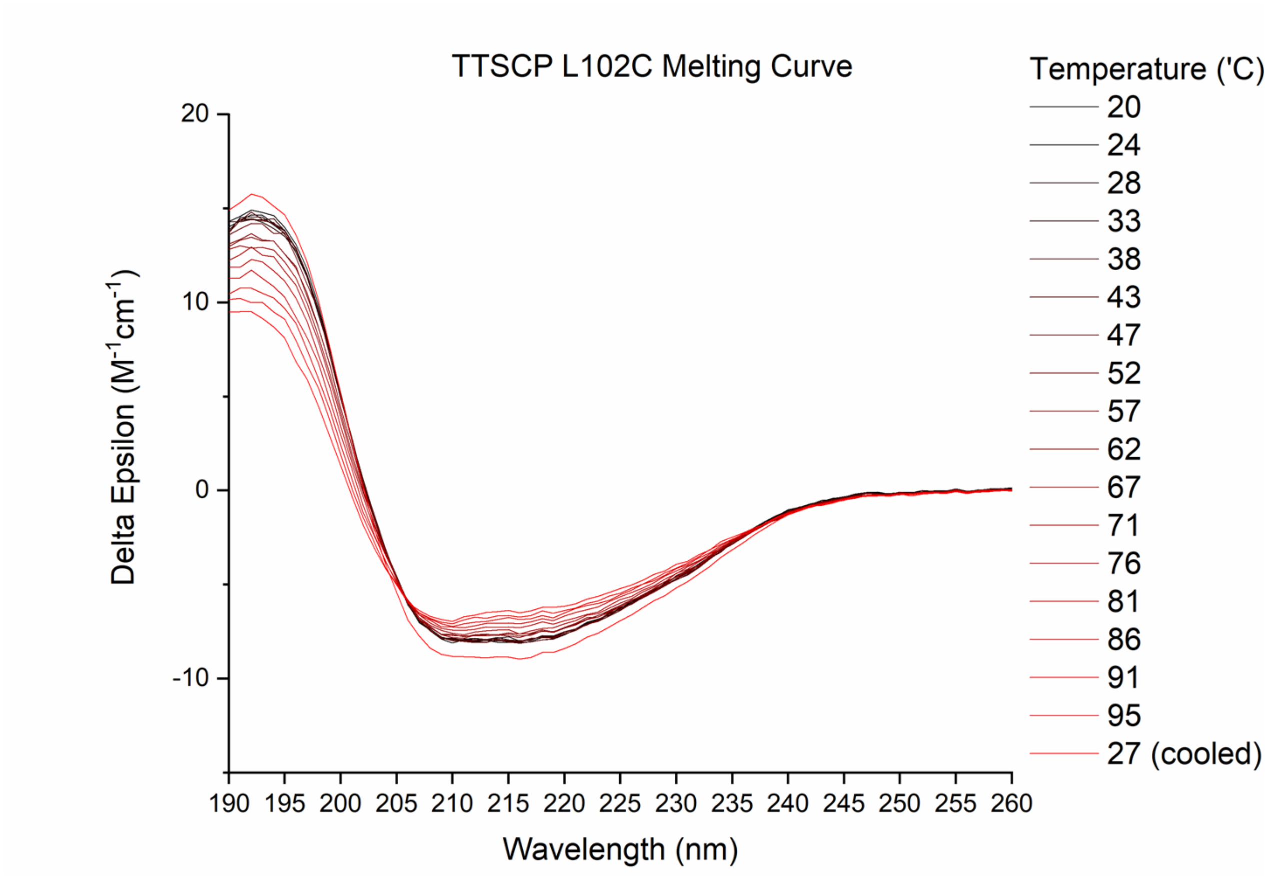

Figure S33: Melting curve of TTSCP L102C recorded by CD.

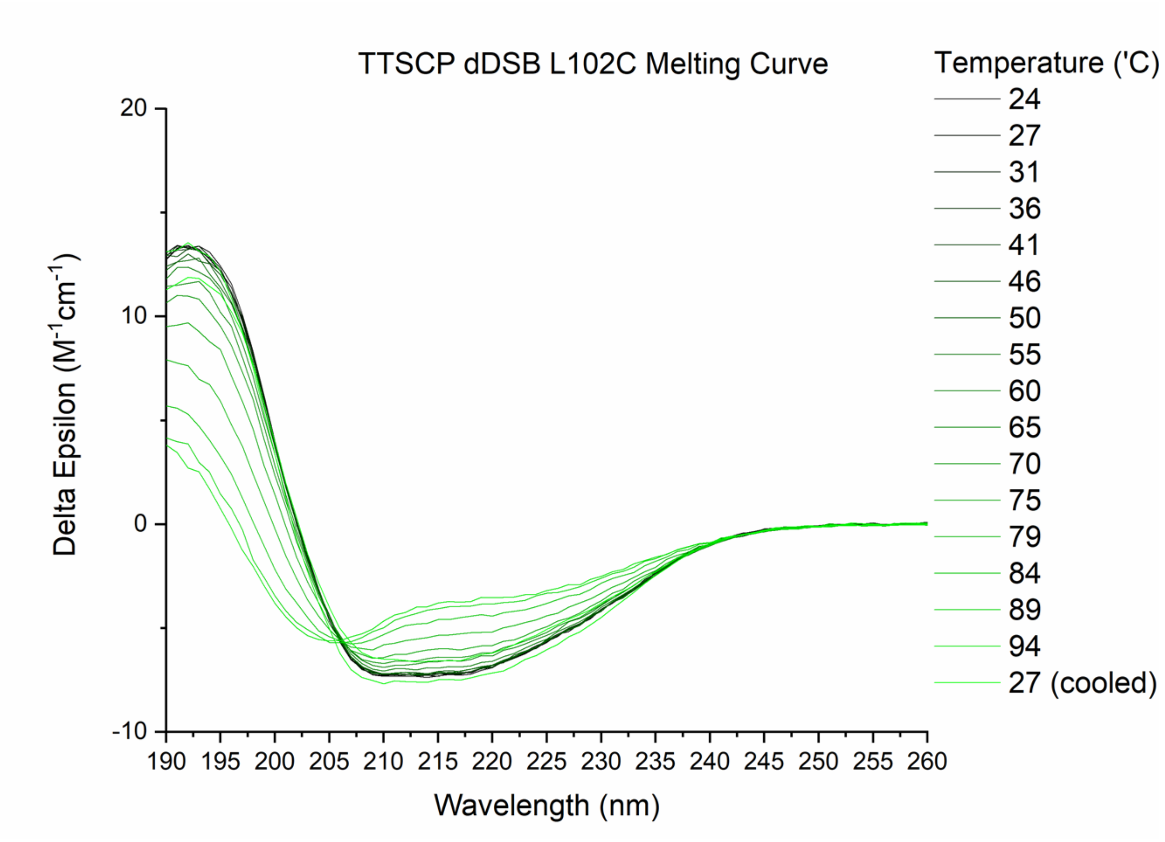

Figure S34: Melting curve of TTSCP ΔDSB L102C recorded by CD.

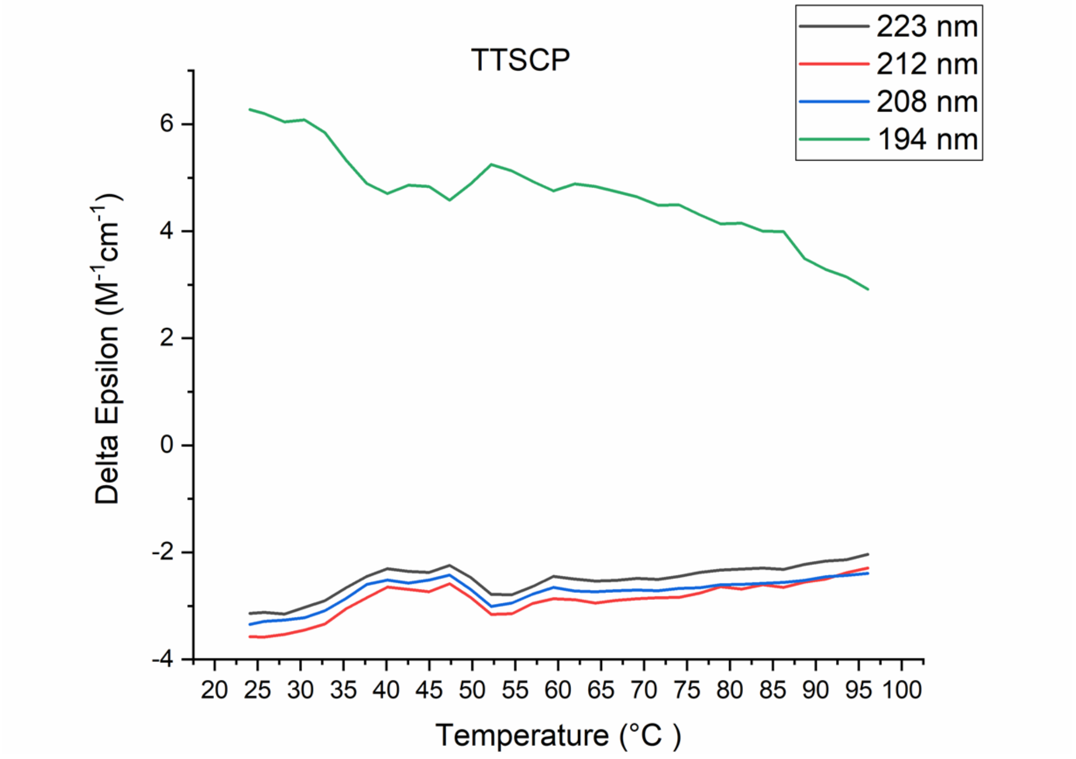

Figure S35: Plot of delta epsilon vs temperature for TTSCP at 223 nm (grey), 212 nm (red), 208 nm (blue) and 194 nm (green).

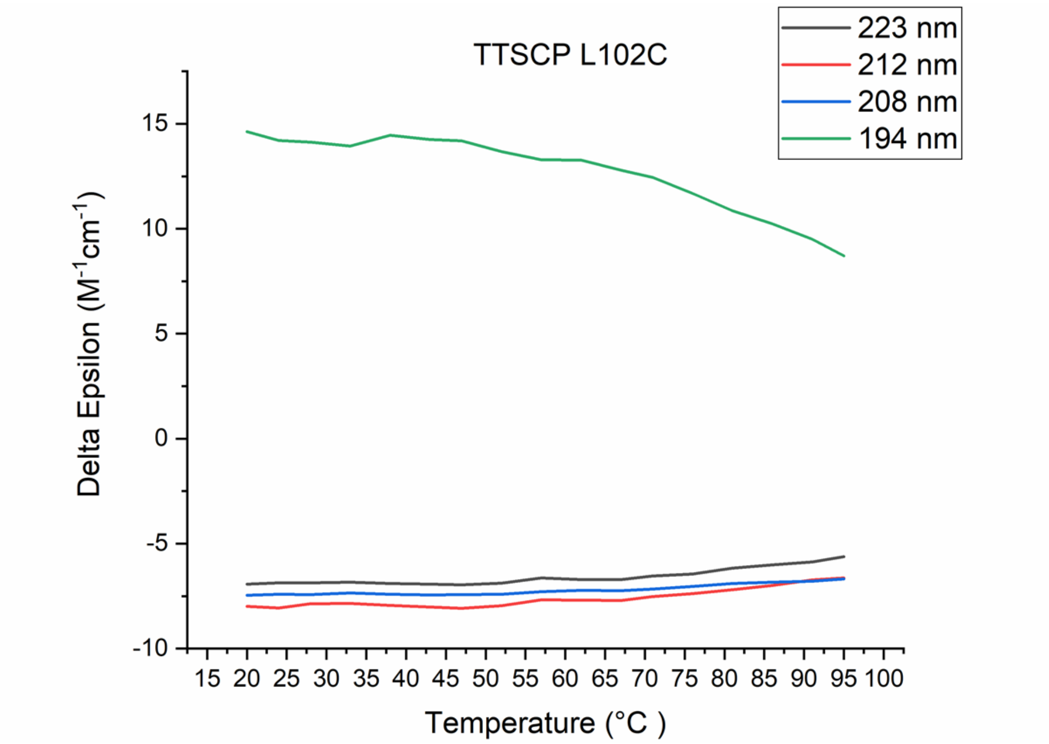

Figure S36: Plot of delta epsilon vs temperature for TTSCP L102C at 223 nm (grey), 212 nm (red), 208 nm (blue) and 194 nm (green).

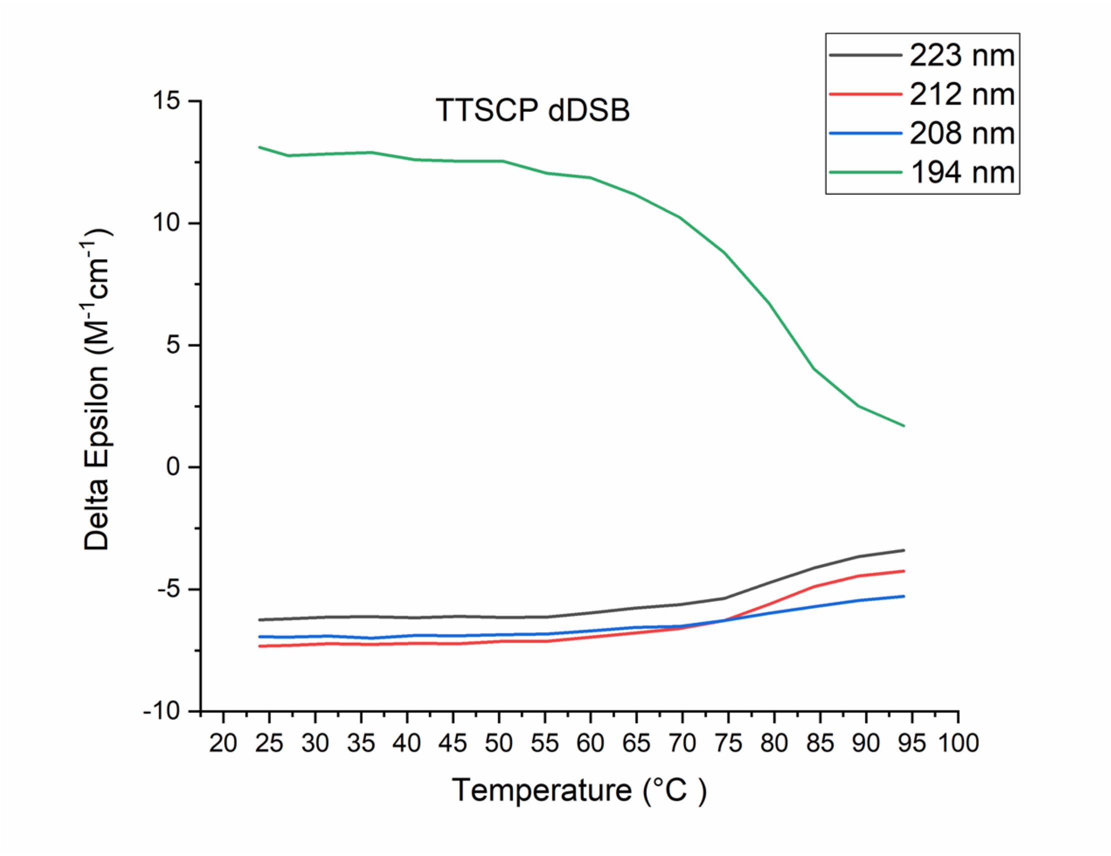

Figure S37: Plot of delta epsilon vs temperature for TTSCP ΔDSB L102C at 223 nm (grey), 212 nm (red), 208 nm (blue) and 194 nm (green).

NMR and MS Data

Compound (5)

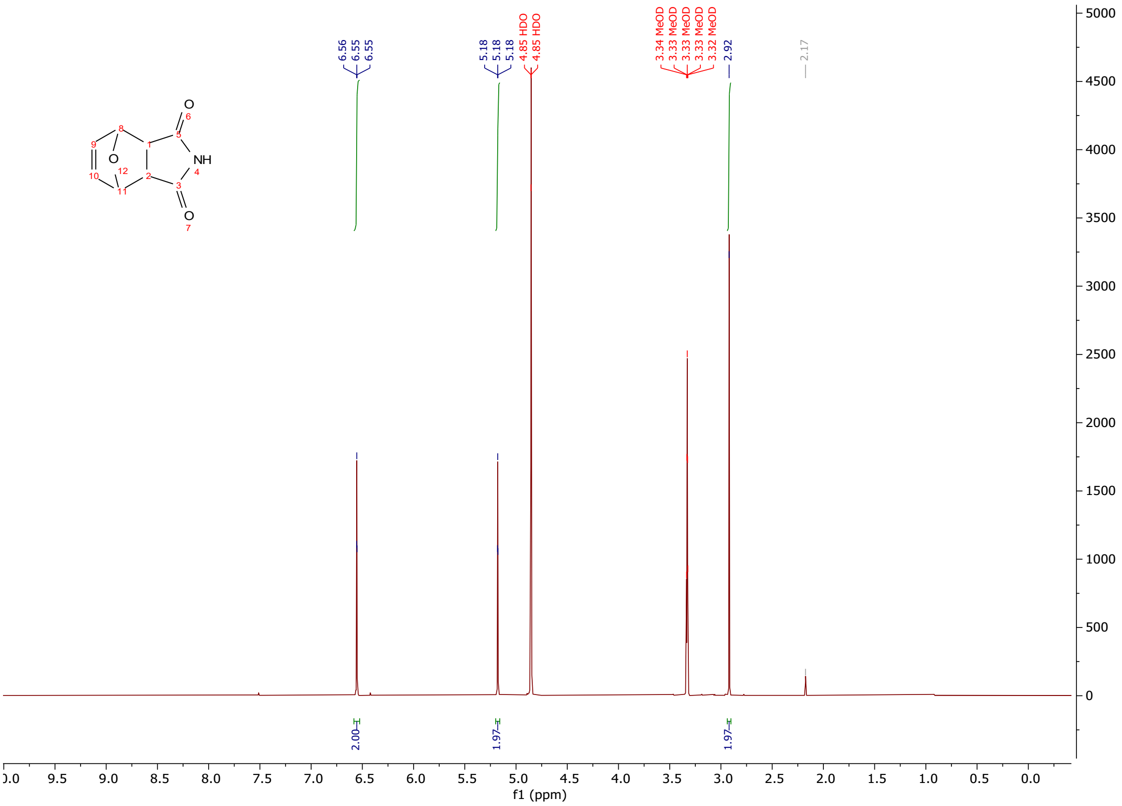

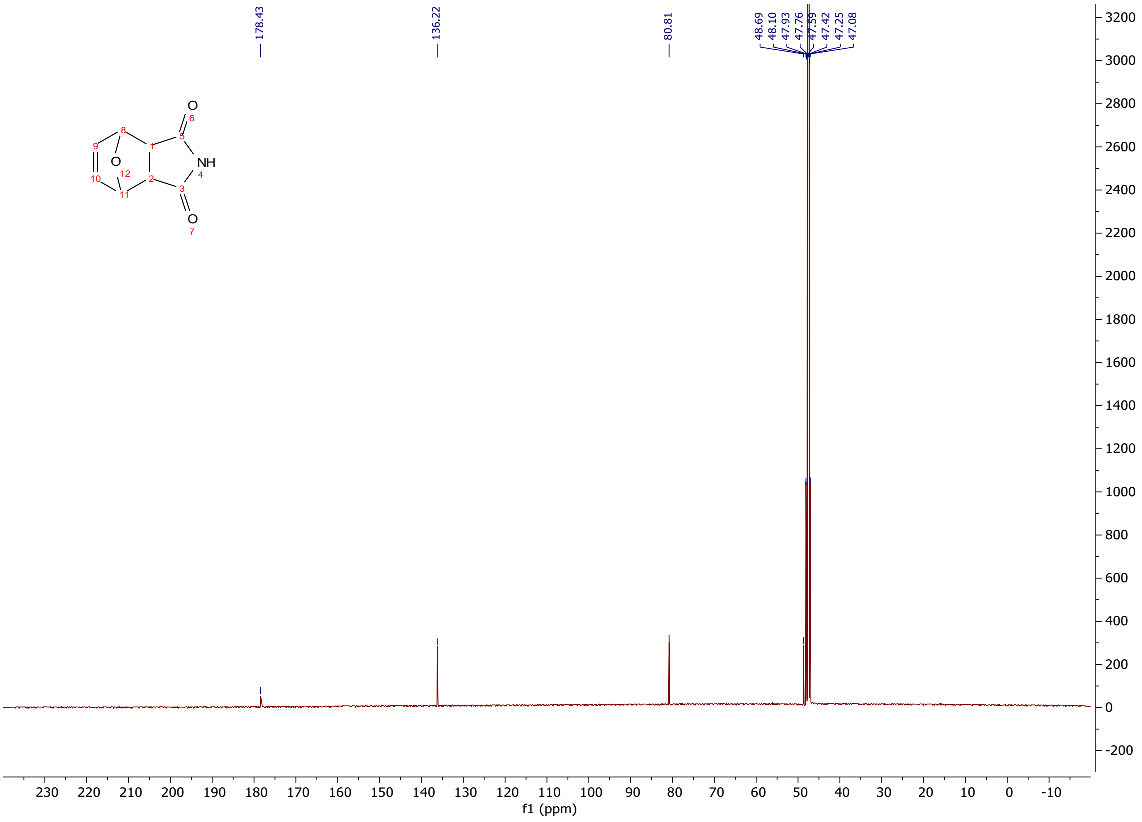

Acquisition Parameter

Source Type	ESI	Ion Polarity	Positive	Set Nebulizer	0.8 Bar
Focus	Not active			Set Dry Heater	200 °C
Scan Begin	50 m/z	Set Capillary	3500 V	Set Dry Gas	6.0 l/min
Scan End	3000 m/z	Set End Plate Offset	-500 V	Set Divert Valve	Waste

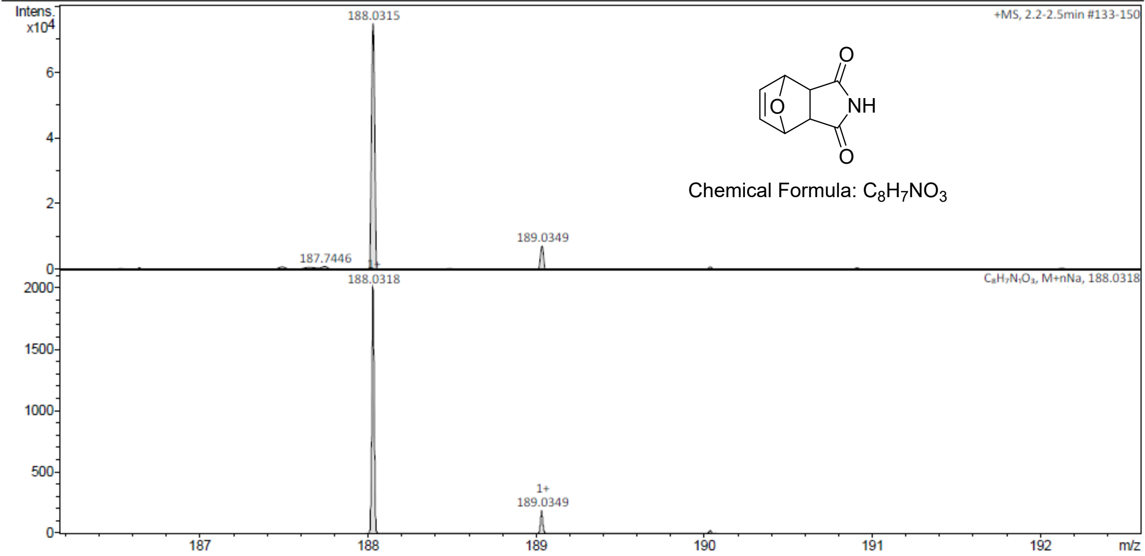

Compound (7)

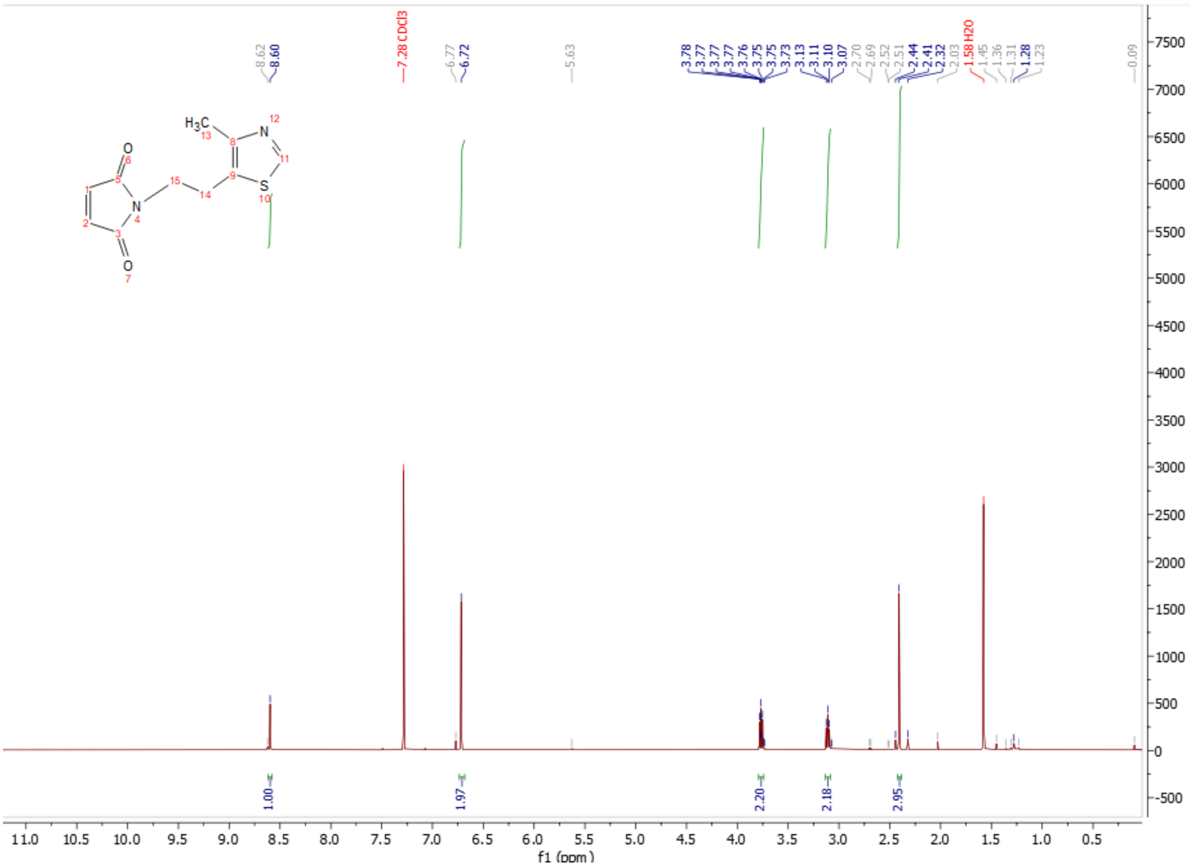
8.62
8.60
7.28 CDCl3
6.77
6.72
5.63
3.78
3.77
3.77
3.77
3.76
3.75
3.75
3.73
3.13
3.11
3.10
3.07
2.70
2.69
2.52
2.51
2.44
2.41
2.32
2.03
1.58 H2O
1.45
1.36
1.31
1.28
1.23
0.09
1.00
1.97
2.20
2.18
2.95
f1 (ppm)

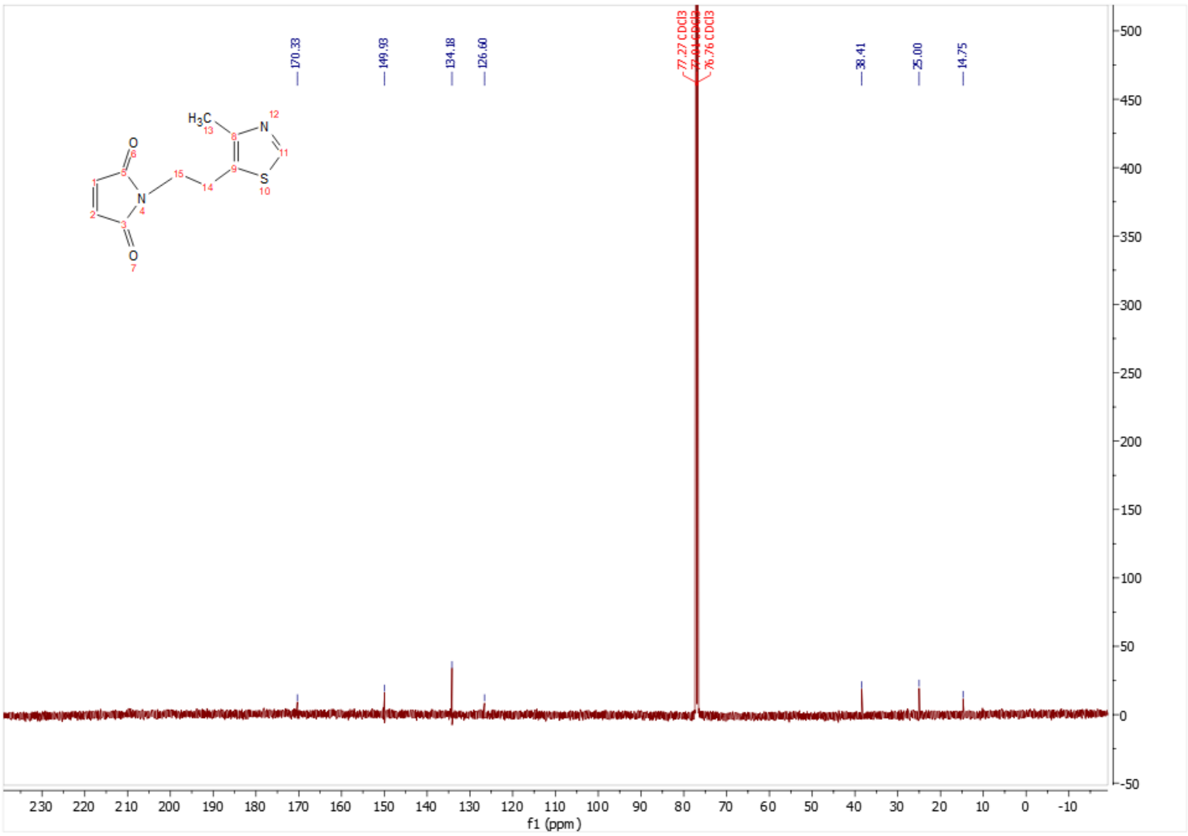
170.33
149.93
134.18
126.60
77.27 CDCl3
76.76 CDCl3
38.41
25.00
14.75
f1 (ppm)

MS (ESI)

Acquisition Parameter

Source Type	ESI	Ion Polarity	Positive	Set Nebulizer	0.8 Bar
Focus	Not active			Set Dry Heater	200 °C
Scan Begin	50 m/z	Set Capillary	3500 V	Set Dry Gas	6.0 l/min
Scan End	3000 m/z	Set End Plate Offset	-500 V	Set Divert Valve	Waste

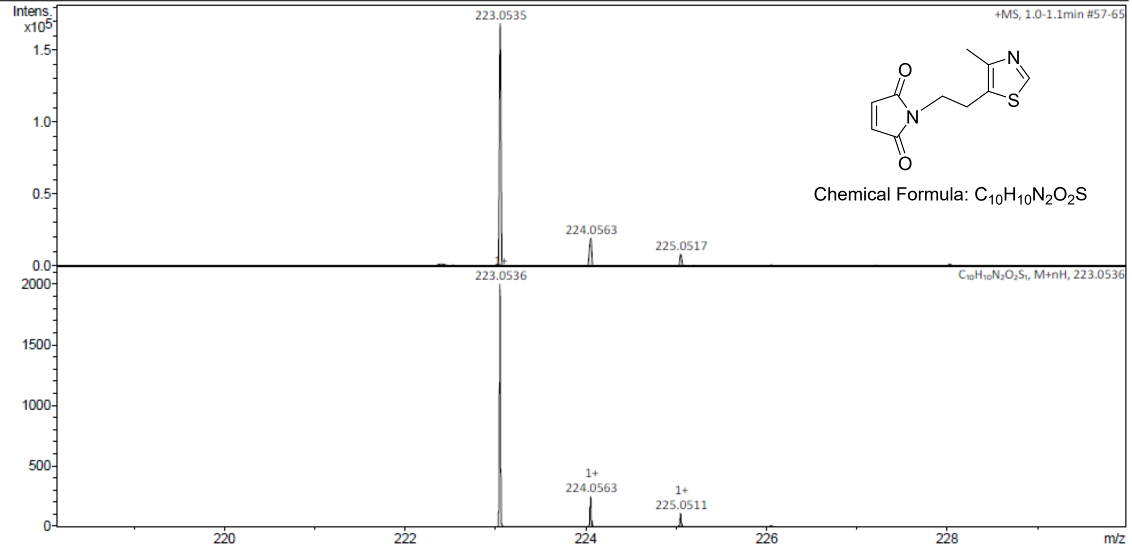

Compound (1)

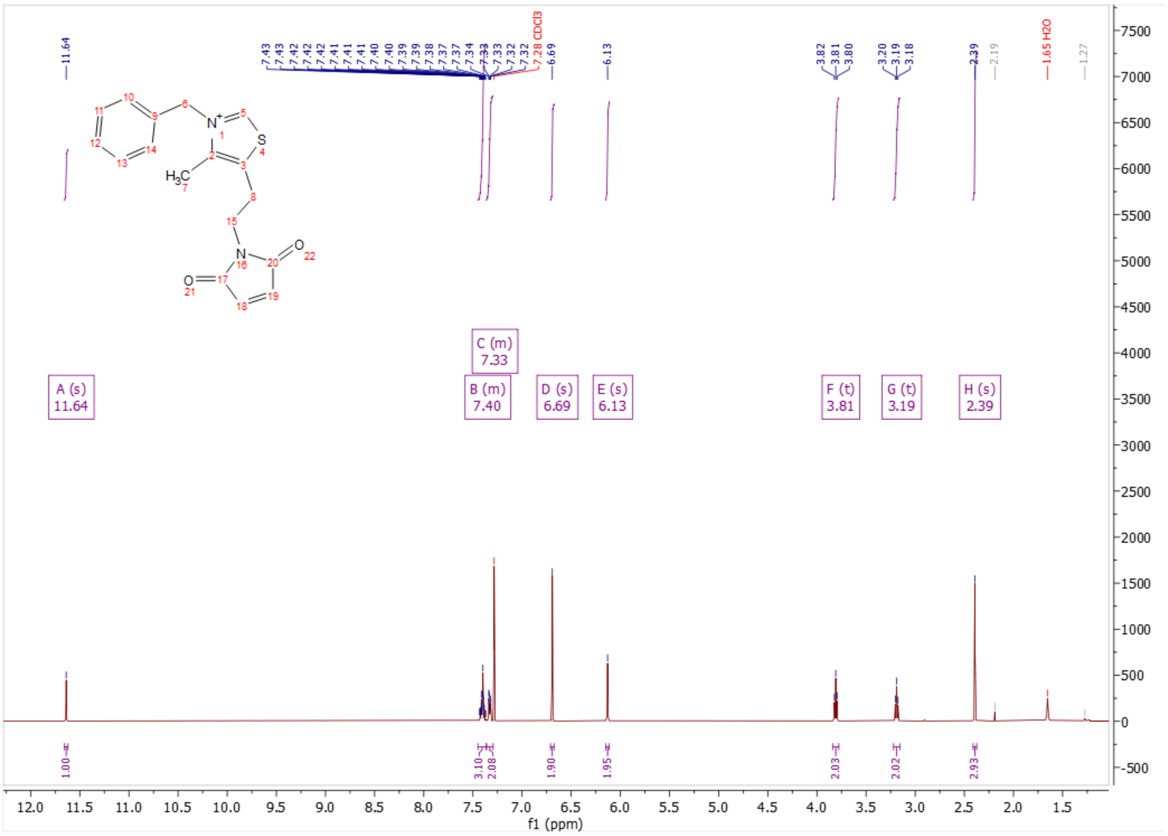

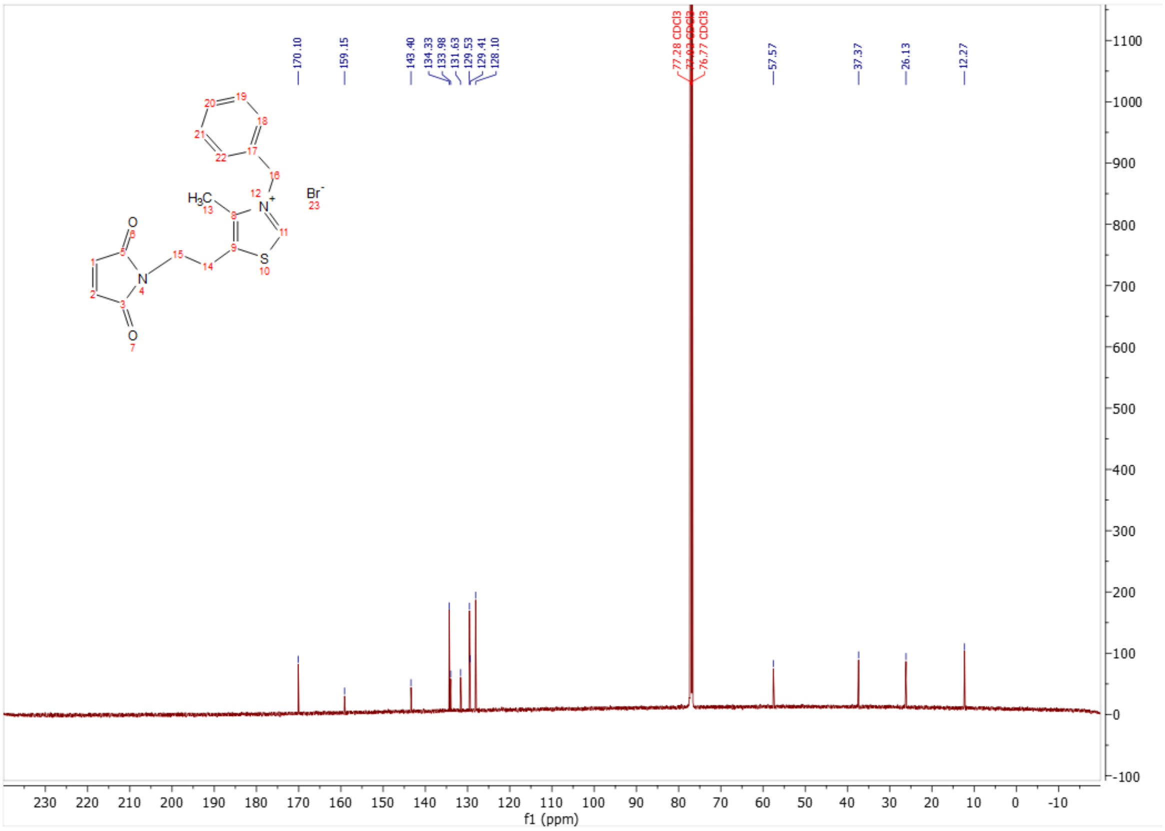

COSY

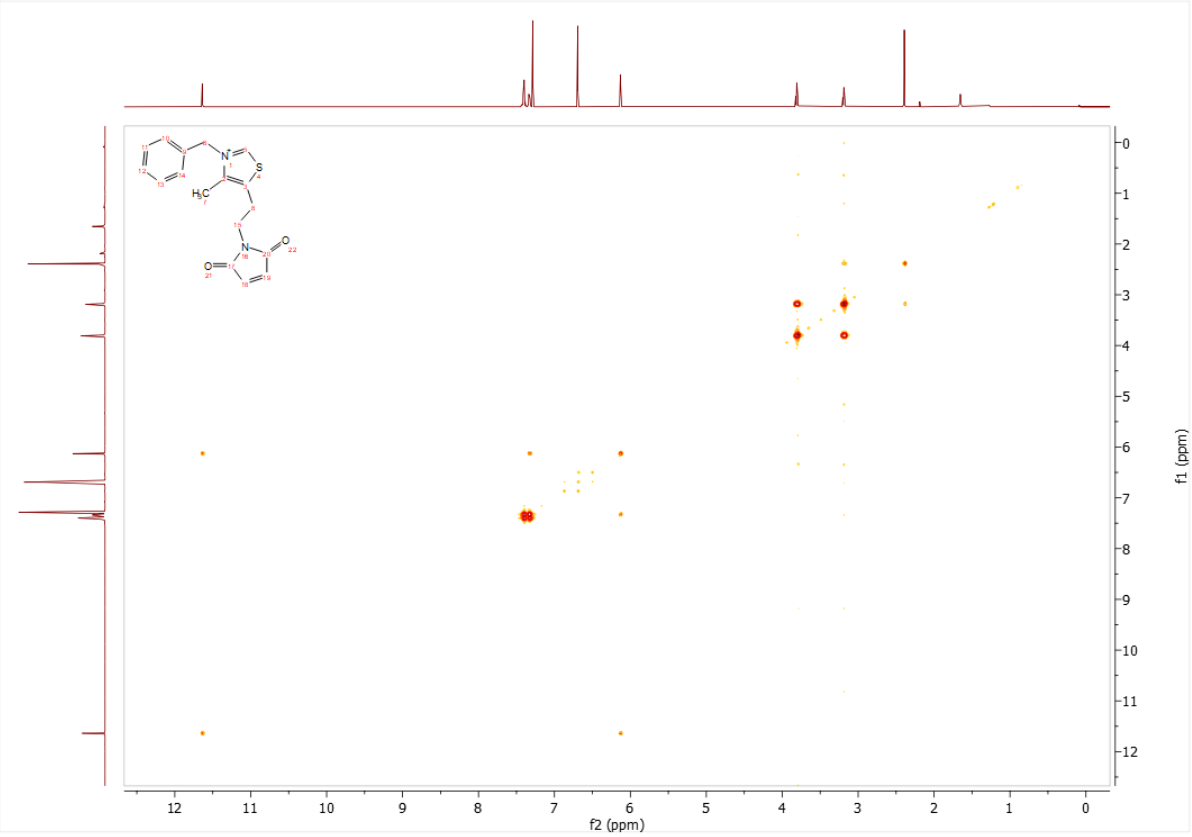

MS (ESI)

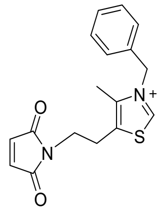

Chemical Formula: $C_{17}H_{17}N_2O_2S^+$

Molecular Weight: 313.39

m/z: 313.10 (100.0%), 314.10 (18.4%), 315.10 (4.5%), 315.11 (1.6%)

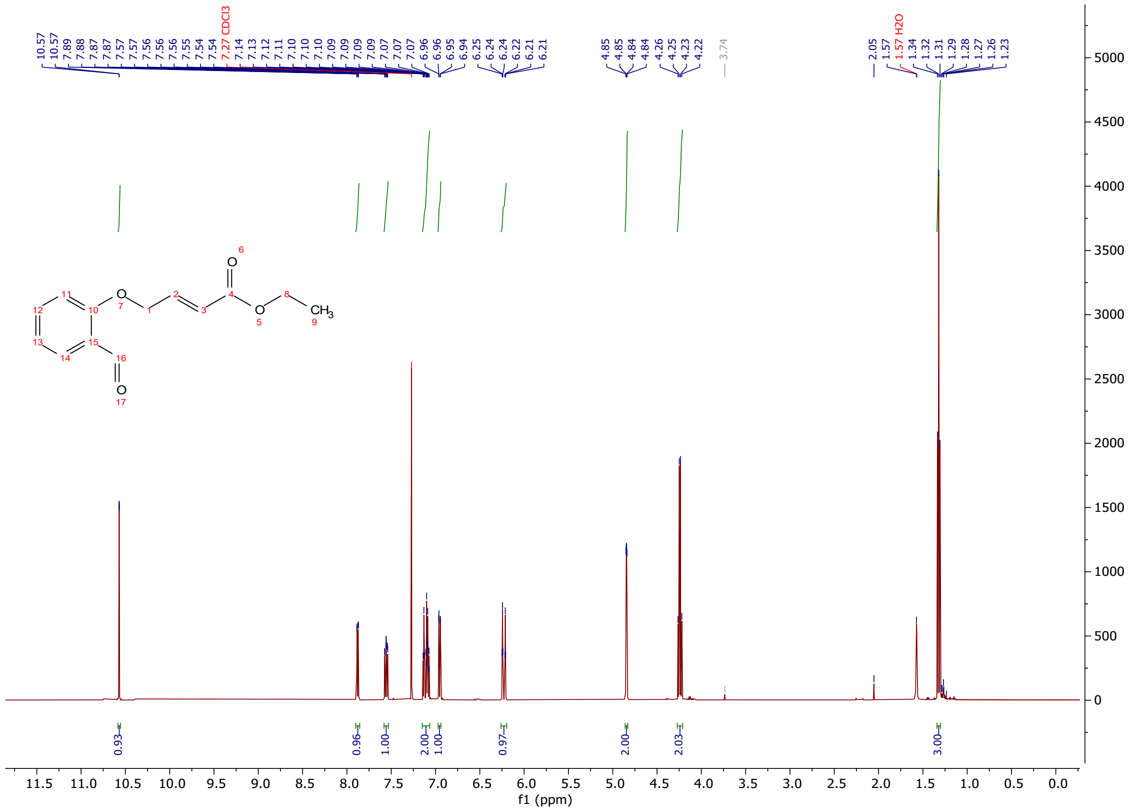

Compound (2)

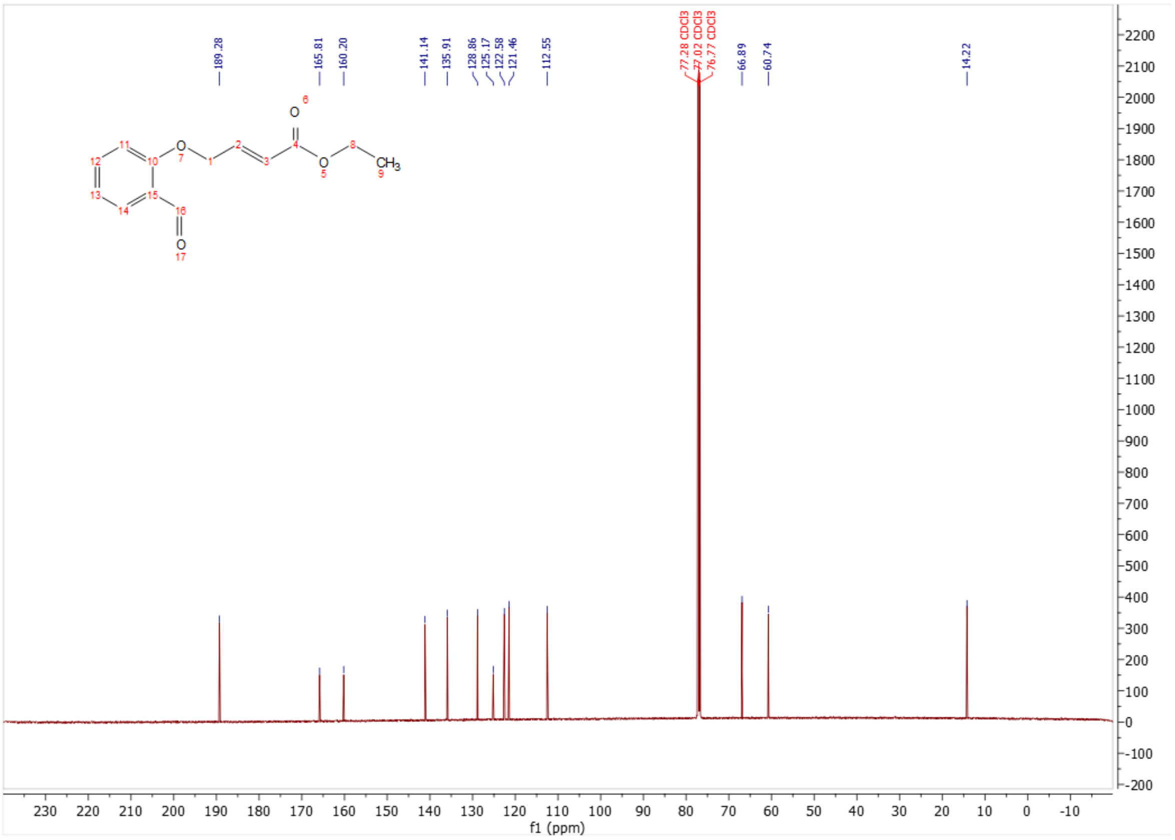

MS (ESI)

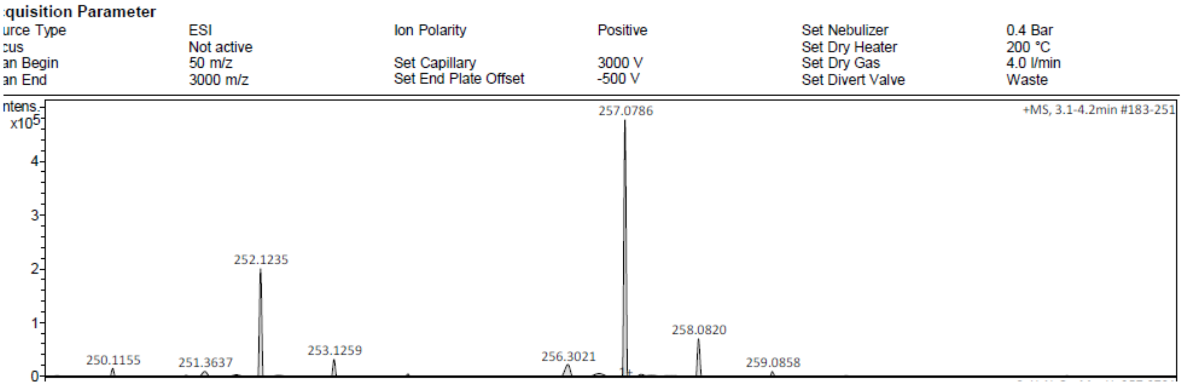
quisition Parameter
urce Type
cus
an Begin
an End
ESI
Not active
50 m/z
3000 m/z
Ion Polarity
Set Capillary
Set End Plate Offset
Positive
3000 V
-500 V
Set Nebulizer
Set Dry Heater
Set Dry Gas
Set Divert Valve
0.4 Bar
200 °C
4.0 l/min
Waste
ntens.
x10^5
+MS, 3.1-4.2min #183-251
257.0786
252.1235
258.0820
250.1155
251.3637
253.1259
256.3021
259.0858
0
1
2
3
4

Compound (3)

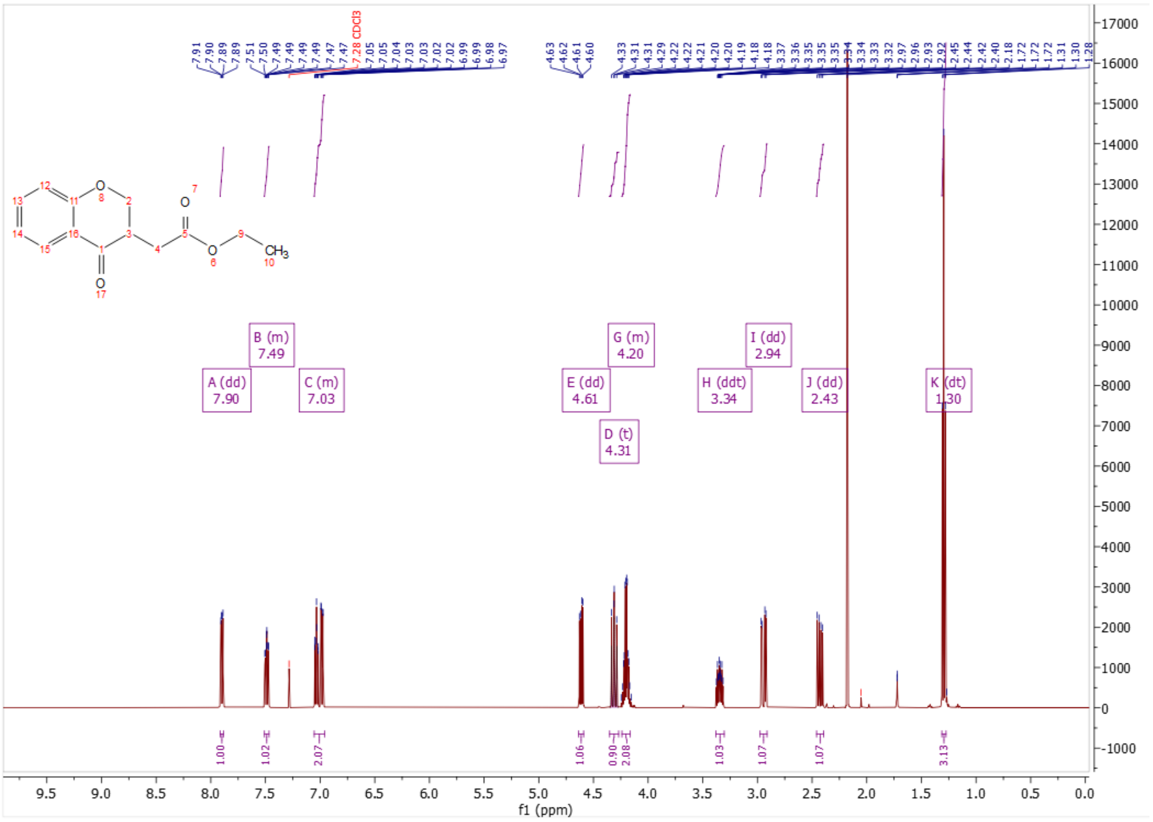
A (dd) 7.90
B (m) 7.49
C (m) 7.03
E (dd) 4.61
D (t) 4.31
G (m) 4.20
H (ddt) 3.34
I (dd) 2.94
J (dd) 2.43
K (dt) 1.30
f1 (ppm)

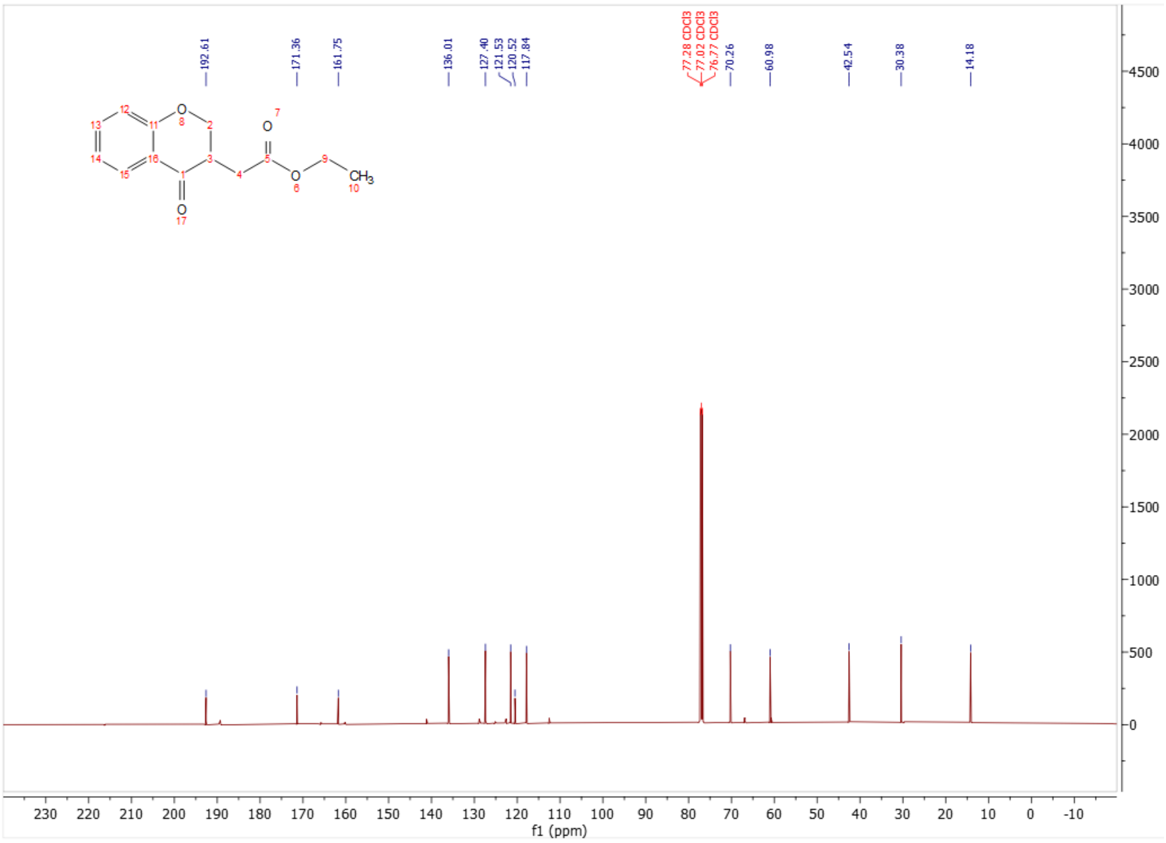
192.61
171.36
161.75
136.01
127.40
121.53
120.52
117.84
77.28 CDCl3
77.02 CDCl3
76.77 CDCl3
70.26
60.98
42.54
30.38
14.18
f1 (ppm)

Supplementary references

1. L. Gorecki, R. Andrys, M. Schmidt, T. Kucera, M. Psotka, B. Svobodova, V. Hrabcova, V. Hepnarova, P. Bzonek, D. Jun, K. Kuca, J. Korabecny and K. Musilek, *ACS Medicinal Chemistry Letters*, 2020, **11**, 65-71.
2. X. Chen, Z. Wang, Y. Lou, Y. Peng, Q. Zhu, J. Xu and Q. Wu, *Angew. Chem. Int. Ed. Engl.*, 2021, **60**, 9326-9329.
3. D.A. Case, H.M. Aktulga, K. Belfon, D.S. Cerutti, G.A. Cisneros, V.W.D. Cruz eiro, N. Forouzesh, T.J. Giese, A.W. Götz, H. Gohlke, S. Izadi, K. Kasavajhala, M.C. Kaymak, E. King, T. Kurtzman, T.-S. Lee, P. Li, J. Liu, T. Luchko, R. Luo, M. Manathunga, M.R. Machado, H.M. Nguyen, K.A. O'Hearn, A.V. Onufriev, F. Pan, S. Pantano, R. Qi, A. Rahnamoun, A. Risheh, S. Schott-Verdugo, A. Shajan, J. Swails, J. Wang, H. Wei, X. Wu, Y. Wu, S. Zhang, S. Zhao, Q. Zhu, T.E. Cheatham III, D.R. Roe, A. Roitberg, C. Simmerling, D.M. York, M.C. Nagan and K.M. Merz Jr., 2023, *J. Chem. Inf. Model.,* **63**, 6183-6191.
4. P. Eastman, J. Swails, J.D. Chodera, R.T. McGibbon, Y. Zhao, K.A. Beauchamp, L. Wang, A.C. Simmonett, M.P. Harrigan, C.D. Stern, R.P. Wiewora, B.R. Brooks and V.S. Pande, *PLoS Comput. Biol.*, 2017, **13(7)**, e1005659.
5. R.T. McGibbon, K.A. Beauchamp, M.P. Harrigan, C. Klein, J.M. Swails, C.X. Hernández, C.R. Schwantes, L.P. Wang, T.J. Lane and V.S. Pande, *Biophysical Journal*, 2015, **109**, 1528-1532.
6. N. Michaud-Agrawal, E.J. Denning, T.B. Woolf and O. Beckstein, *J. Comput. Chem.*, 2011, **32**, 2319-2327.
7. R.J. Gowers, M. Linke, J. Barnoud, T.J.E. Reddy, M.N. Melo, S.L. Seyler, D.L. Dotson, J. Domanski, S. Buchoux, I.M. Kenney and O. Beckstein, In S. Benthall and S. Rostrup, editors, *Proceedings of the 15th Python in Science Conference*, 2016, 98-105.
